# A State-of-the-Art Review of Polymer-Enabled Bionic Vascular Self-Healing Cementitious Materials: Vascular Fabrication, Healing Agent Use, and Healing Efficiency Evaluation

**DOI:** 10.3390/polym18151889

**Published:** 2026-07-31

**Authors:** Xianfeng Wang, Dongwei Zhang, Xuanzhe Zhang

**Affiliations:** 1 Guangdong Provincial Key Laboratory of Durability for Marine Civil Engineering, College of Civil and Transportation Engineering, Shenzhen University, Shenzhen 518060, China; xfw@szu.edu.cn (X.W.);; 2 Shenzhen Key Laboratory for Low-Carbon Construction Material and Technology, Shenzhen 518060, China; 3 State Key Laboratory of Intelligent Construction and Healthy Operation and Maintenance of Deep Underground Engineering, Shenzhen University, Shenzhen 518060, China

**Keywords:** vascular self-healing, cementitious materials, polymer composites, healing agents, sustainable infrastructure

## Abstract

This review provides an overview of the latest advances in bionic vascular self-healing cement, focusing on vascular design, fabrication, selection of healing agents, transport and curing mechanisms, and performance evaluation methods. Compared to systems based on microcapsules and microorganisms, vascular networks enable directed and efficient transport of healing agents and repeated healing; however, the presence of hollow channels results in an inevitable loss of mechanical properties. Additive manufacturing, in situ printing based on Pickering emulsions, and direct printing of cement-based or multi-material systems have enhanced geometric flexibility and scalability. However, issues such as channel quality, polymer-cement interface stability, and on-site quality control remain unclear. Regarding the selection of healing agents, epoxy resin systems are generally more suitable for structural healing, polyurethanes are suitable for rapid sealing and wide or irregular cracks, while silicate healing agents are suitable for healing where cement compatibility and durability are prioritized. The most critical research gap lies in the lack of standardized, full-scale, multi-cycle, and long-term environmental validation, which limits the practical engineering application of vascular self-healing technology. Future research should prioritize the integrated design of various performance metrics, the long-term durability of polymers, standardized benchmark testing, and validation based on actual service conditions.

## 1. Introduction

Concrete is considered the most widely used building material in the world today; however, its production process is accompanied by significant carbon emissions [1,2]. According to a report by the International Energy Agency (IEA), global cement production accounts for approximately 7% of total carbon dioxide emissions. Concrete design optimization is crucial to achieving carbon reduction goals and promoting sustainable development. From the perspective of extending the service life of structures and reducing carbon emissions throughout their life cycle, self-healing concrete has attracted considerable attention [3,4]. This technique can automatically heal micro-cracks as they appear, preventing them from spreading and deteriorating the structure, while also avoiding the intrusion of corrosive ions, thereby extending the service life of buildings and reducing maintenance and repair needs [5,6]. In this context, vascular self-healing also intersects with polymer-modified cementitious materials and polymer composites, as polymeric channels, templates, and healing agents can influence crack transport, interfacial bonding, and long-term durability.

Based on their healing mechanisms, self-healing concrete can be divided into two main categories: intrinsic and extrinsic self-healing. Intrinsic self-healing relies on insufficiently hydrated cement particles continuing to react with water without external intervention to generate hydration products that fill cracks [7]. This method is simple to implement and requires only adjustments to the concrete formula and mix ratio, but it is generally only effective for small cracks. Extrinsic self-healing involves introducing additional repair systems into the concrete, such as microcapsules, microorganisms, and vascular systems, to heal cracks, as well as the recently proposed practical application of self-healing cement, which stimulates self-healing on the concrete surface through spraying microorganisms that precipitate alkali-tolerant calcite [8]. Microcapsule self-healing techniques rely on the formation of cracks in the capsule wall, which tear the capsule and release a healing agent to seal the cracks [9]. However, this technique is limited by the uncertainty of single healing events and crack development paths [10,11]. Microbial self-healing leverages the metabolic processes of microorganisms to generate healing products [12,13]; however, many limitations remain in production conditions and long-term survival [14,15].

Although existing microcapsule and microbial techniques can repair concrete cracks to some extent, there is still room for improvement in healing range and effectiveness due to factors such as proportions, microcapsule dosage [16], and microbial metabolic rates [17]. In comparison, vascular self-healing shows greater potential, first proposed by Dry et al. [18], who suggested embedding glass tubes in concrete to distribute healing agents, marking an important step toward advanced self-healing systems. Subsequently, researchers drew inspiration from the biological vascular system and developed a technique to install a vascular network in concrete to deliver healing agents to damaged areas, enabling multi-cycle healing and restoring structural durability. Furthermore, the vascular phase not only functions as a transport pathway but can also be viewed as an embedded polymer or hybrid-embedded structure. The compatibility of this phase with the cementitious matrix directly influences load transfer, crack propagation, and material durability.

Currently, numerous studies have been published in the field of vascular self-healing, but only a few have reported reviews. Existing reviews have provided detailed explanations of vascular design principles, manufacturing strategies, fracture responses of concrete vascularization, and potential applications of vascular cement [19]. However, due to recent advances in the literature, new approaches have emerged in manufacturing technology, including 3D-printed concrete, carbonization-enhanced compensation for compressive strength, and synergistic printing for toughening. In addition, composite-related reviews provide a comprehensive overview of healing agents, but they are not detailed enough from the perspective of cementitious materials. This is reflected in the influence of the cement environment on the diffusion and curing of the healing agent, as well as in differences in the effectiveness of various repairs due to variations in their properties. In response, this paper provides an overview based on new literature from the past three years (57% of the total references). First, it reviews the design principles and manufacturing strategies for vascular self-healing cement and summarizes the latest advancements in this field. Next, it explains the mechanisms of action of healing agents, summarizes the healing effects of different agents, and introduces methods for evaluating healing efficiency applicable to vascular self-healing strategies. Finally, it summarizes the challenges in vascular self-healing methods and discusses the field’s future development based on recent research.

## 2. Design of Vascular Systems in Cementitious Materials

Design principles and fabrication techniques based on vascular systems are reviewed. Strategies for applying design principles, artificial intelligence (AI)-assisted techniques, and fabrication methods are analyzed in terms of their strengths, weaknesses, and applicability. Updates are provided on the latest vascular fabrication strategies, in particular the application of neural networks, in situ printing techniques based on Pickering emulsions, and three-dimensional printing cements. The design principles and assistive methods for self-healing vascular systems are summarized in Table 1.

### 2.1. Design Principles of Vascular Systems

Before integrating a vascular system into a concrete structure, the vascular design must be fully developed. This examination should encompass the determination of the appropriate volume fraction of the vascular system, the pipe diameter, branching, and the grid structure. It is imperative to take into account Murray’s law, construction laws, computer-aided design, and compatibility issues between vascular and concrete. When polymeric channels are used, surface wettability, stiffness mismatch, and alkaline stability should also be considered, together with geometric parameters.

#### 2.1.1. Murray’s Law


(1) $${r_{p}}^{3}={r_{d_{1}}}^{3}+{r_{d_{2}}}^{3}$$

Murray’s Law is a biometric principle concerned with the branching angles of arteries, aiming to minimize energy loss during fluid transport. The fundamental principle is illustrated by Equation (1), which states that the cube of the parent pipe radius is equivalent to the sum of the cubes of all child pipe radii [20,21]. The continuous advancement of self-healing vascular technology has led to the development of biomimetic vascular systems in concrete, based on Murray’s law. These systems mimic the vascular network in living organisms, thereby enabling the more efficient distribution of healing agents to damaged areas. The design has been demonstrated to enhance repair efficiency, extend the material’s service life, and reduce maintenance costs.

**Figure 1:**
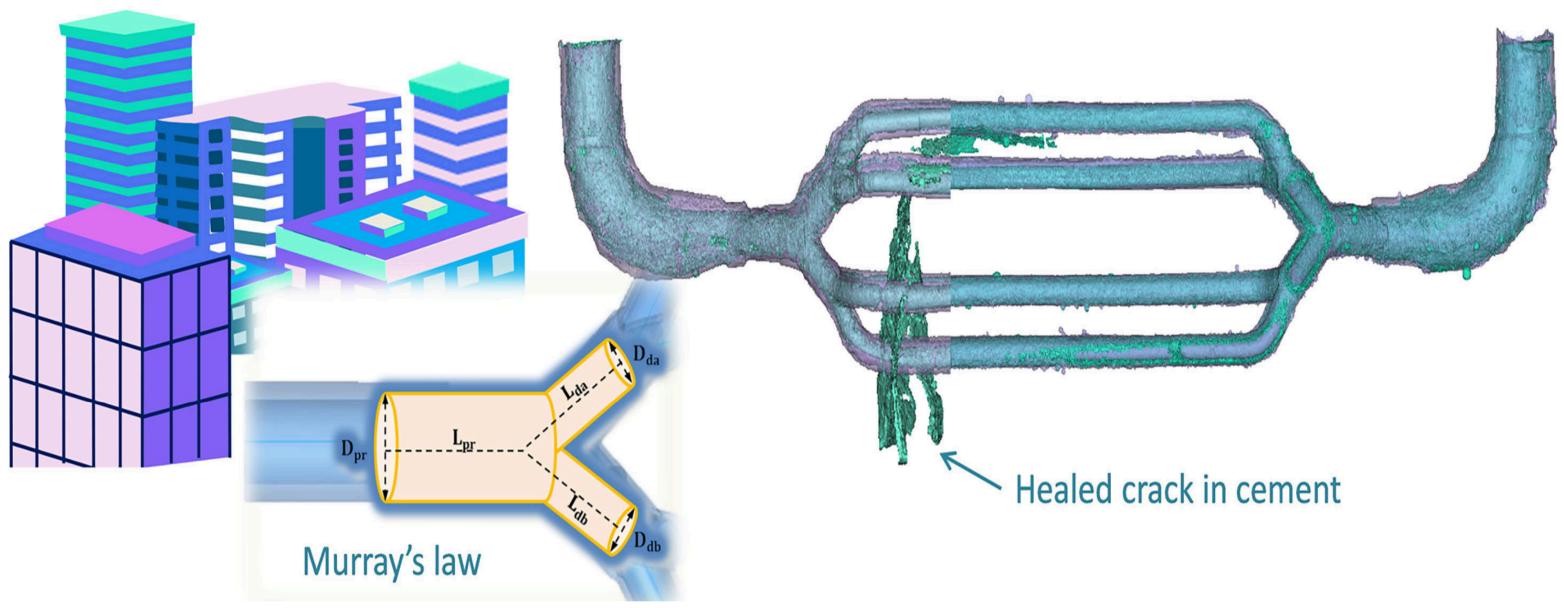
Schematic diagram of vascular branching considering Murray’s law [22].

As demonstrated in Figure 1, Li et al. [22] implemented Murray’s law in their research on 3D printing of vascular systems to optimize fluid transport routes, thereby enhancing the distribution range and healing efficiency of healing agents. Wan et al. [23] manufactured two-dimensional (2D) and three-dimensional (3D) vascular networks. The diameter of the vascular networks was designed according to Murray’s law to minimize turbulence at the junctions, optimize liquid transport efficiency, and provide a feasible reference for vascular self-healing in terms of energy and structural optimization.

The three-dimensional pipe system, designed according to Murray’s law, significantly improves healing efficiency compared with traditional two-dimensional vascular systems. The results show that applying Murray’s law to vascular design is an effective strategy. However, the ideal conditions for Murray’s law originate in biological systems, which differ from the heterogeneous, unpredictable, complex microstructures of cement materials [34]. Currently, there is no research on applying Murray’s law to concrete vascular systems, particularly regarding the flow behavior at irregular interfaces and randomly distributed cracks [35,36]. This suggests that there are still considerable research opportunities to apply Murray’s law to predict the optimal flow path in vascular concrete.

#### 2.1.2. Construction Law

Bejan [24,25] proposed a construction law that emphasizes that various systems in nature continuously evolve to follow the optimal path for the flow of matter and energy, thereby reducing flow resistance. Unlike Murray’s law, which focuses only on minimizing energy consumption in vascular systems, the construction law supports the design of non-Newtonian fluids and complex fluid environments, providing a broader framework [26,37].

In the field of grid layout optimization, Bejan et al. [27] used a triangular grid. Their results showed that compared with traditional square or hexagonal layouts, the triangular grid structure improved transmission performance by nearly 2× and significantly reduced average transmission time. This indicates that selecting the appropriate grid type can reduce overall transmission resistance while maintaining flow accessibility. Wang et al. [28] found that when the location of cracks is uncertain, multiscale grids (combining large pores and small branches) perform better than traditional tree-like networks. However, if the grid is too dense, a large number of pores will form within the material, potentially leading to a decline in mechanical performance. Therefore, a balance must be struck between improving fluid distribution efficiency and maintaining structural integrity; the network density must be controlled to meet flow requirements without compromising strength. This is also the key to future development.

The construction law provides ideas for the construction of vascular systems in concrete. When combined with Murray’s law, it is expected to improve energy efficiency and create favorable conditions for self-healing in materials.

### 2.2. Artificial Intelligence (AI) Assistive Technology

#### 2.2.1. Genetic Algorithm

Studies [28,29,38,39] have utilized artificial neural networks to address challenges in civil engineering, demonstrating their exceptional ability to model and solve complex problems across various fields. Artificial neural networks are commonly used to study concrete compressive and shear strengths, strain, dynamic elastic modulus, chloride permeability, and crack patterns. As shown, artificial intelligence techniques are a promising approach for predicting the self-healing efficiency of cement.

**Figure 2:**
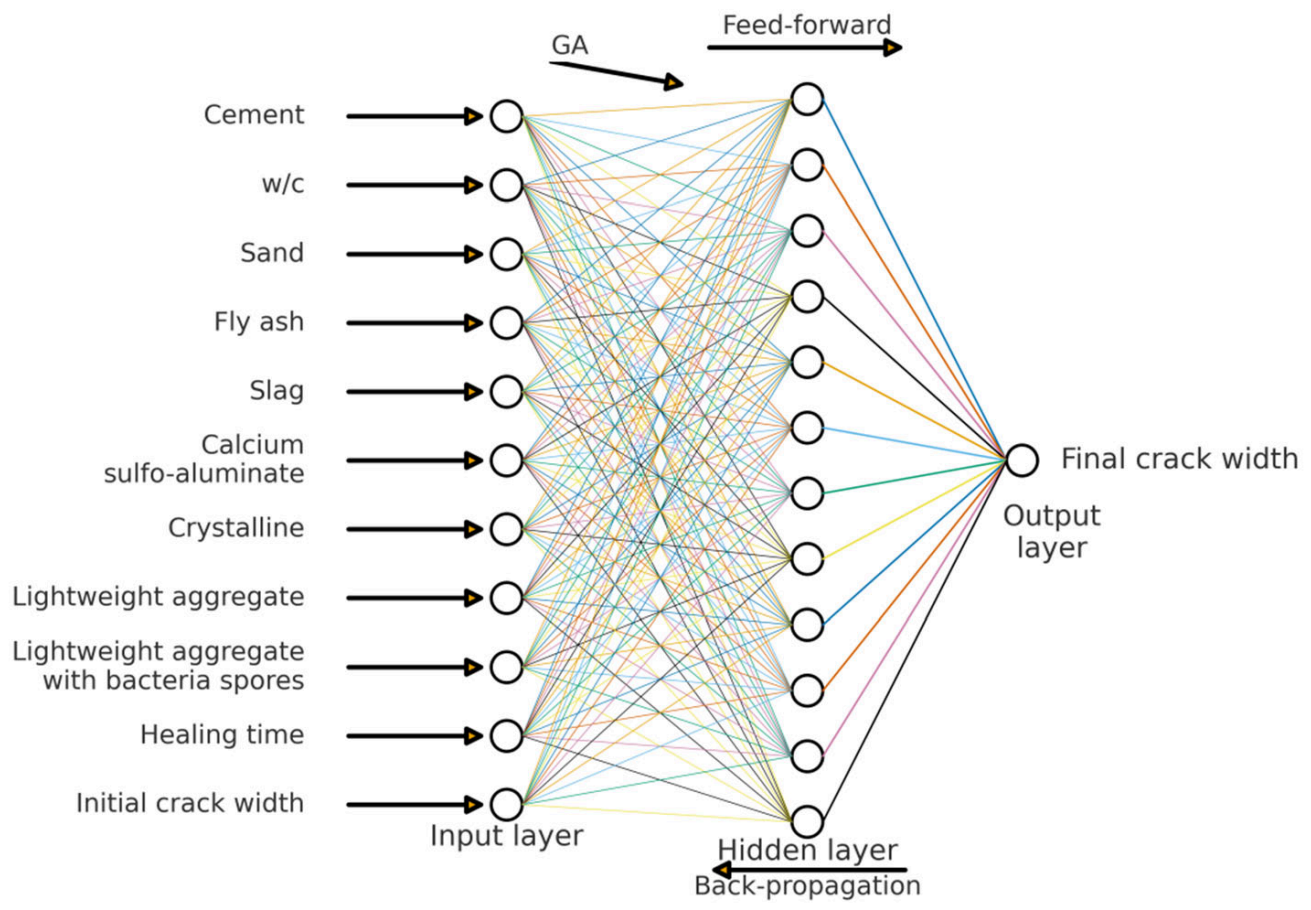
Genetic algorithm architecture-artificial neural network model (GA-ANN) for self-healing performance prediction [30].

Recently, Suleiman et al. [30] used a hybrid genetic algorithm-artificial neural network (GA-ANN) to predict the feasibility of self-healing in cement. Their model inputs included cement content, w/c ratio, types and doses of complementary cement materials, bio-repairing materials, and expansive and crystallizing additives (Figure 2). The model output was expressed as self-healing, measured by crack width. The results showed that the proposed GA-ANN model could capture the complex interactions among various self-healing agents (e.g., biochemical materials, silica-based additives, expansive and crystallizing components) and accurately predict the self-healing performance of cement materials.

Genetic algorithms possess strong global search capabilities, suitable for multi-parameter optimization problems in self-healing cement [40,41]. When combined with artificial neural networks (ANNs), genetic algorithms can be used to optimize the ANN’s network architecture, weight parameters, and hyperparameters, thereby enhancing the model’s ability to fit complex, multivariate, and nonlinear relationships, as well as its generalization potential [42,43]. However, the reliability of the GA-ANN model is highly dependent on the quality and coverage of the training data. For self-healing cement, there is significant experimental variance in specimen preparation, crack induction, curing conditions, and healing efficiency testing. Moreover, individual studies typically have limited sample sizes [44,45], making it difficult to cover various combinations of variables such as water-to-cement ratio, healing agent type, crack width, age, and environmental conditions. Consequently, even when the same experimental methods are employed, significant deviations may still exist between test specimens from different batches, leading to noise in the training data and further affecting the genetic algorithm’s evaluation of the objective function [46]. In this condition, the best parameter combination may represent only a local or apparent optimum for a specific dataset, rather than the true global optimum; at the same time, the model’s robustness will decrease when faced with new material systems or new crack conditions [47,48]. Furthermore, measurement variations from different experimental instruments, loading methods, and image processing techniques are also significant sources of training uncertainty [49]. Therefore, future applications of GA-ANN for optimizing vascular self-healing cement should not only report prediction accuracy or optimal parameters but also quantify the uncertainty of model predictions and optimization solutions by combining methods such as cross-validation, independent test sets, noise sensitivity analysis, and confidence intervals. At the same time, higher-quality, more standardized datasets should be established, and strategies such as few-shot learning, data augmentation, or surrogate model-assisted optimization should be adopted to reduce the impact of experimental bias on model training and optimization results [50,51].

#### 2.2.2. Deep Neural Network

**Figure 3:**
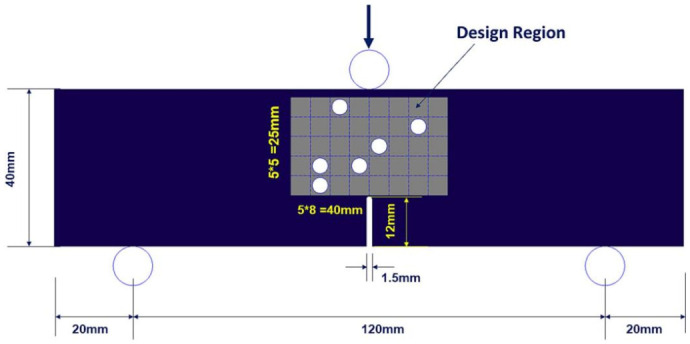
Model of a cementitious beam with vascular structures for mechanical-response prediction [31].

Wan et al. [31] effectively modeled a cement beam with six circular holes in the middle span (Figure 3), increased the number of pipe holes, and defined the target toughness based on the peak load, thereby improving overall performance. As shown in the results, the deep neural network model achieves good performance in predicting peak load and toughness, and in defining mixed objectives, with an R^2^ exceeding 0.895 on the test sets of the three ML models. However, when the objective shifts from peak load to toughness, and to a mixed objective, the DNN model’s accuracy decreases.

Deep neural networks achieve high predictive performance on large-scale datasets, support multi-objective optimization, and enable fast prediction, making them suitable for rapidly evaluating peak loads and fracture toughness of networks in different locations [52]. However, their performance is highly dependent on a large amount of high-quality training data [53]. In research on vascular self-healing cement, real experimental data are typically limited in quantity and are influenced by a combination of factors such as sample size, crack path, pipeline location, pipeline type, and loading methods; as a result, the data distribution is uneven and fails to cover the complex design space comprehensively. Although finite element simulations can provide DNNs with a large number of low-variability training samples, thereby improving model fitting accuracy, existing simulation data are typically based on simplified pore or conduit models and have not yet fully coupled Murray’s law, the construct law, and the behavior at the pipeline-cement interface [54,55]. Consequently, DNNs trained on simulation data may perform well on test sets, but their generalization capability to real cement remains uncertain. In particular, when the optimization objective shifts from peak load to fracture energy, healing efficiency, durability, or multi-objective comprehensive performance, the model may require retraining or structural adjustments [56,57]. Although transfer learning can improve the model’s adaptability to new tasks to some extent, extrapolation errors may still occur if there are significant distribution differences between the source data and the target material system [58]. Therefore, when applying DNNs to vascular self-healing cement in the future, it is essential to provide clearer specifications regarding the sources of training data, data distributions, and the model’s applicability boundaries. Additionally, methods for quantifying uncertainty—such as ensemble models, Bayesian inference, Monte Carlo perturbation, or prediction interval analysis—should be introduced to distinguish between high-confidence predictions and high-risk extrapolated predictions. The reliability of DNN optimization results for vascular structure design and practical engineering decisions can be enhanced when the limitations of the training data and the uncertainties in the predictions are clearly understood.

#### 2.2.3. Reinforcement Learning

In recent years, reinforcement learning (RL) has been particularly effective in solving complex problems [32]. This algorithm does not rely on prior knowledge or a large number of initial samples. In engineering, reinforcement learning has been applied to the design of building structures and the optimization of material proportions. In structural research, to simplify the process, the design domain is typically discretized, and an interactive simulation environment is constructed by solving control equations or applying normative constraints to meet actual requirements.

**Figure 4:**
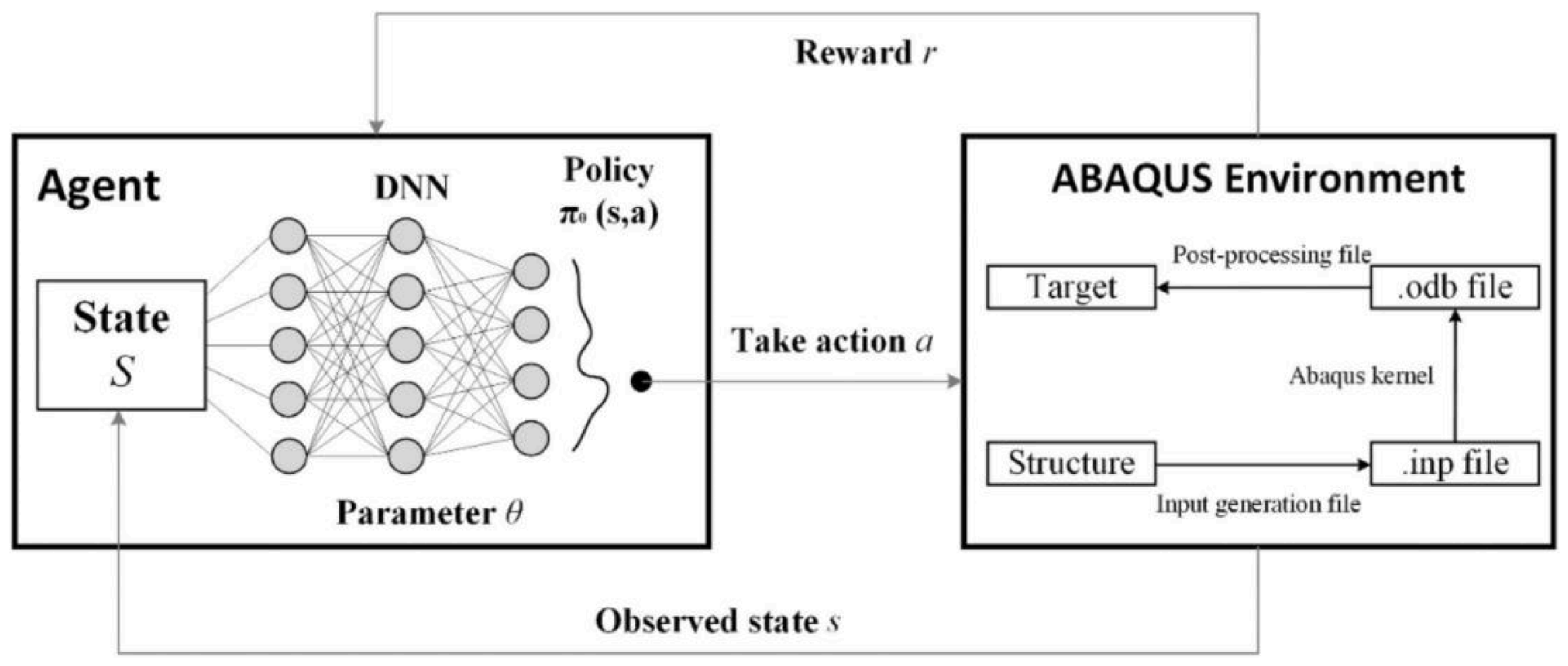
Interaction of reinforcement learning agents and Abaqus environment for vascular-layout optimization [33].

Wan et al. [33] optimized vascular layout through reinforcement learning (RL) to minimize the adverse effects of the vascular system on cement. As shown in Figure 4, this optimization was performed through interaction between RL agents and Abaqus simulation. The results show that the RL method can automatically enhance the pipe layout of self-healing concrete because the 3-hole structure with the maximum target mechanical properties (i.e., peak load or fracture energy) can be used for all independent runs, while the RL optimization method can identify the fracture energy in the new optimization task of the 4-hole concrete structure.

Reinforcement learning (RL) possesses strong adaptive capabilities and the potential to extend across multiple optimization objectives, making it suitable for addressing optimization problems in vascular self-healing cement characterized by a large design space, complex constraints, and diverse objective functions [59]. Compared to traditional supervised learning, RL can gradually identify optimal vascular layouts or pore combinations through continuous interaction between the agent and simulation software. However, the reliability of RL training is highly dependent on the realism and stability of the interactive simulation environment [60]. On the one hand, RL requires iterative trial and error across a vast number of state-action combinations; each strategy update may require finite element calculations or verification of mechanical responses, which significantly increases computational costs and training time. On the other hand, current simulation environments are rarely able to fully reflect the pipeline-matrix interface, humidity variations, aging effects, and complex service environments in real cementitious materials [61]. Therefore, the optimal vascular layout obtained by RL agents in a simulated environment may not be directly transferable to actual test specimens or engineering structures. In other words, RL optimization results are influenced not only by the design of the reward function but also by the uncertainties in simulation model assumptions, boundary conditions, and material parameters. If the reward function considers only peak load or fracture energy while ignoring healing agent delivery efficiency, long-term durability, and constructability, the resulting solution may exhibit superior mechanical performance but lack engineering applicability. Therefore, future applications of RL for vascular configuration optimization should incorporate parameter perturbations, multi-environment training, and sensitivity analysis during training to enhance policy robustness. At the same time, it is necessary to quantify uncertainty in RL outputs—for example, by comparing strategy stability under different random seeds, material parameters, and boundary conditions—and calibrate and validate simulation optimization results using experimental data. Only by reducing the gap between the simulation environment and the real material behavior can RL evolve from a “numerical optimization tool” into a reliable design method for vascular self-healing structures.

### 2.3. Coherence Between the Vascular and the Cement Matrix

Adding a vascular system to cement can enhance self-healing, heal wider cracks by continuously releasing healing agents, and perform multiple repairs [62], thereby significantly reducing resource consumption and carbon emissions associated with traditional manual maintenance. However, the presence of a hollow vascular system creates large voids, leading to a defect effect; particularly when loads are applied perpendicular to the channel direction, stress concentrations occur around the pipeline, leading to the formation of cracks, which in turn results in a decline in mechanical properties [63,64,65]. The changes in compressive and flexural strengths for different vascular fabrication methods are shown in Table 2. As shown in the table, although relevant data are lacking for some methods, the Pickering in situ printing method still indicates that hollow channels in cement can significantly affect mechanical properties without polymers or other pipeline materials, especially when the pipeline diameter exceeds 1 mm. For structures incorporating pipeline materials, the mechanical properties of the pipeline materials themselves and their adhesion to the cement also affect the composite strength of the structure. For example, the flexural strength increases when reinforced with PLA and steel reinforcement, but decreases when the pipeline materials are PVA and ABS. This demonstrates that only when the stiffness, adhesion, and geometric structure of the polymer channels are properly controlled can some of these adverse effects be effectively mitigated.

To ensure the mechanical properties of self-healing cement while maintaining good healing ability, scholars have adopted various methods, including the addition of steel bars, fibers, and shape memory alloys, to enhance the bending resistance of cement [66], controlling the vascular diameter during additive manufacturing to minimize mechanical property loss, and toughening printable cement using a curing agent with high tensile strength after curing.

**Figure 5:**
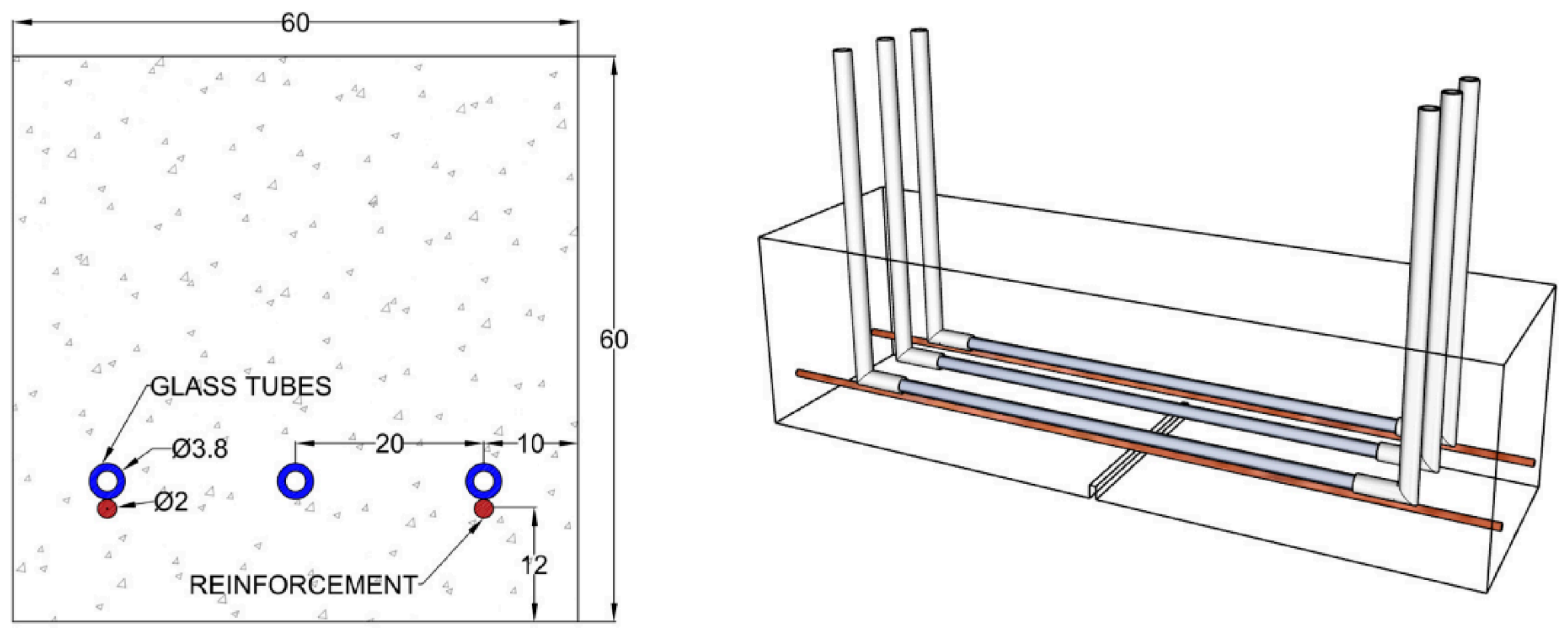
Illustration of reinforcement-assisted vascular self-healing [69].

Directly enhancing the bending performance of cement with added materials can control crack width to some extent (Figure 5) and reduce the healing work of the vascular system, thereby lowering the requirements on the vascular system and alleviating design demands [62,67,68]. Reinforced concrete is the primary building material, and this method makes vascular systems feasible for practical engineering. However, the placement of these steel bars requires space within the concrete, which also limits the distribution of the three-dimensional vascular network. Therefore, additives must be avoided during the manufacturing of 3D vascular systems.

A vascular system composed of external materials can achieve greater bending strength due to its material characteristics, compensating for the loss of mechanical properties caused by the presence of the vascular system [22,62,69]. However, compared with the in situ printing strategy that relies on fugitive ink, prefabricated vascular systems produced by additive manufacturing often entail higher costs and more processing steps [70,71]. Moreover, printing defects may cause voids [72], leading to cement leakage and thus affecting the repair performance. Although the printing strategy for fugitive ink retains the flexibility of freeform construction characteristic of additive manufacturing [73,74], stress concentration within the hollow vascular structure still exists, resulting in a greater decline in mechanical properties. Zhang et al. [64] employed the carbonation strengthening method to densify the vascular wall and inhibit crack propagation, thereby enhancing compressive strength. Still, they yielded only a modest improvement in bending strength. This is because the carbonation-strengthening method involves a gradient weakening [75]. With increasing depth, the degree of strengthening decreases, leading to densification of the vascular wall that can inhibit crack formation. Still, it is challenging to effectively address crack development in bending failure. Therefore, in the future, we should aim to strengthen the cement matrix as a whole and explore the overall impact of the improved matrix on the healing agent’s flow state and the curing reaction. Indeed, the enhanced cement matrix lacks a vascular system and performs better than ordinary cement specimens, but we are pursuing a lower strength loss under stress concentration. Therefore, a comprehensive strengthening method for the cement matrix should be tested in the future, and the extensive impact of the improved matrix on the flow state of the healing agent and the curing reaction should also be explored. Admittedly, the enhanced cement matrix performs better than standard cement specimens, even without a vascular network. However, the objective here is to achieve a lower rate of strength loss under stress concentration. From the perspective of polymer composites, mechanical compensation is distinct from stress-concentration mitigation, as fiber reinforcement, polymer modification, and matrix densification primarily enhance damage tolerance or retard crack propagation.

In recent years, additive manufacturing has played an essential role in the manufacture of vascular systems. Relying on its free-form printing capabilities, this method enables flexible vascular designs that support a range of dimensions and shapes. It combines Murray’s law, construction laws, and fluid mechanics equations to refine vascular design [22,23] and investigate the appropriate pipe volume fraction and configuration. However, cement has a non-homogeneous surface, unlike the conditions assumed in Murray’s law for biological blood vessels. Hence, obstacles remain in terms of refinement, and there is also considerable room for improvement in the development of models that combine fluid mechanics equations.

**Figure 6:**
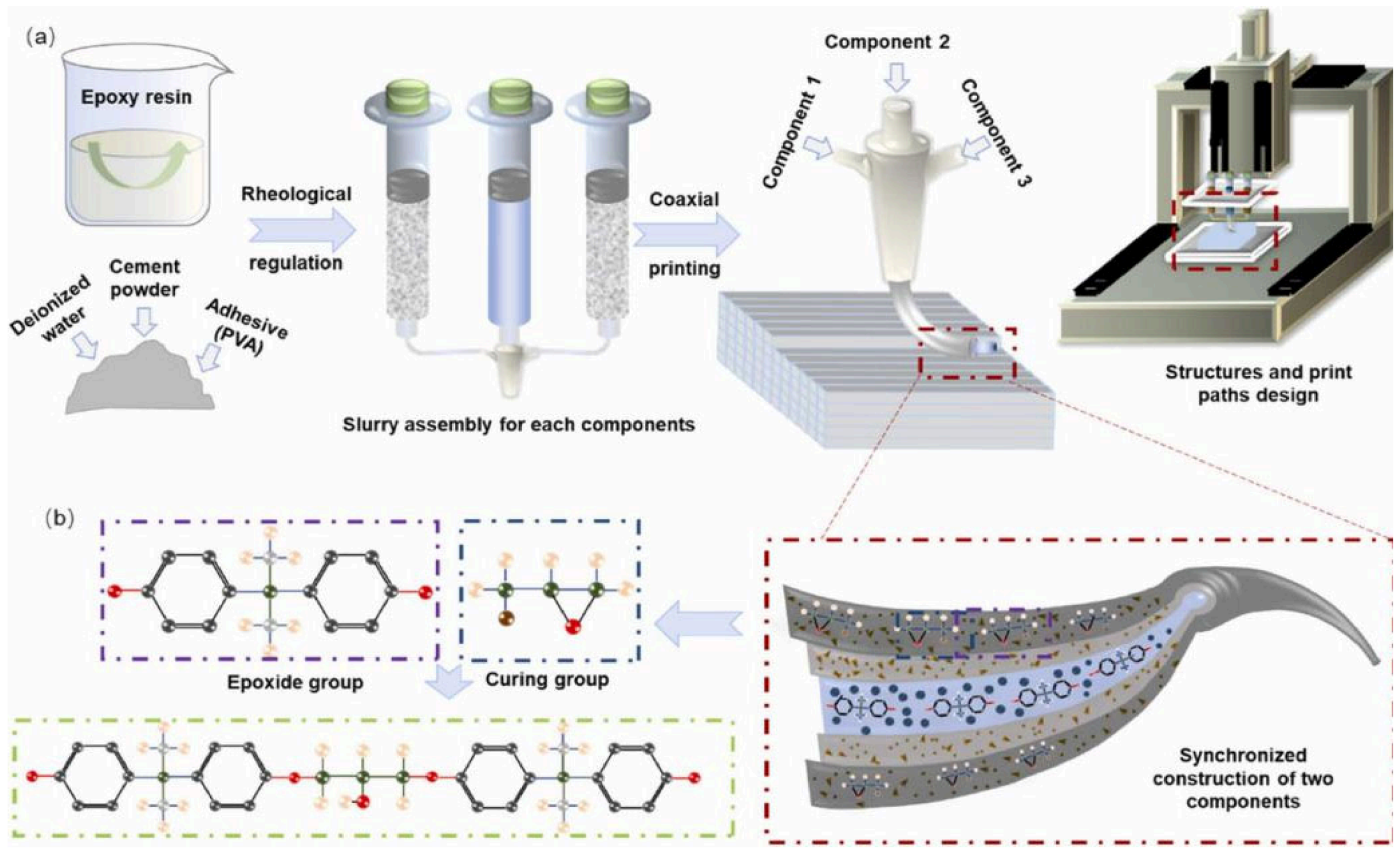
Bionic coaxial self-healing printing mechanism: (**a**) three-channel coaxial printing procedure of multilayered microstructure; (**b**) self-healing reaction between epoxy resin components [77].

Another method is integrated molding, specifically by altering the rheological properties of cement to impart shear-thinning behavior, which can then be used directly for printing [76,77]. During printing, space is reserved for the vascular, thereby simplifying the production of self-healing cement and facilitating its implementation. Given the precedents in 3D-printed cement, it has the potential for use in real-world engineering applications. However, due to the physical properties of cement and the heat of hydration generated during curing, the viscosity may change, leading to poor vascular morphology. There is still much room for improvement through modification. In addition, recent research has combined healing agents with silicate matrices via coaxial printing, as shown in Figure 6. Once a crack forms, the healing agent flows into the silicate matrix layer, thereby enabling rapid healing [77]. After curing, the curing agent interacts closely with the silicate matrix to form an interpenetrating network, thereby improving the mechanical properties of the structure.

### 2.4. Critical Discussion of Vascular Design and AI-Assisted Optimization

Current design methods for vascular systems generally address different theoretical problems and are often developed around a single performance objective. At the network-design level, Murray’s law is used to optimize vascular branching by minimizing flow-energy losses within branched channels [22]. Constructal theory provides a broader framework for optimizing transport paths in complex flow networks [27]. However, these approaches mainly focus on the transport of healing agents. The influence of the vascular network itself on the mechanical properties of cementitious materials is often treated as a secondary issue [63,64]. In the field of artificial intelligence, GA–ANN is a data-driven method for parameter prediction and optimization. It can be used to predict healing efficiency, but its reliability is strongly dependent on data quality [30]. DNN is more suitable for identifying nonlinear relationships between the number, position, and arrangement of channels and mechanical responses such as peak load, fracture energy, and toughness [31]. Once trained, a DNN can replace a large number of repeated finite element analyses. The mechanical response of different vascular layouts can therefore be predicted rapidly, improving the efficiency of design screening [31]. In contrast, RL interacts repeatedly with a finite element simulation environment. The number and position of channels are adjusted iteratively. More favorable layouts are then selected according to indicators such as peak load or fracture energy [33]. RL is therefore more suitable for optimization problems involving a large number of candidate layouts and sequential design decisions. Nevertheless, current AI-based approaches mainly focus on channel positioning or healing-efficiency prediction. The vascular networks are usually represented by simple geometries. The effects of network shape, branching pattern, and hierarchical architecture are rarely considered. These methods are complementary to some extent, but their optimization objectives, applicability ranges, and underlying assumptions differ. A design that improves healing-agent transport may not simultaneously minimize mechanical degradation or ensure interfacial durability and manufacturing feasibility [22,63,76].

The apparently contradictory results reported for vascular-network design mainly reflect trade-offs among different performance objectives. Branched three-dimensional vascular networks can increase the probability of crack–channel intersection and improve healing-agent transport. However, the vascular volume fraction and interfacial area are also increased. As a result, channel-induced mechanical degradation may become more severe [64]. Smaller channels can reduce the disturbance to the effective load-bearing section and the local stress field. However, flow resistance may increase, and the risk of blockage may become higher [72,76]. Regular networks are more convenient for mathematical description and numerical optimization. In real cementitious materials, however, crack paths are random. They are jointly affected by the loading mode, material heterogeneity, and boundary conditions [34,35,36]. Therefore, no universally optimal channel diameter or branching pattern can be defined independently of a specific application. A rational design should account for the statistical characteristics of crack development, the viscosity and other rheological properties of the healing agent, the vascular volume fraction, and the loading condition. Vascular-network design should therefore be shifted from single-objective optimization to multi-objective coordinated design. Future layouts should simultaneously consider transport efficiency, crack–channel intersection probability, channel-induced mechanical degradation, polymer–cement interfacial stability, manufacturability, and repeated delivery capacity [23,63,76,77]. Artificial intelligence can improve design efficiency, but physical constraints, material mechanisms, and experimental validation cannot be replaced. Objectives such as peak load, fracture energy, toughness, crack coverage, and healing efficiency may compete with each other. Future models should therefore couple vascular geometry, healing-agent transport, crack propagation, and healing reactions. Channel position or healing efficiency should not be treated as the only optimization variable.

The main limitation of current research is the restricted data basis. Most design data are obtained from finite element simulations or small-scale specimens. Cementitious materials are commonly simplified as homogeneous media. Crack locations and propagation paths are often idealized or intentionally controlled to produce predetermined fracture paths [34]. Polymer–cement compatibility, interfacial debonding, and long-term interfacial evolution have not been adequately characterized. Environmental exposure, material aging, and repeated damage–healing cycles are also rarely incorporated into design models. In addition, most studies primarily report the optimal prediction results. Prediction intervals and independent experimental validation are seldom provided. Therefore, current vascular-design theories and AI-assisted optimization methods are more appropriately regarded as tools for candidate screening and parameter-sensitivity analysis. They should not yet be considered sufficient evidence of engineering feasibility or long-term service reliability.

## 3. Manufacture of Polymeric, Hybrid, and Cementitious Vascular Systems

The development of vascular self-healing technology is driven by two primary factors: the gradual refinement of vascular design principles and ongoing advancements in manufacturing technologies. Early hollow glass fiber tube technology was inspired by the traditional method of embedding healing agents within concrete. This involved filling glass fiber tubes with healing agents, embedding them in cement [18,68,69,78], and then pulling out steel rods [62,79], which led to the idea of implanting hollow vascular systems. From this, three types of additive manufacturing-based vascular manufacturing technologies were developed.

Previous classifications of manufacturing methods included implant methods, removable materials, and additive manufacturing. However, research on removable materials primarily utilizes thermoplastic organic films and PVA. Thermoplastic organic films have thermoplastic properties that help seal and preserve the healing agent, making them more akin to early technology. PVA, on the other hand, requires additive manufacturing to achieve a customized tubing design, with additive manufacturing playing a more significant role throughout the process from design to formation. Therefore, removable materials have not evolved from early technologies, nor have they matured into integrated technologies similar to additive manufacturing. In addition, considering the latest literature, the previous review of additive manufacturing in vascular manufacturing was not comprehensive and overlooked research on printable cement. Therefore, this paper reviews the evolution of early manufacturing technologies and comprehensively illustrates recent developments in pipeline manufacturing using additive manufacturing technologies, comparing different methods in terms of manufacturing complexity, cost, scalability, impact on mechanical properties, healing efficiency, and potential for practical applications. The relevant metrics are summarized in Table 3 and Table 4.

### 3.1. Early Manufacturing Methods

**Figure 7:**
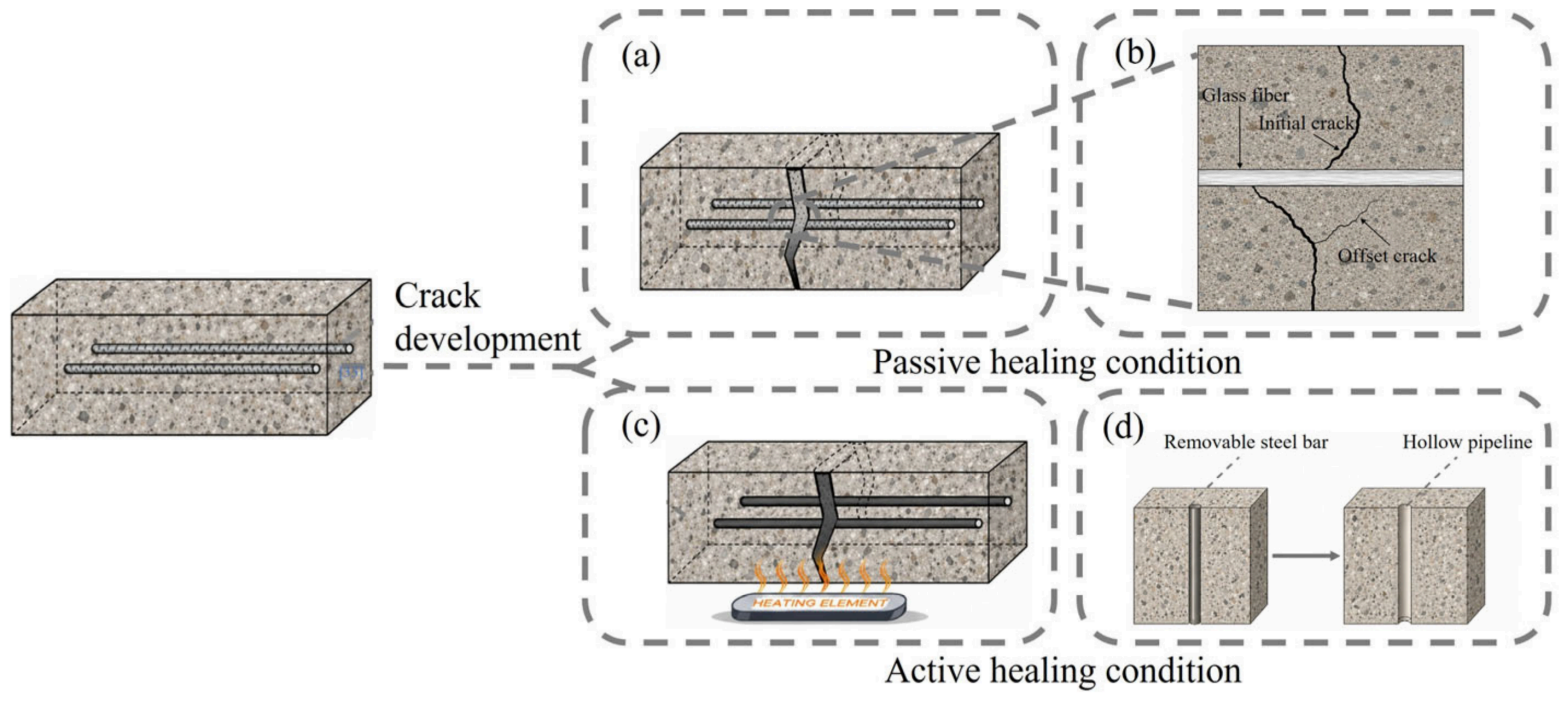
Self-healing mechanisms of vascularized cement in active and passive conditions: (**a**) Pre-hidden fiberglass tubes. (**b**) Crack development paths. (**c**) Heating melts, releasing the healing agent. (**d**) A pipeline for pumping the healing agent.

The primary methods for early-stage vascular manufacturing can be categorized into three types based on their application methods (encapsulated fiberglass, organic film pipes, and removable materials). As shown in Figure 7, these methods can be further classified into active and passive mechanisms. Passive healing relies on crack penetration to release the healing agent, while active methods rely on melting to release it or on exposing the pipe to pump it in.

#### 3.1.1. Encapsulated Fiberglass

In the early stages of vascular manufacturing, glass fiber tubes were mainly used as the primary vehicle for healing agents. At that time, hollow vascular systems for transporting healing agents had not yet been invented, and various materials or technologies had to be used alongside embedded fiber tubes to improve the effectiveness of cement repairs. Dry et al. [18] began exploring vascular self-healing by using glass fiber tubes for concrete self-healing applications. They developed an intelligent timing system to release the healing agent from the fibers into the cement matrix, thereby repairing matrix cracks. In passive mode (Figure 7a), mechanical stress from cement cracking causes the embedded hollow glass fiber tubes to rupture. After the fiber tubes rupture, the healing agent stored inside flows into the cracks by capillary action, solidifies upon contact with air, and fills them. Secondary loading results show that the bending strength of the repaired concrete beams was significantly restored. In active mode (Figure 7c), the stored healing agent can be released by melting the wax coating under external heating.

Based on research by Dry and Li et al. [78], a passive self-healing cement-based composite material was developed. By leveraging the strain-hardening properties of engineered cement-based composites, cracks can be generated within a controllable range, optimizing the use of healing agents by reducing the demand for repair agents and effectively driving capillary forces. After secondary loading repair, the elastic modulus returns to its original value, indicating that the mechanical properties have been restored. Subsequently, Kuang and Ou [68,69] proposed a new method for controlling cracks that absorbs energy via SMA’s hyperplastic effect during loading and releases it after unloading, thereby driving the cement to close. After SMA converts large cracks into small cracks, the healing agent enhances local bonding, and the two complement each other.

Early vascular self-healing technologies have demonstrated preliminary feasibility for application. Still, their technical limitations are equally significant: hollow glass fiber tube carriers are only suitable for small prefabricated components or laboratory research settings. In large-scale engineering applications, issues such as poor controllability of glass fiber tube spatial distribution and pronounced brittleness of the tubes themselves are particularly evident [6]. From the perspective of mechanical compatibility, there is a dilemma in regulating the brittleness and toughness of glass fiber tubes: if the tube walls are excessively brittle, they are prone to fracture during the early stages of mixing and forming the cement-based material, completely losing their subsequent repair function; if the tube walls are excessively tough, they cannot fracture concurrently with cracks in the matrix, leading to premature leakage and failure of the healing agent. Balancing brittleness and toughness remains a core bottleneck in this technology that urgently requires breakthroughs; when applied to large-scale components, glass fiber tubes also face challenges such as limited healing agent capacity in individual tubes and difficulties in achieving uniform spatial distribution. Excessive dosage not only significantly increases material costs but may also impair the intrinsic mechanical properties of the cement matrix. By incorporating engineering cementitious composite (ECC) and shape memory alloy (SMA) technologies into the glass fiber tube self-healing system, the scope of application for vascular-type self-healing systems has been effectively expanded. Through precise control of the matrix crack width, this composite structure can be applied to scenarios—such as bridges and seismic-resistant components—that demand high material toughness and deformation capacity, thereby achieving more efficient crack repair [80,81]; however, this composite solution still has numerous shortcomings: SMA materials are inherently expensive [82]; their processing, forming, and on-site installation are complex; and their interfacial adhesion to the cement matrix is poor [83]. Meanwhile, the PSS-ECC system is limited by the insufficient total volume of the repair agent, resulting in significant application limitations when addressing multiple random cracks. At the same time, the initiation and propagation of cracks in the cement matrix are inherently random; some cracks (as shown in Figure 7b) cannot penetrate the wall of the glass fiber tube, preventing the repair agent from being triggered and released. Given that the fiber tube carrier is made of inorganic glass, performance evaluations of the polymer components in this technical approach should focus on the stored adhesive repair agent and the interface coating, rather than the structural properties of the glass tube wall itself. Although the manufacturing process is relatively simple—prefabricated glass tubes can be directly embedded into the matrix during molding—the system’s scalability and engineering applicability are limited. In large-scale components, it is difficult to precisely control the spatial positioning of the tubes, the probability of fracture initiation, and the alignment between cracks and tube walls. Furthermore, there are currently few reports on direct bond strength data for the glass-cement matrix interface or the repair agent-cement matrix interface. Therefore, the stiffness recovery values reported in the existing literature must be interpreted comprehensively in light of the elastic modulus contribution of ECC, the surface modulus effect of SMA, and the adhesive action of the repair agent itself; they cannot be simply attributed to the inherent properties of the glass fiber tube carrier.

#### 3.1.2. Thermoplastic Organic Film Pipe

**Figure 8:**
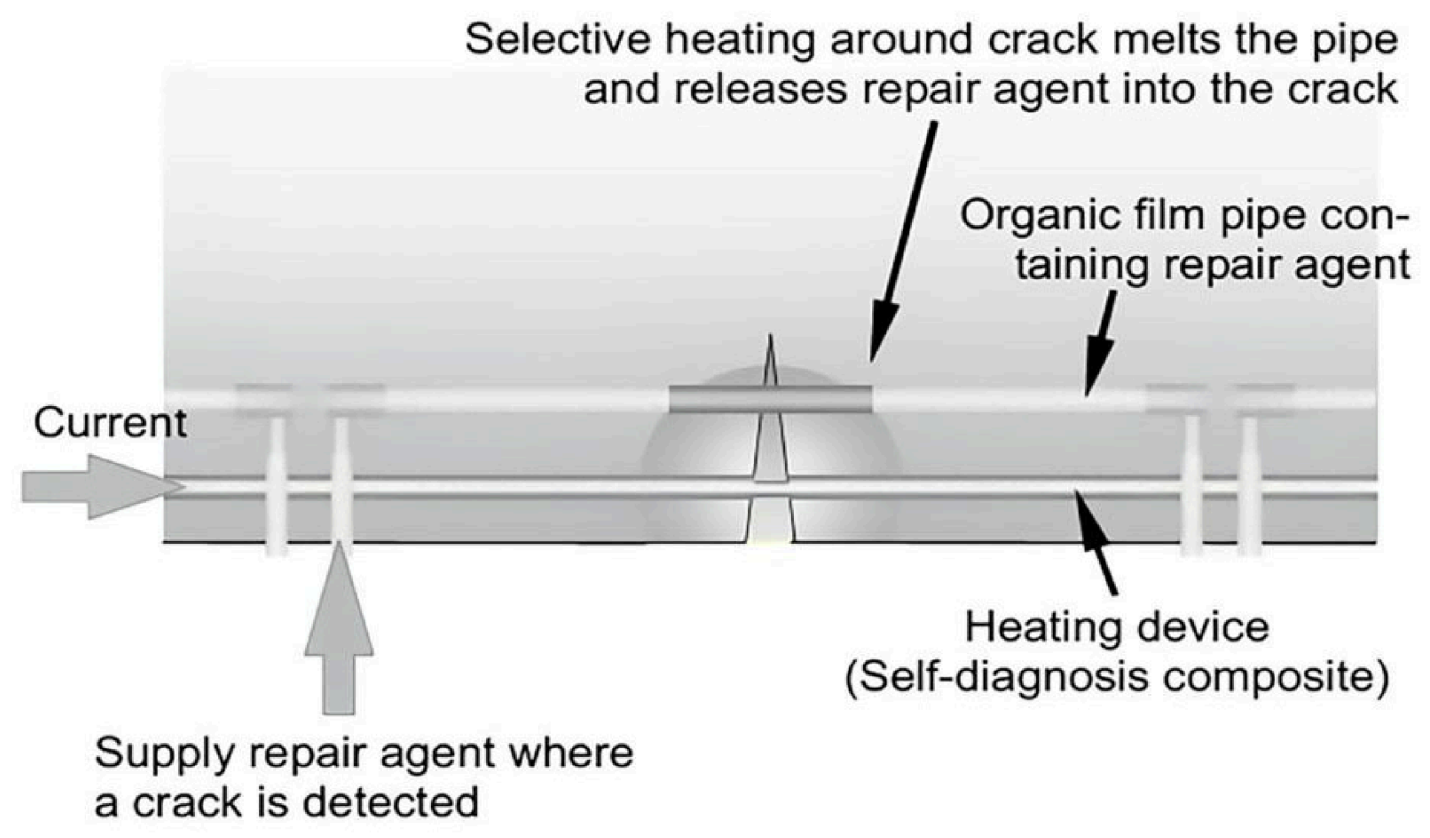
Self-diagnostic healing diagram using thermoplastic organic film pipes [84].

Nishiwaki et al. [84] developed a self-healing cement composed of a self-diagnostic, cement-based composite material combined with organic thin-film tubes. The composite material consists of cement, a conductive matrix, and ceramic fibers, as shown in Figure 8. Cracks alter the conductive matrix’s resistance, and selective heating in the area where the resistance changes melts the thermoplastic tubes, releasing the healing agent.

Self-diagnostic healing methods are highly controllable [85], and selective heating can eliminate resource waste in undamaged areas. Heating systems require an external power supply [86], increasing costs and resource consumption beyond those of the cement itself. They are not suitable for environments without power supplies, and high temperatures can degrade cement performance [87]. Composite systems are challenging to manufacture and difficult to implement under engineering conditions [88,89]. In terms of self-healing performance, there is no cycle replenishment mechanism [10], and the amount of the healing agent is insufficient to support multiple healing releases.

Future research can investigate the relationship between crack width and heat-generation resistance, or use more advanced detection technologies to replace resistance sensing, thereby altering the heat-release transport mechanism, optimizing energy supply requirements, and preventing excessive temperatures that affect cement performance. IT can also develop a repeatable self-healing vascular system that enables multiple repairs. Thermoplastic films were selected because they can store the curing agent during casting and release it through melting or softening under specific heating conditions. Compared to passive glass tubes, this material offers more precise control during activation; however, the need for a conductive matrix, ceramic fibers, a power supply, and a localized temperature control system results in a complex and costly manufacturing process, thereby limiting its potential for large-scale production. In addition, the polymer film must undergo testing for the following properties: heat deflection behavior, alkali stability before activation, UV/thermal aging behavior during storage or use, and recyclability, as these parameters have not been quantitatively evaluated in existing cement research.

#### 3.1.3. Linear Vascular

The steel bar extraction method marks the emergence of a new vascular manufacturing solution. After the steel bars are removed, a hollow vascular system remains in place to transport the healing agent. Dry and McMillan [79] developed a steel bar extraction method, as shown in Figure 7d, in which steel bars are embedded in the cement, cured for 24 h, and then extracted, leaving a passage inside the cement for transporting the healing agent. When cracks occur, they intersect with the hollow vascular system. At the same time, the healing agent is pumped into the vascular system and sucked into the crack area by negative pressure, gravity, or capillary action, and then solidifies and repairs the crack. This method simplifies the manufacturing process, avoids complex packaging, and is suitable for producing prefabricated components. However, the issue of reduced mechanical properties in vascularized cement still needs to be addressed. Based on this, Pareek [62] combined superelastic alloy steel (SEA) to reduce the healing pressure of large cracks in the vascular system and improve self-healing performance. Robert et al. [90] used heat-shrinkable or polyurethane tubes to form a pipe system that could be cleaned with hot water after solidification, leaving no residual material. The emergence of heat-shrinkable tubes has optimized the method of linear pipes. Removing the heated, shrunk mold prevents structural damage and improves the integrity of the cement.

The linear vascular method offers a straightforward self-healing implementation that avoids fragile fiber pipe breakage and can be healed by repeatedly injecting healing agents via an external pumping system. Its feasibility has been verified in full-scale bridges [91], providing new ideas for future scholars. However, the 1D linear design sacrifices vascular flexibility and creates new space for the introduction of corrosive media [92]. Future research should focus on enhancing scalability, with 3D printing as a promising direction [70]. This advanced technique was indeed further explored by later scholars, as described in detail below. Another area of research involves methods to control solidification time, thereby improving the fault tolerance of steel rod extraction and preventing structural damage [93]. For polymer-related evaluations, this method primarily uses removable heat-shrink tubing or polyurethane (PU) tubing, along with polymer curing agents, rather than embedded polymer vascular walls. Heat-shrink tubing or polyurethane tubing is chosen because of its removability and hot-water washability, which make this approach relatively low-cost and more suitable for easily processed prefabricated components; however, there is currently a lack of clear quantitative data regarding issues such as cleaning efficiency, port sealing, polymer residue, and strength loss with or without a luminal structure.

### 3.2. Additive Manufacturing-Based Fabrication Methods

In recent years, additive manufacturing has become the primary method of vascular manufacturing. It can be further subdivided by application method (polymer printing, Pickering emulsion, and printable cement). These three methods are classified because, although they are based on additive manufacturing, their extruded materials, methods of use, and development ideas differ significantly. These three methods involve, respectively, using 3D printing to generate vascular models that comply with design requirements, transplanting them into cement, utilizing an interface-enhanced polymerization method to produce a Pickering emulsion, and then printing it in the cement matrix as a fugitive ink and using cement as the extruded material for direct printing. These additive manufacturing techniques are closely related to polymer science and include technologies such as polymer printing, sacrificial polymer templates, colloidal or emulsion inks, and hybrid polymer-inorganic systems.

#### 3.2.1. Additive Manufacturing of Polymeric Vascular Networks

Linear vascular networks have design limitations. With advances in design principles, it is now possible to realize vascular designs in accordance with Murray’s law and construction laws and explore additional configurations for vascular self-healing. Scholars have developed a new manufacturing method that incorporates additive manufacturing technology [70]. Currently, there are two solutions for additive manufacturing polymers. One is non-sacrificial materials, such as acrylonitrile butadiene styrene ABS [94] and polylactic acid PLA [22], and the other is water-soluble sacrificial materials mainly composed of PVA [95], both of which avoid the effects of heating and mechanical removal on the integrity of the cement structure. The selection of ABS, PLA, PVA, or other printable polymers should consider shape retention, water sensitivity, alkaline stability, and interfacial bonding with hydration products.

**Figure 9:**
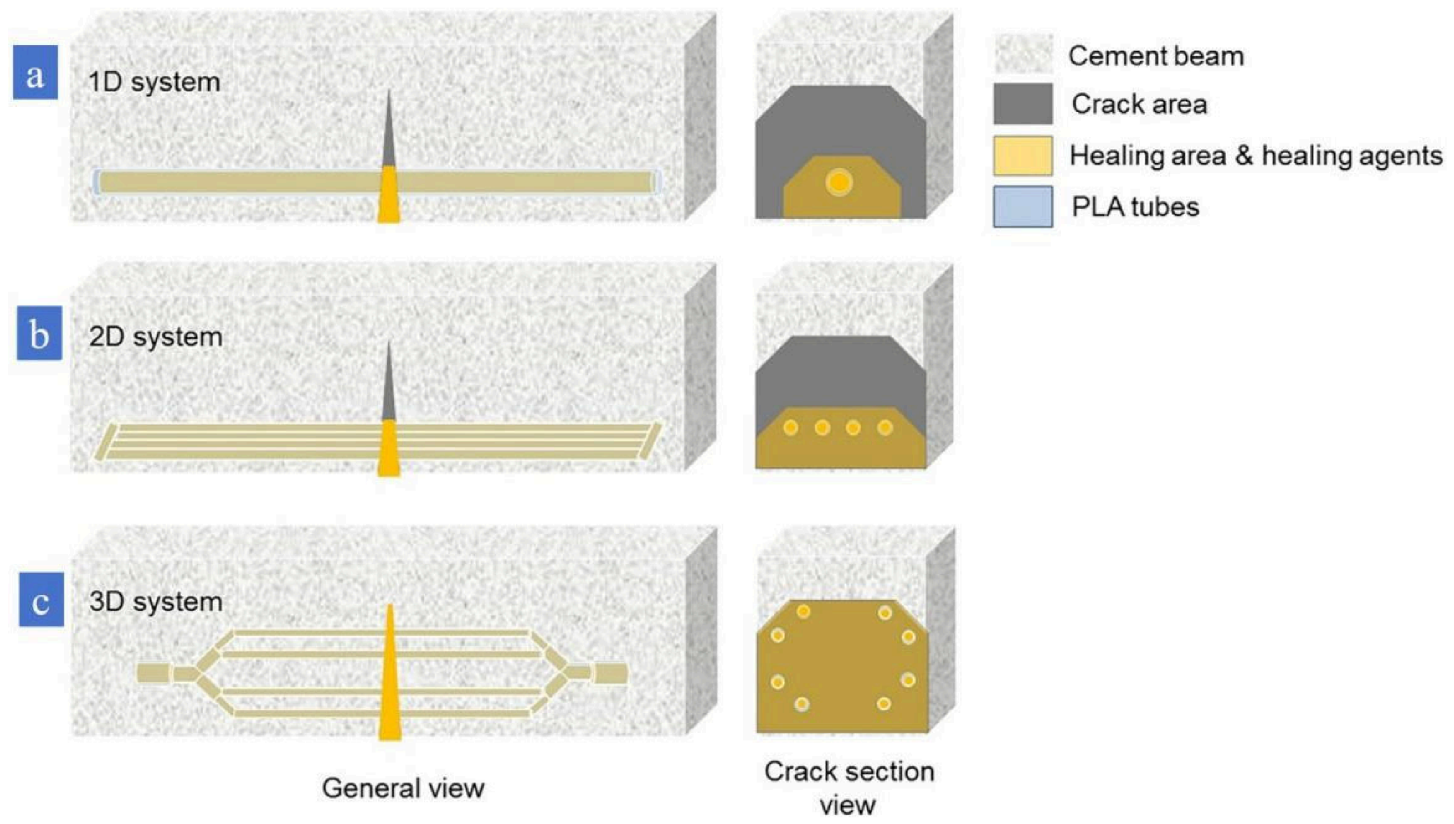
(**a**) 1D, (**b**) 2D, and (**c**) 3D self-healing solutions [22].

Regarding non-sacrificial materials, Li et al. [22] used PLA to design and fabricate a biomimetic three-dimensional (3D) tubular system in accordance with Murray’s law (Figure 9). They also designed one-dimensional (1D) and two-dimensional (2D) systems for comparison with the 3D vascular system. Mechanical performance tests confirmed that all tubular systems could deliver healing agents after crack formation, and the 3D system demonstrated superior healing performance. Wan et al. [63] used acrylonitrile butadiene styrene (ABS) filaments to print four sets of vascular networks (two different printing directions and two different printing layer heights) and studied the effect of printing parameters on the mechanical properties and watertightness of vascular self-healing cement. De Nardi et al. [96] used 3D-printed tetrahedral miniature vascular networks (MVNs) to store and deliver healing agents to damaged areas within a cement matrix. They analyzed the optimal size of the MVNs (to maximize the volume of the healing agent), the optimal surface profile (to achieve enhanced bond stress with the matrix), their optimal distribution in the mixture, and the optimal mechanical properties to ensure survival during the cement production stage and the fragility of crack formation. De Nardi et al. [97] further developed the TET structure with coaxial hollow tendons (d-TET) to store two-component healing agents, thereby addressing the short shelf life associated with single-component healing agents. From a long-term perspective, there are significant differences between PLA and ABS materials. PLA is easy to print and can be partially derived from renewable materials, but its ester bond structure is prone to hydrolysis; environments with high alkalinity, humidity, and high temperatures accelerate molecular weight loss, material embrittlement, and changes in crack initiation and fracture behavior; meanwhile, exposure to ultraviolet (UV) light during filament storage or before injection molding can degrade the performance of prints. ABS avoids ester bond hydrolysis and is generally less sensitive to moisture during injection molding; however, its butadiene-rich phase structure is susceptible to UV and thermal oxidative aging, which may lead to increased material brittleness and changes in interlayer fracture behavior. As the embedded network structure is largely unexposed to direct sunlight, its primary service risks are more likely to manifest as pipeline diameter contraction due to creep, thermal expansion mismatch, interlayer leakage, and polymer-binder desorption during wet-dry and thermal cycling. The bio-based nature of PLA does not imply that it will biodegrade in a concrete service environment, and neither PLA nor ABS can be practically recycled after permanent embedding. Therefore, material selection should be based on channel continuity, load-bearing capacity under pressure and flow, and trigger reliability after composite treatment involving alkali, moisture, and heat, rather than relying solely on initial printability or nominal recyclability.

**Figure 10:**
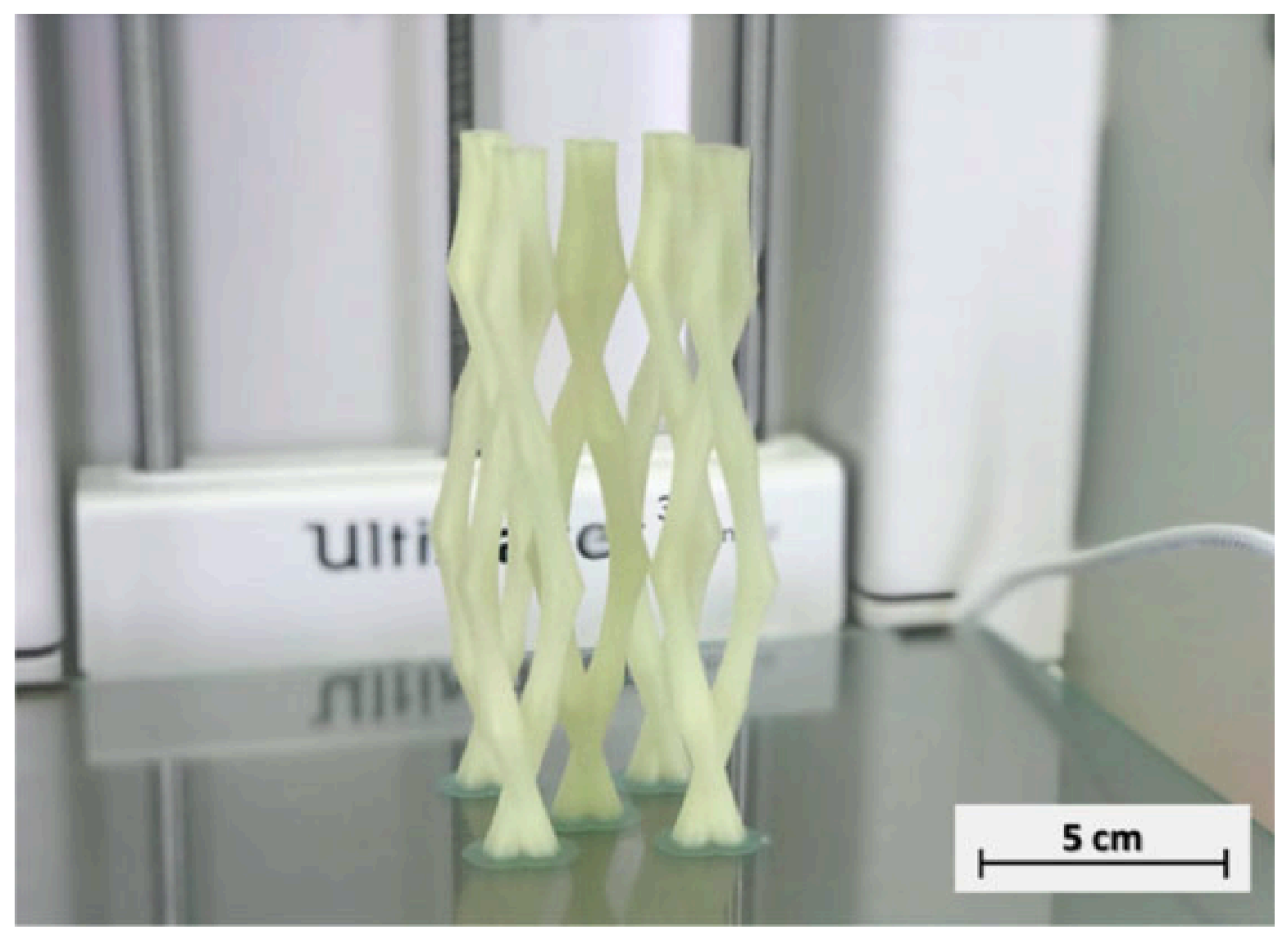
Twisted-pair channels for a PVA sacrificial vascular system [98].

**Figure 11:**
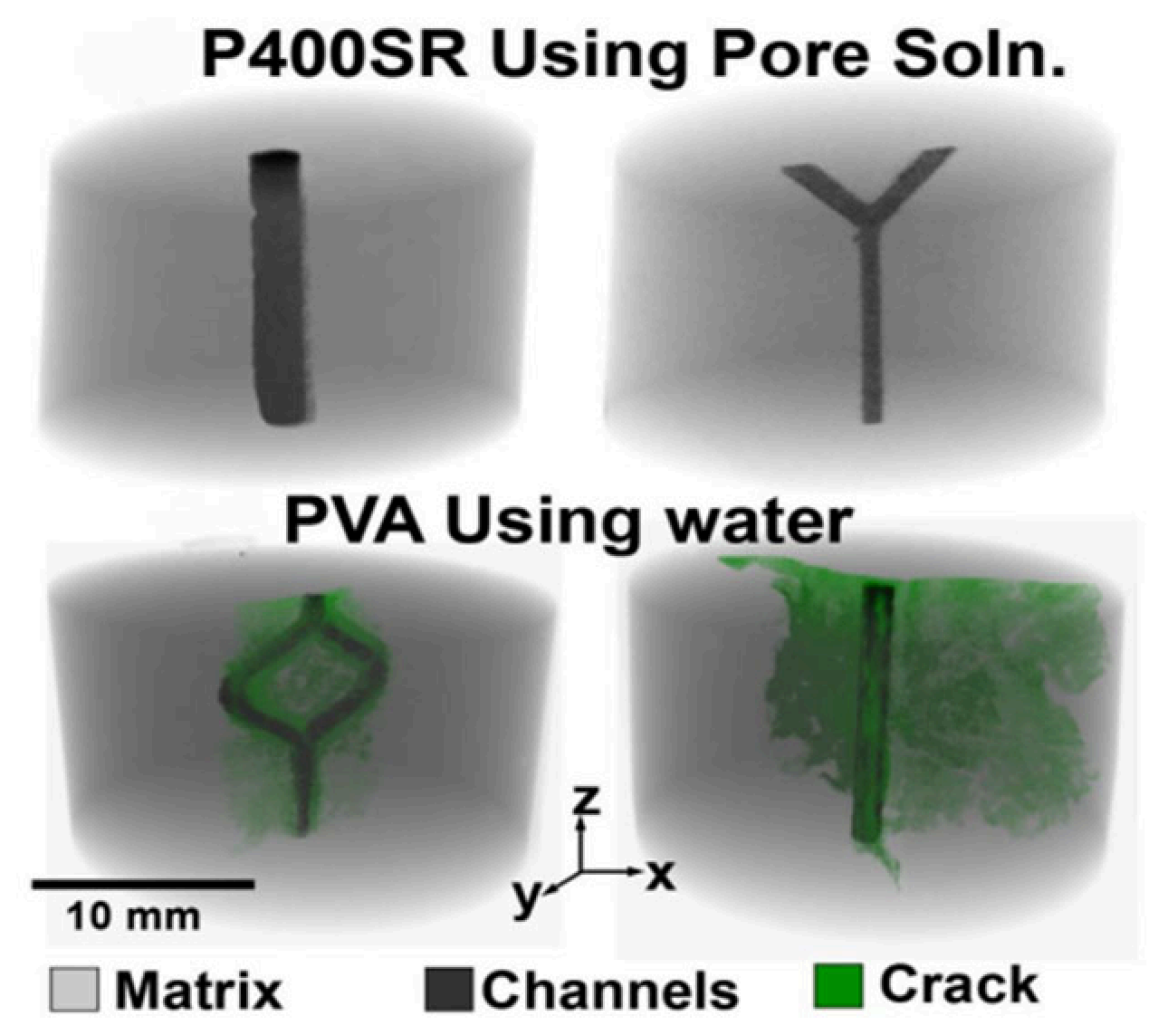
Three-dimensional constructed images of P400SR and PVA sacrificial vascular materials [99].

In terms of sacrificial materials, Li et al. [98] developed a new method for 3D printing complex internal hollow vascular structures using polyvinyl alcohol (PVA), with twin-twisted channels to deliver a two-component healing agent (Figure 10). However, when the water-cement ratio exceeds a critical value, PVA absorbs water and expands, leading to early cracking during cement solidification and compromising its structural integrity. Based on this, Wan et al. [23] coated the PVA surface with wax to prevent contact with cement, thereby preventing PVA from absorbing moisture during cement solidification, limiting its expansion, and avoiding the formation of early cracks. Osan et al. [99] conducted experiments using P400SR and PVA (Figure 11). For example, 3D construction images showed that P400SR does not cause expansion or cracking in the matrix, making it a promising material candidate. For a sacrificial template, the desired long-term outcome is complete removal rather than retention of polymer strength. PVA must remain dimensionally stable during early hydration but subsequently dissolve without leaving polymer-rich or wax-rich residues; dissolution time, residual organic mass, channel-wall wettability, and post-removal hydraulic resistance should therefore be quantified. P400SR may improve dimensional stability, but its removal chemistry, residue, and recovery or disposal route remain insufficiently documented. From a sustainability perspective, sacrificial templates can avoid a permanent polymer-cement interface. Still, dissolution water, polymer-containing effluent, coating materials, and channel-cleaning energy should be included in life-cycle assessments.

The above solution demonstrates the significance of additive manufacturing polymers in the design of complex biomimetic structures. It overcomes the limitations of traditional one- and two-dimensional designs, stimulates continuous research and development in vascular design, expands the range of healing agents, and improves healing efficiency and water resistance. However, it is still necessary to reduce the volume fraction and pore size to minimize the loss of mechanical properties in the embedded hollow vascular structures, while enhancing interface adhesion with the cement matrix. In terms of scale, the printer size limits the use of standard test pieces [70]. In the future, printer use can be optimized, or the physical properties of the printing material can be improved with modified additives to avoid gaps during layer-by-layer printing that cause cement leakage and pipe blockages, affecting the self-healing process. Combining monitoring technology with self-healing is also a direction for development [100], enabling real-time crack detection and selective healing. It is also necessary to study how to improve the mechanical properties compromised by the presence of the vascular system using combined template materials. Similar to steel and fiber reinforcement, which can enhance concrete’s tensile and bending strength, this is also a significant research direction.

#### 3.2.2. In Situ Printing with Fugitive Ink and Pickering Emulsions

**Figure 12:**
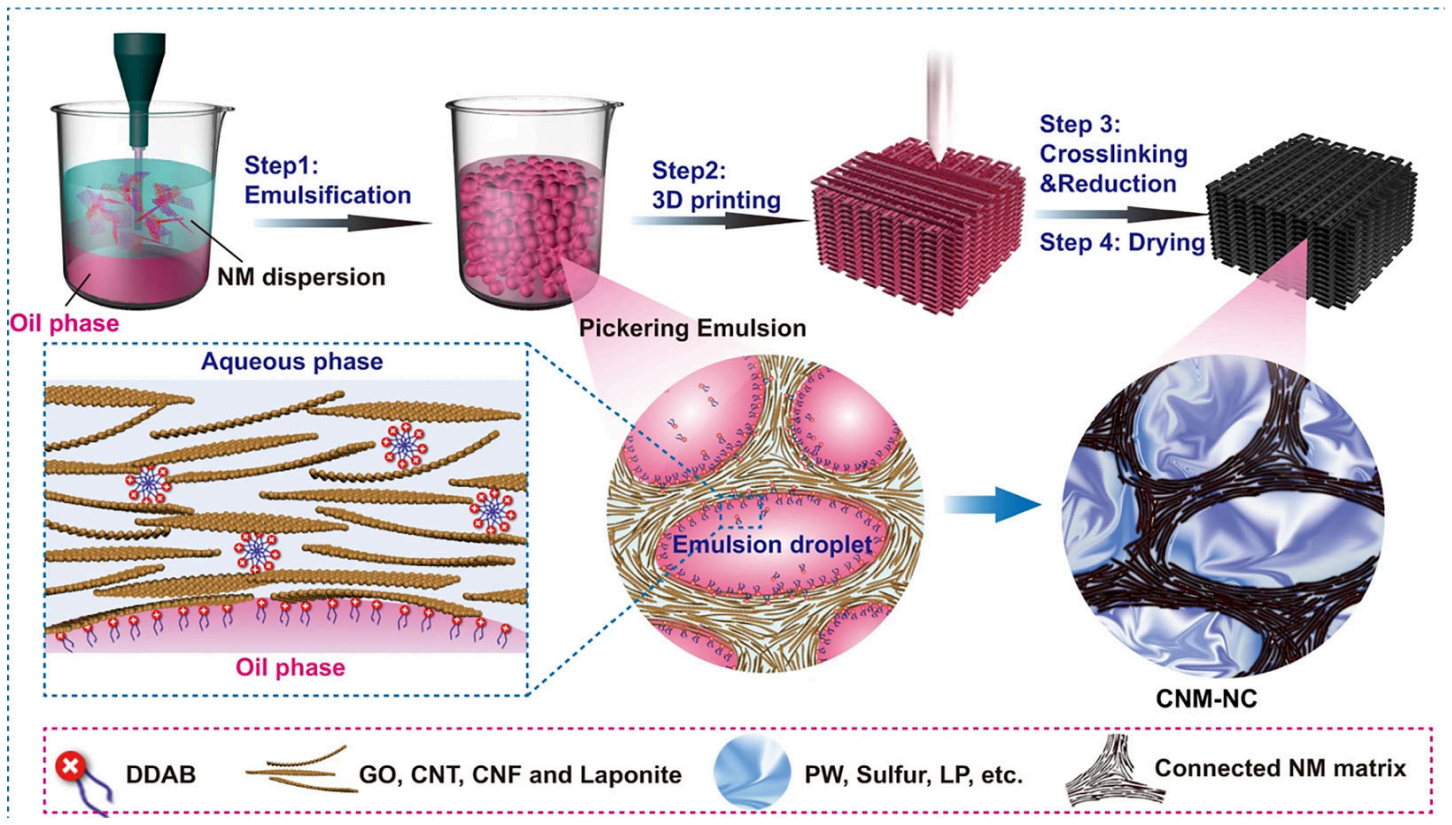
Preparation of Pickering Emulsion [101].

**Figure 13:**
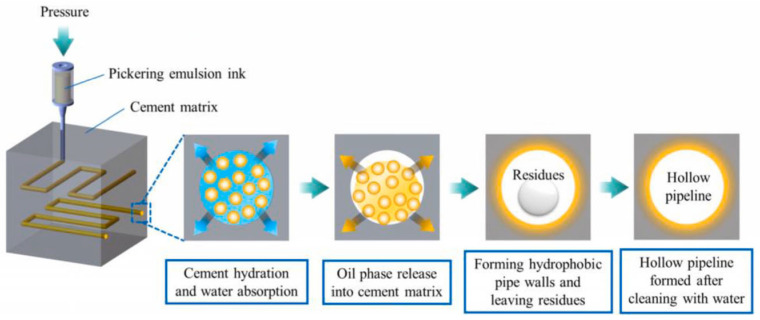
Schematic diagram of Pickering emulsion for in situ printing of vascular channels [106].

As shown in Figure 12, Pickering emulsions can transform the intrinsic physical properties of nanomaterials into 3D topologies and geometries, enabling extrusion molding without additional manufacturing steps (such as photopolymerization or thermoforming) [101,102]. The key requirements for this method are that the emulsion must have sufficient energy storage modulus and exhibit shear-thinning properties [103,104,105]. The high energy storage modulus ensures that the emulsion maintains its shape, and the shear-thinning properties enable it to pass through narrow nozzles during extrusion. Furthermore, as shown in Figure 13 [106], the nozzle can be inserted directly into the cement slurry for printing. Its high storage modulus ensures that the emulsion retains its original shape; at the same time, an appropriate w/c ratio aligns the rheological properties of the cement matrix with the filling path of the injection needle, thereby ensuring the structural integrity of the cement matrix. During curing, the cement gradually absorbs oil and water components from the Pickering emulsion, leading to gradual shrinkage and making the emulsion removable; it can be easily stripped away after 28 days of curing within the cement matrix. The Pickering emulsion should be regarded as a particle-stabilized system: its printability depends on interfacial particle adsorption, emulsion stability, yield stress, and compatibility with the fresh cement matrix.

Zhang et al. [74] developed a system in which sodium alginate served as the continuous aqueous phase, forming a stable emulsion with Tween 80 as the emulsifier. They leveraged the cross-linking properties of carboxylates with cations to maintain the predefined shape within the cement matrix, thereby preventing deformation caused by the matrix’s rheological properties. This led to the stable formation of the vascular system. Building on this, Zhang et al. [73] further developed Pickering emulsions as fugitive inks that retained shear-thinning properties while significantly increasing the energy storage modulus and viscosity. These emulsions did not rely on crosslinking to maintain their shape, enabling multifunctional printing and effective sealing, and could be removed under mild conditions to form a free-form isolated vascular system. Mechanical tests showed that the compressive and flexural strengths of the cement base were reduced after the hollow vascular structures were formed. To compensate for this disadvantage, Zhang et al. [64] also used a carbonization reinforcement method in the vascular area to promote calcite formation, creating a denser structure within a specific range of the vascular area to compensate for the compressive strength.

By removing the two-phase ink, a hollow cement structure was constructed without the need for embedded network templates, overcoming the shortcomings of polymer pipelines, such as reduced ductility and delayed fracture response, and avoiding expansion problems caused by the material’s water solubility. Pickering emulsion printing does not require heating or light curing, thereby reducing printing time and the number of experimental steps. By setting the printing parameters, the vascular size can be flexibly controlled without leakage. Moreover, the oil phase in the two-phase ink can be absorbed by the cement matrix during curing, thereby contributing to the hydrophobic modification of the vascular surface. The only drawback is that like additive manufacturing polymers, current research is still at the laboratory stage due to limitations in printer size. The phase-change temperature of the methyl palmitate oil phase used in two-phase ink is relatively high (28 °C), so it is generally solid under constant laboratory conditions and requires heating before use. Furthermore, while carbonization compensates for compressive strength, it has no significant effect on flexural strength [107]. Enhancement strategies for flexural strength have yet to be determined, and future development should focus on scaling up production, material optimization, and toughening.

#### 3.2.3. 3D-Printed Cement Mortar

Direct ink writing (DIW) is an additive manufacturing technology based on extrusion. When the ink material satisfies the rheological properties, it can be used to print 3D structures [108,109]. Although the rheological properties of cement itself are insufficient to support the existence of hollow vascular structures [110], upon the use of admixtures and fibers [111,112], it is possible to modify the cement matrix to directly serve as ink for printing hollow vascular cement-based materials. Some scholars have attempted this.

**Figure 14:**
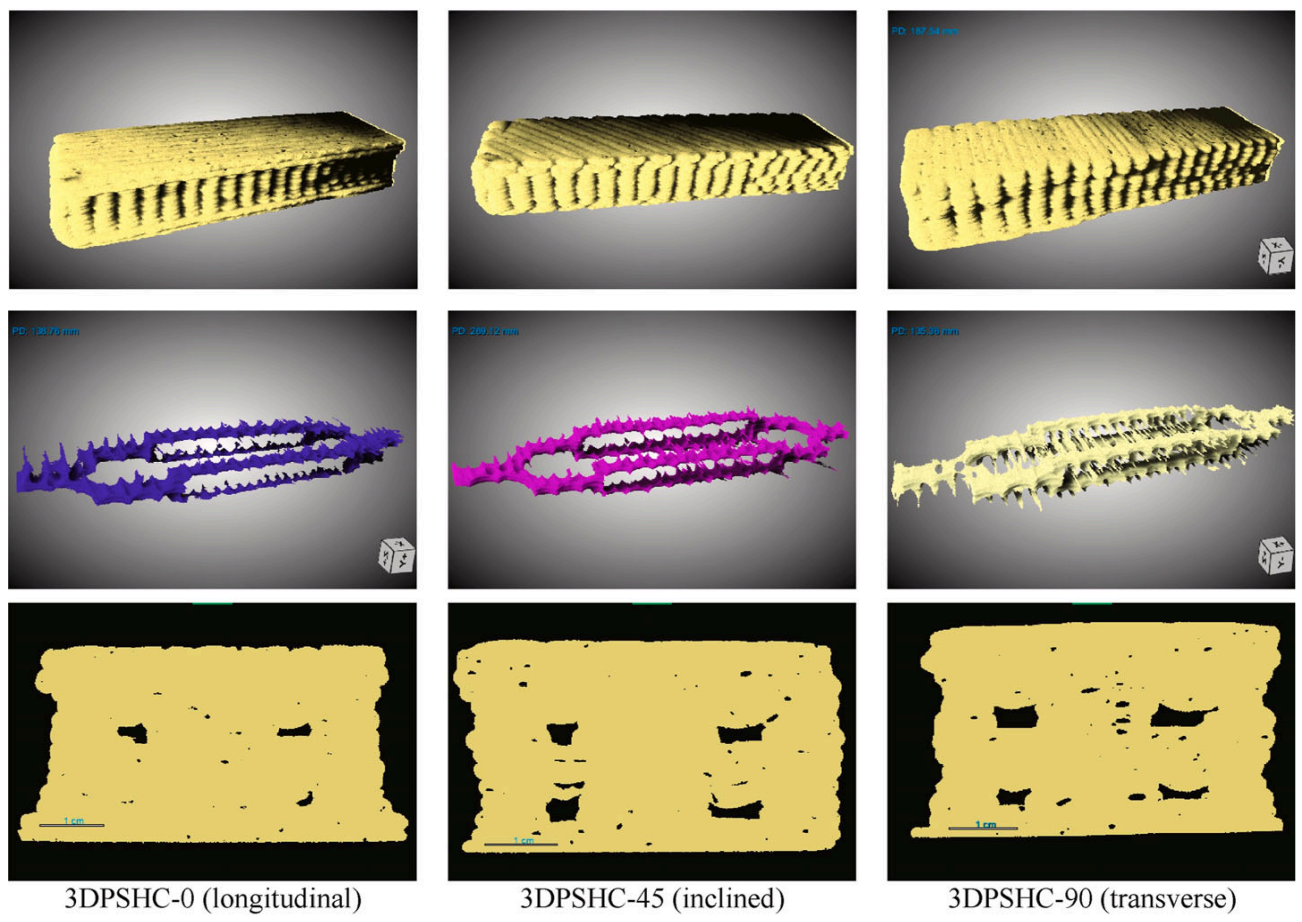
CT reconstruction of hollow vascular channels in 3D-printed cement slurry [76].

**Figure 15:**
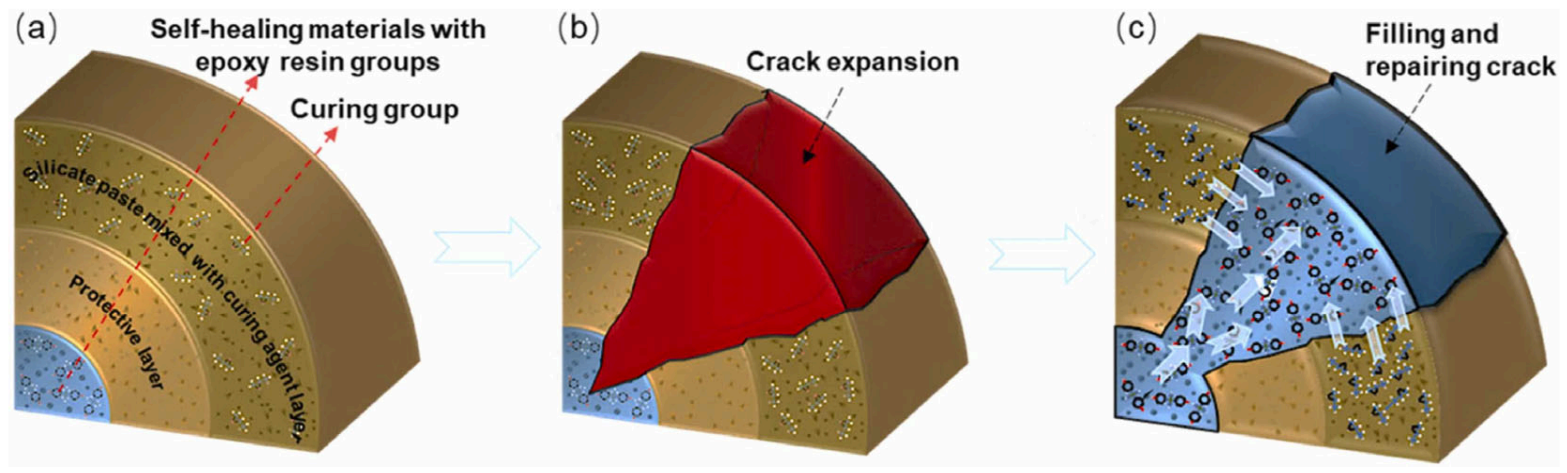
The schematic illustration of the self-healing mechanism: (**a**) Vascular-like multilayer structure; (**b**) Crack propagation reaching the internal healing layer; (**c**) Epoxy resin repairing all cracks after in situ solidification [77].

To achieve direct ink writing with cement paste (Figure 14), Wan et al. [76] adjusted the fluidity of the cement matrix by adding a high-efficiency water-reducing agent and a viscosity-modifying admixture based on hydroxypropyl methylcellulose to the cement material. At the same time, to avoid fragile damage to the test specimens, ultra-high-molecular-weight polyethylene fiber (UHMWPE) was added to the cement matrix to improve its plasticity. Different tests and CT observations were conducted for different printing directions. However, after curing, the channel surface was rough, and the actual channel volume was smaller than the designed volume due to compression of the upper layer. Yuan et al. [77] used a new cement paste printing method, as shown in Figure 6, to develop a multi-component coaxial printing method that simultaneously constructs silicate and epoxy resin components into a self-healing engineering composite material with a biomimetic vascular structure. As shown in Figure 15, when no cracks occur, the epoxy resin is stored in the silicate shell. When cracks occur, the epoxy resin flows into the silicate matrix containing the epoxy resin curing agent, effectively filling the cracks and then solidifying. This method ensures the supply of healing agents while maintaining the morphology of the hollow vascular cement and enhancing its flexibility through an interpenetrating structure.

Direct ink writing technology, which utilizes cement-based materials as printing ink, has excellent prospects [108]. Compared with stereolithography and fused deposition methods for additive manufacturing of polymers, extrusion printing is fast and efficient, and cement is inexpensive [113,114], significantly reducing the manufacturing cost of cement-based materials for hollow vascular structures. Given that 3D printing of concrete has already been used in engineering practice [70,115]. In these systems, polymers serve as functional additives rather than prefabricated pipelines: HPMC improves extrusion performance, dimensional stability, and water retention, while UHMWPE fibers enhance ductility and workability during the printing process. This approach reduces the need for separate polymer templates and is better suited for large-scale construction. Additionally, it presents challenges related to the fiber-cement interface and aging, including the alkali stability of organic additives, fiber-matrix delamination, thermal cycling effects, and the recoverability of cement after hardening. Since it is necessary to simultaneously control rheological properties, layer stability, channel precision, and curing agent delivery efficiency, the manufacturing process remains highly complex; therefore, the practical engineering applicability of this technology still falls short of its potential for large-scale application.

### 3.3. Strategies for the Selection of Polymer-Based Vascular Materials

In selecting polymeric vascular materials, the first step is to distinguish between embeddable networks and removable templates, as they require different levels of stability. Embeddable PLA or ABS networks must maintain structural stability during printing, implantation, and use, while ensuring channel continuity and exhibiting stable crack-induced fracture behavior; in contrast, PVA, P400SR, or emulsion templates need only maintain dimensional stability until the cement matrix can support the channel structure, after which the template must be completely removed without damaging the channel surface. Therefore, high water resistance and strong adhesion are critical for embedded networks, but may conflict with the removability of removable templates. The final selection should be based on intended function, exposure phases, and measurable failure modes, rather than relying merely on printing performance.

For embeddable networks, PLA and ABS provide different combinations of printability, durability, and sustainability. PLA enables precise branched, tetrahedral, and coaxial networks with relatively accessible fused-filament equipment [22,96,97]. Still, its ester backbone is susceptible to alkaline hydrolysis, particularly when moisture and temperature accelerate chain scission. The pH 12 and 12.55 NaOH tests showed that commercial PLA formulations did not behave as one material class, and ultraviolet aging before printing changed orientation-dependent mechanical behavior and water absorption. ABS is less susceptible to ester hydrolysis and generally offers higher toughness and lower water sensitivity during casting [63,94], but its butadiene phase is vulnerable to ultraviolet and thermal-oxidative aging. Because embedded channels are largely shielded from sunlight, their principal service risks are more likely to be creep-induced narrowing, thermal-expansion mismatch, interlayer leakage, and polymer-cement debonding. Filament grade, additives, crystallinity, infill, wall thickness, layer direction, and thermal history should therefore be reported, together with retained flow and trigger reliability after coupled alkaline-moisture-thermal conditioning.

Sacrificial materials require a balance between construction-stage stability and later removability. PVA is printable and water-soluble, but it can absorb water and swell during hydration; complete dissolution of 1 g of printed PVA required approximately 1300 min, and Ca-containing polymer-related products were observed [98]. Wax coating can limit premature swelling [23], but adds to coating quality control and may leave wax or polymer residue. P400SR was reported to avoid the pronounced swelling associated with PVA [99], although its removal completeness and residue remain insufficiently quantified. Pickering-emulsion and emulgel inks permit free-form in situ printing [73,74], but require simultaneous control of yield stress, storage modulus, droplet stability, oil-phase transition, fresh-cement rheology, and complete discharge. For these systems, removal time, residual organic mass, channel roughness, wettability, and post-removal pressure-flow resistance are more relevant than long-term coupon strength.

The polymer-cement interface and trigger mechanism should be explicit selection criteria. A weak annular interface may act as a preferential crack path and cause leakage or premature debonding, whereas an excessively strong interface may divert the crack away from the channel and reduce triggering probability. Thermal-expansion mismatch, polymer creep, cement shrinkage, and repeated loading can progressively change this balance. Whole-beam peak load alone cannot resolve these mechanisms. Embeddable network candidates should therefore be compared using polymer-cement pull-out or interfacial shear strength, interface fracture energy, failure mode, burst or trigger pressure, and pressure-flow retention under dry, saturated, alkaline, and thermally cycled conditions.

Manufacturing complexity, scalability, and sustainability should be evaluated against the repair function delivered. PLA may be bio-based, and both PLA and ABS can be mechanically recycled before use. Still, PLA does not normally biodegrade within concrete, and recovery of either polymer after embedding is unlikely to be practical. PVA routes consume dissolution water and generate polymer-containing effluent, whereas emulsion routes reduce solid template waste but require recovery or disposal of liquid phases. A defensible selection should include printing, joining, positioning, inspection, template removal, cleaning, and end-of-life burdens, together with the expected reduction in repair frequency. Current evidence supports geometric feasibility, but it does not justify a universal material ranking because direct interface tests, long-term functional aging, standardized cost data, and structural-scale production yields remain limited.

### 3.4. Critical Discussion of Vascular Fabrication Strategies

The fundamental differences among vascular fabrication methods arise from the materials used, the channel-forming mechanisms, the healing-triggering modes, and the agent-delivery strategies. Existing methods can be classified into five categories: embedded passive-release systems, continuous channels with active delivery, embedded polymeric networks, sacrificial templates, and directly printed cementitious or multimaterial systems. Embedded glass-fiber tubes are ruptured when a crack penetrates the tube wall, after which the stored healing agent is released. This method is therefore considered a one-time or limited-cycle passive-triggering approach. Continuous channels formed by removing steel tubes or heat-shrinkable tubes are supplied through external ports. Their main advantages are cleanability and refillability [79,90]. Three-dimensional PLA and ABS networks retain embedded polymeric channel walls and provide complex spatial configurations. PVA, P400SR, and Pickering emulsions preserve complex geometries during fabrication and are subsequently removed to form hollow channels. Directly printed cementitious systems with reserved hollow channels and coaxially printed multimaterial systems integrate vascular fabrication with structural-component manufacturing [76,77].

Claims of an optimum vascular diameter are contradictory only when different objectives and normalizations are ignored. In Pickering-printed specimens, increasing channel diameter from 1 to 3 mm increased the reported strength loss from 13.97% to 44.91%, whereas three-dimensional branched networks improved crack coverage, internal filling, and healing relative to one- and two-dimensional arrangements [22,106]. The former comparison isolates the mechanical penalty within a fixed specimen geometry; the latter changes topology, vascular volume, crack-intersection probability, and delivered agent volume. Smaller channels preserve more of the effective load-bearing section but increase flow resistance and blockage risk, while larger channels improve storage and delivery but intensify stress concentration and enlarge the interfacial area. No universal optimum diameter follows from these data. Meaningful comparison requires interpreting diameter together with specimen dimensions, channel number and orientation, vascular volume fraction, healing-agent viscosity, pressure, crack width, and the selected endpoint.

Several common trends can be identified with the development of additive manufacturing. First, vascular fabrication is shifting from fixed and single-use embedded units toward continuous networks that can be cleaned, refilled, and repeatedly used. Second, channel geometries are evolving from one-dimensional regular pathways toward three-dimensional, branched, and free-form networks. Improved crack coverage under uncertain crack conditions is thereby pursued. Third, fabrication is moving from separate channel preparation and implantation toward sacrificial-template printing, in situ fabrication, and integrated component–vascular manufacturing. At the same time, evaluation criteria should move beyond single-cycle crack closure. Initial mechanical loss, repeated delivery capacity, load-bearing recovery, durability recovery, interfacial durability, manufacturing complexity, and maintenance requirements should also be considered. These trends indicate that the future competition among fabrication methods will not depend only on whether a channel can be formed. Greater importance will be placed on whether stable, maintainable, and repeatable healing can be achieved with a limited structural penalty.

The principal barrier to commercialization is the absence of standardized, full-scale, long-term evidence that a vascular system can deliver repeatable healing under realistic loading and environmental exposure without an unacceptable initial or accumulated mechanical penalty. This evidence gap subsumes channel reproducibility, reinforcement conflicts, polymer-cement interface aging, blockage and flushing, agent shelf life and replenishment, crack detection, port accessibility, field quality control, cost, sustainability, and liability. Current studies demonstrate feasibility, but incompatible specimen sizes, diameters, recovery definitions, healing cycles, and exposure conditions prevent reliable technology qualification or a universal fabrication ranking.

## 4. Classification and Mechanism of Healing Agents for Polymer-Enabled Vascular

The self-healing technology for cement-based vascular systems relies not only on the design and manufacture of the vascular system but also on the selection of appropriate healing agents to promptly repair cracks. An ideal healing agent should be a low-viscosity liquid that flows smoothly and penetrates microcracks via pumping or capillary action. It should have suitable curing conditions and time to ensure sufficient mechanical strength after healing. Additionally, the healing agent should be cleanable with air or water for reuse, thereby enhancing durability and restoring strength [69]. However, because the healing effects and mechanisms of different healing agents vary, their classifications and mechanisms of action are used to analyze in depth the healing effects and principles of various healing agents. The curing mechanism, physicochemical characteristics, and maintainability of the healing agents are summarized in Table 5. For polymer-based healing agents, crack sealing should be distinguished from mechanical recovery, as the latter depends on the degree of curing, penetration depth, modulus, interfacial adhesion, and aging in alkaline pore solutions.

### 4.1. Classification of Healing Agents

Currently, healing agents used in self-healing technology are primarily categorized into two types: organic and inorganic. Organic materials mainly cure through polymerization, while inorganic materials primarily involve alkaline silicate solutions that rely on the alkaline environment within cement to complete the hydration reaction. Sodium silicate and mineralizers are classified as inorganic healing agents, while epoxy resins, polyurethanes, cyanoacrylates, and methyl methacrylate are classified as polymer-based healing agents.

#### 4.1.1. Inorganic Substances

Inorganic self-healing materials are typically alkaline silicate solutions, with sodium silicate solutions demonstrating superior performance [116]. Sodium silicate is discussed as an example. As shown in Equation (2), sodium silicate solutions can react with excess Ca(OH)_2_ in the cement Matrix to form calcium silicate hydrate, making them one of the ideal materials for self-healing. Additionally, the healing products from alkaline silicate solutions are consistent with the composition of cement, ensuring good compatibility and eliminating the need to consider interface bonding issues [117]. Furthermore, the low viscosity and dilutability of alkaline silicate solutions enable them to remain stable for extended periods in vascular [118], facilitating transportation and achieving broad coverage.

Na_2_SiO_3_ + Ca(OH)_2_ + H_2_O→2NaOH + C-S-H (2)

In a review by De Belie et al. [7], sodium silicate solution was mentioned as a healing agent. Shields et al. [69] examined the durability and mechanical property recovery of different healing agents (sodium silicate solution, waterproofing agent, polyurethane, and epoxy resin) and compared the applicability of various commercially available healing agents in self-healing vascular systems. In terms of volume recovery, except for sodium silicate solution, all other repair agents achieved sealing efficiencies of over 100%, and polyurethane and epoxy resin demonstrated high-strength recovery. Other researchers also tested volume recovery rates, with alkaline silicates failing to reach 100% [22,73]. In terms of strength, the single-component silicate solution typically achieved a single-cycle strength recovery rate of 8–14% [5,90,96,119]. Many researchers have attempted to enhance the strength recovery of alkaline silicates. Li et al. [22] improved the coverage and reaction space of the healing agent using a 3D vascular design, increasing flexural strength from 20% to 34%. The 20% strength recovery rate here is due to the externally reinforced vascular system with PLA. De Nardi et al. [97] mixed the solution with nano-lime or nano-silica solutions, where the nanoparticles provided nucleation sites for hydration reactions, thereby increasing the flexural strength recovery rate to 25%. In terms of durability, alkaline silicate solutions can seal cracks, reducing water permeability and chloride ion penetration.

Alkaline silicate participates in the hydration reaction of cement, is highly compatible with the cement matrix, offers clear advantages in bonding with cement materials, exhibits strong strength recovery and sealing performance, and has low viscosity, making it easy to transport. It is suitable for repair work with the primary purpose of improving durability [120]. Still, it is not yet sufficient to meet the requirements for strength recovery and fast repair [121], and the strengthening effect of existing reinforcement methods is relatively limited. Future research should focus on improving the durability of alkaline silicate healing agents under different extreme environmental conditions (dry-wet cycles, frost-thaw cycles), developing more advanced vascular systems to optimize the distribution and supply of healing agents, and exploring additive formulations with better strength recovery properties to accelerate the reaction rate of C-S-H formation and achieve better strength recovery effects.

#### 4.1.2. Organic Polymer-Based Substances

Organic materials used for self-healing include epoxy resins, polyurethanes, methyl cyanoacrylate, and methyl methacrylate. These materials repair cracks through polymerization reactions, resulting in high bond strength. Their viscosity can be adjusted through temperature control, additive addition, or formulation modifications, offering high flexibility and broad applicability. Unlike inorganic materials, which require hydration reactions and thus longer curing times, these organic materials cure quickly. They exhibit excellent sealing efficiency and high recovery rates of mechanical properties. Their performance is governed by viscosity, surface tension, wetting of crack surfaces, polymerization or curing behavior, shrinkage, and bond stability at the polymer-cement interface.

For epoxy resins, the flexural strength recovery approaches 100% [62,67,69,122]. Among these, epoxy resins with low elastic moduli demonstrate better repair performance due to their superior toughness compared to those with high elastic moduli [123]. In terms of durability, the high elastic modulus facilitates stress transfer, limiting crack propagation. Samples with crack widths ≤0.5 mm showed complete healing upon ultrasonic testing [62,122]. Epoxy resins exhibit good strength and stiffness recovery after curing, making them suitable for repairing areas with high strength requirements. However, epoxy resins containing bisphenol A are highly toxic and may have adverse environmental impacts [124]. Additionally, UV aging alters the physical and chemical properties of epoxy resin, and a decrease in adhesion directly reduces protective performance. Therefore, before classifying an epoxy resin vascular system as a durable material, comprehensive testing must be conducted on the adhesion between the epoxy resin and cement, ultraviolet exposure conditions before and after crack healing, and resistance to erosion by alkaline pore solutions.

For polyurethane, bending strength recovery exceeds 50% [125,126]. Shields et al. [69] achieved load recovery of 215% using a high-pressure injection method with a two-component polyurethane. Hu et al. [127] improved strength recovery to 75% by adding acetone to dilute polyurethane, thereby adjusting surface tension and viscosity. In terms of durability, polyurethane undergoes a polymerization reaction upon contact with water, resulting in a volume expansion of 25–30 times its original size. This effectively fills cracks and achieves sealing efficiency exceeding 100%, thereby enhancing water resistance. Acoustic emission and ultrasonic velocity testing indicate that structural integrity is improved after crack bonding [72,126]. Polyurethane has the characteristic of rapid expansion, increasing its volume by 25–30 times its original size, enabling it to fill cracks quickly. It is suitable for scenarios requiring rapid repair, such as large cracks and complex crack patterns, as well as for temporary reinforcement after disasters. However, it has poor high-temperature resistance and tends to age under intense ultraviolet light [128]. Future developments could include developing UV-resistant polyurethanes, modifying polyurethanes with UV stabilizers, and integrating these modifications with sustainable development to produce biodegradable polyurethanes.

For cyanoacrylate (CA), strength recovery can reach 90% [129]. De Nardi et al. [130] mixed cyanoacrylate with acrylic acid (AA) and nitro anthraquinone (nAq) in different ratios to form a series of modified cyanoacrylates (n-CA), finding that the n-CA formulation with a ratio of 4:1:0.02 (CA:AA:nAq) 02(CA:AA:nAq) significantly increased from 14 days to 21 days, with a maximum value of 2.6 MPa. De Nardi et al. [131] further mixed CA with acrylic acid at a 3:1 ratio, achieving a 24% increase in flexural strength load recovery compared to commercial CA while reducing the viscosity of the healing agent. In terms of durability, CA’s rapid initial setting effectively seals cracks and enhances water resistance. De Nardi et al. [131] also conducted numerical modeling using an optimized, modified n-CA formulation and successfully predicted that n-CA can effectively close cracks. CA has low viscosity, good permeability, and favorable rheological properties and cures rapidly, enabling sound repair quickly and making it suitable for complex, rapidly changing crack conditions. However, excessive curing speed may cause premature closure of the crack front, resulting in an insufficient supply of healing agent to the rear end and affecting the healing effect. Future developments can explore new CA formulations, optimize existing formulations, and model CA-based healing flow and healing to control healing speed, thereby ensuring a consistent supply of the healing agent.

For methyl methacrylate (MMA), the flexural strength of repaired concrete beams reached 130% of the original strength under standardized conditions, with a deflection of 0.5 mm [79]. In terms of durability, the three-component MMA filled the cracks after healing, with the healed structure resembling organic glass, being dense and enhancing water resistance. MMA has a viscosity similar to that of water, allowing it to flow into small cracks, and it exhibits strong resistance to extreme temperatures. However, its drawbacks include flammability and low toxicity.

### 4.2. Mechanism of Healing Agents

Many mechanisms trigger the action of healing agents, such as capsule rupture and exposure to moisture and air. The mechanism in vascular systems is relatively simple, typically relying on pumping and capillary action to initiate curing upon reaching the crack area. The use of healing agents in vascular systems is explained in terms of their diffusion and curing mechanisms.

#### 4.2.1. Capillary Diffusion Mechanism

Capillary action is the primary mechanism by which healing agents participate in crack sealing [132]. The healing agent flows through the crack and penetrates its surface due to the combined effects of capillary action, gravity, and surface tension. The flow efficiency of capillary action depends on the vascular diameter [72], external environment (temperature, ultraviolet rays) [133,134], crack geometry (crack width and shape) [132], and physical properties of the healing agent [69] (curing characteristics, viscosity, surface tension with cement, and contact angle).

Abyaneh et al. [135] considered cement as a heterogeneous composite material with a discrete cubical lattice and proposed a three-dimensional numerical study of capillary absorption to simulate the capillary water absorption process of cement as a heterogeneous composite material, verifying that the nonlinear diffusion equation and the finite element method can effectively simulate the capillary water absorption process. Gardner et al. [136] studied capillary flow in discrete cracks using experiments and a modified Lucas-Washburn equation, developed a numerical program, and incorporated modifications to established capillary flow theory to account for the stick-slip behavior of the meniscus and friction dissipation at the meniscus-wall interface. Huang et al. [137] used Hagen-Poiseuille’s law to analyze the relationship between capillary action and the viscosity of healing agents. They found that surface tension and contact angle are key factors affecting the flow efficiency of healing agents and that surface tension and viscosity have an exponential relationship [137].

The reviewed studies show that healing agent transport has mainly been quantified by adapting classical fluid-mechanics models to describe capillary penetration and pressure-driven flow in cementitious cracks. The main difficulty is not the number of available equations but the inconsistency in their assumptions and parameter definitions. The Lucas–Washburn equation is generally used for spontaneous imbibition in ideal capillaries or simplified uniform cracks. Modified forms are required when crack roughness, contact-line stick–slip, dynamic contact angles, wall slip, or additional friction are considered. The Hagen–Poiseuille relation is usually limited to fully developed laminar flow of constant-viscosity Newtonian fluids in regular channels. Darcy-type models are more suitable when cracked regions are treated as equivalent permeable media. Navier–Stokes or CFD models are required for irregular and coupled channel–crack geometries, but their predictions are sensitive to constitutive laws and boundary conditions. In addition, the same parameter may be defined differently. Crack width may refer to surface width, mean geometric width, or effective hydraulic aperture. Contact angle may be static, advancing, or dynamic. Viscosity may denote an initial, apparent, or curing-dependent value. These inconsistencies hinder direct comparison among studies.

A standardized framework should be established for model selection. The driving mechanism should first be identified as capillary flow, pressure-driven flow, or a combination of both. The applicability, required corrections, and failure conditions of the Lucas–Washburn, Hagen–Poiseuille, Darcy-type, and Navier–Stokes/CFD models should then be defined according to the crack representation, fluid rheology, flow regime, and boundary conditions. At a minimum, key information should include crack width, crack roughness, vascular geometry, temperature, moisture condition, viscosity, contact angle, curing time, pumping pressure, and transport distance. The governing equations, principal assumptions, and boundary conditions should also be clearly stated. To improve cross-study comparability, transport distance, filling ratio, flow rate per unit pressure, and dimensionless parameters such as the Reynolds number should be reported using consistent definitions. For non-Newtonian healing agents, a universal critical Reynolds number should not be proposed unless the crack geometry, characteristic length scale, rheological model, curing state, and boundary conditions have been clearly defined.

#### 4.2.2. Pressurized Flow Diffusion Mechanism

Pressurized flow actively drives the healing agent, increasing its driving force and enabling it to fully cover the damaged area, even in complex, wide cracks. In addition to early packaging technology, the self-healing process of the vascular system generally results from the dual effects of pressure flow and capillary action. Pressure flow delivers the healing agent, while capillary action ensures that small cracks are effectively sealed.

Compared with solutions that rely solely on capillary action, the pressure-flow solution significantly increases the number of healing cycles and overall healing efficiency. Davies et al. [90] studied the effect of pressure flow on healing efficiency and found that it improves the healing of 0.2 mm cracks, enabling multiple flushes and redelivery of the healing agent. Hamilton et al. [138] explored various pumping strategies and found that the volume required to achieve excellent healing performance in vascular systems was significantly reduced when using pressure- flow. Moreover, pressure flow is more effective for low-viscosity healing agents. Shields et al. [69] and Qamar et al. [139] found that high pressure can reduce vascular blockages. However, when the supply pressure is too high, it has the opposite effect, causing the healing agent to flow out. Selvarajoo et al. [140] found that a pressure flow of 0.3 bar yielded the best healing results, whereas healing deteriorated when the supply pressure was too high (>0.5 bar). Precise pressure control requires determining the appropriate friction coefficient equation for each healing agent, as they exhibit different critical Reynolds numbers under the same pressure-delivery conditions. Zirjawi et al. [141] used computational fluid dynamics to select seven suitable models from numerous fluid mechanics equations and obtained good predictive results for the two best solutions. Freeman et al. [142] developed a coupling model based on the Navier-Stokes equation crack-flow model to simulate the pressure-driven flow of the healing agent in the vascular and successfully predicted its distribution and solidification in the crack at 0.5 bar.

Based on the above literature review, it can be concluded that applying pressurized flow enhances healing efficiency, flushes the vascular, prevents blockage of the healing agent, and enables multiple uses. However, excessive pressure may cause the healing agent to leak from the crack, thereby reducing the healing rate. Additionally, different healing agents have distinct properties, and their flow states are not uniform. Future developments should consider the healing efficiency of different healing agents under various pressures, determine the critical pressure at which healing is optimal, and observe the repair process under different pressures. Using computational fluid dynamics, the Reynolds numbers governing the transition from laminar to turbulent flow of different healing agents at the microscopic level should be investigated, and methods for modifying the fluid mechanics equations under dynamic contact angles should be explored.

#### 4.2.3. Curing Mechanism of Polymerization Reactions

When cracks form, the healing agent is released from the piping system and flows to the damaged area, gradually curing as it flows and repairing the damage. One method of curing is to trigger polymerization using ions.

Epoxy resin curing primarily occurs via two mechanisms: anionic and cationic [143]. In the anionic mechanism, epoxy groups can be opened in different ways to generate reactive anions. In the cationic mechanism, epoxy groups can be opened by active hydrogen to form new bonds and hydroxyl groups. In cement self-healing applications, epoxy resin curing is mainly an anion-initiated addition polymerization reaction [144]. Curing agents have two or more reactive groups per molecule, which react with epoxy groups and are directly incorporated into the curing system as structural components of the polymer. Multifunctional amines, amide regenerators, acid anhydrides, polyphenols, and polycarboxylic acids are commonly used as co-reactive curing agents. Wang et al. [145] used TEPA (tetraethylenepentaamine) to cure epoxy resin, with the nitrogen atom acting as a nucleophile to initiate anionic polymerization, thereby significantly improving bonding to the cement matrix. Sun et al. [146] used molecular dynamics to study the interaction between a load-induced C-S-H hydrogel and epoxy resins with different crosslinking densities and found that increasing crosslinking density increases interfacial porosity, thereby affecting the adhesive properties and subsequent mechanical behavior of the material. Long-term performance is governed more by the cured epoxy-cement interface than by bulk resin strength. Although crosslinked epoxy can show good resistance to alkaline pore solution, water uptake can plasticize the network, weaken polar interactions with hydration products, and promote interfacial separation; ultraviolet exposure is most relevant to exposed or near-surface repairs, whereas buried repairs are more sensitive to hydrothermal and thermal-cycle damage. Curing shrinkage and thermal-expansion mismatch can further concentrate stress at the crack walls. After curing, epoxy is generally neither flushable from narrow channels nor practically recyclable because it is a thermoset. Repeated use therefore requires separated components, delayed mixing, or parallel delivery paths, and the service-life benefit should be balanced against channel blockage, amine/BPA handling, and end-of-life separation.

Polyurethane (PU) is mainly composed of diphenylmethane diisocyanate (MDI) and polyether polyol, in which the isocyanate reacts with water, mainly from the water content of the cement itself, to produce foam that fills cracks and hardens. In very humid environments, the foaming reaction of PU healing agents is not solely determined by the cement’s moisture content. Philip et al. [147] observed through visual assessment of neutron radiography images obtained from capillary adsorption tests that both high-viscosity and low-viscosity healing agents could prevent water from almost completely entering the crack zone. But the foamed product is difficult to remove from the vascular network. Long-term durability is strongly formulation-dependent: polyester-based soft segments are generally more susceptible to hydrolysis than polyether-based systems, while heat, oxygen, and ultraviolet radiation can change foam stiffness, cell structure, and brittleness; high alkalinity and repeated wet-dry cycles may also alter adhesion to cement. Embedded repairs are shielded from most ultraviolet exposure, but near-surface cracks and external ports are not. Because cured PU foam has limited recyclability and conventional flushing is often ineffective, repeated-healing designs should verify aged pressure-flow retention, blocked-channel fraction, foam residue, and bond or sealing retention rather than assuming that an expandable sealant remains reusable.

Highly reactive cyanoacrylate monomers (CA), such as ethyl cyanoacrylate, undergo anionic polymerization at room temperature with trace amounts of water or amines as catalysts, which absorb sufficient water to catalyze polymerization. The chemical reaction proceeds very rapidly, leading to rapid bonding. Not only can it penetrate large cracks, but it can also penetrate the microcrack regions in the fracture process zone. Joseph et al. [148] found that ethyl cyanoacrylate resin provides advanced healing in small crack widths (<0.5 mm) and in the presence of hydroxide ions (obtained from moisture within the crack plane). Selvarajoo et al. [140] reported a cured CA tensile strength of 21 MPa, far exceeding the tensile strength of the cement matrix (3.4 MPa), which helps explain why re-cracking may shift away from the healed interface. However, CA is less suitable for long-term wet and highly alkaline exposure than its rapid initial bond might suggest. Moisture and hydroxide ions initiate polymerization but can also promote post-cure hydrolysis, while the stiff local repair may progressively embrittle and transfer damage to the surrounding cement. Thermal cycling and differential expansion can further weaken the narrow bonded interface. The small delivered volume reduces material consumption, but the cured polymer is not recoverable from the crack or channel.

**Figure 16:**
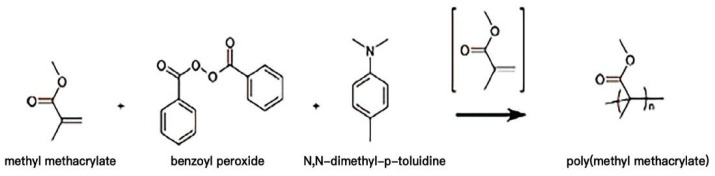
Polymerization reaction of methyl methacrylate initiated by benzoyl peroxide and N,N-dimethyl-p-toluidine [150].

Methyl methacrylate monomer (MMA) undergoes free radical polymerization under the action of two initiators, isopropyl peroxide and cobalt neo decanoate, and hardens to the hardness of organic glass within 30 min [68]. However, mixing isopropyl peroxide and cobalt neo decanoate simultaneously can cause an explosion, and the three-component healing agent is difficult to polymerize at room temperature. Tittelboom et al. [149] optimized the three-component formulation by using benzoyl peroxide (BPO) as the initiator and N-dimethyl-p-toluidine (DMPT) to activate the redox reaction and generate BPO free radicals, thereby eliminating the need for heating. As shown in Figure 16, BPO free radicals react with MMA monomer molecules and bind to one end of the MMA molecule [150], while another free radical originates from the other end of the molecule. Then, the latter free radical can react with another MMA molecule, and the chain polymerization proceeds in this manner. The cured PMMA product has high density and excellent mechanical properties, and its UV weather resistance is generally better than ABS or many polyurethane formulations; however, the combined effect of strong alkalis and high temperatures may still trigger a slow ester hydrolysis reaction. Polymerization shrinkage and a high coefficient of thermal expansion significantly increase the risk of interfacial voids and thermal cycling fatigue. Although PMMA is a thermoplastic material and can theoretically be recycled as bulk material, recovery from confined cracks or cement pore networks is impractical, and the cured material is difficult to expel from the channels.

#### 4.2.4. Curing Mechanism of Chemical Reactions

Curing mechanisms resulting from contact with cement usually depend on calcium ions and alkaline hydroxide ions in the cement matrix. Converting calcium hydroxide (CH) to C-S-H is beneficial to the material as a whole because the presence of CH is harmful to the chemical and mechanical durability of cement. In addition, CH is water-soluble and susceptible to acid erosion, and the surrounding interface is usually highly porous, increasing permeability and reducing strength. Therefore, using a curing agent to absorb CH is also an effective strategy for protecting the cement itself [151].

Sodium silicate reacts with calcium hydroxide (CH) in the presence of water to form calcium silicate hydrogel (C-S-H), the main product of cement hydration, which is considered an excellent mineral candidate for self-healing of cement materials. Kanellopoulos et al. [152] confirmed through X-ray diffraction analysis of hardened cement mortar, cement samples with added sodium silicate, and cement samples with added 4% L500 microcapsules and 4% T130 microcapsules that the addition of sodium silicate leads to the consumption of CH and the formation of C-S-H in the reaction between healing substances and the cement matrix. Hu et al. [153] found that TEA addition induces C-S-H to arrange sequentially and form a sponge-like structure. The formation of TEA-Ca promotes the dissolution of solid Ca from hydration products, thereby increasing the chemical reactivity of the hydrated cement matrix. Additionally, the increased density of C-S-H enhances the actual bonding capacity.

### 4.3. Strategies for the Selection of Polymer-Based Healing Agents

Polymer-based healing agents should be selected through a coupled transport-cure-interface assessment. The relevant question is not whether the cured polymer is strong but whether the liquid can be stored without premature reaction, delivered through the selected channel and crack, cured within the required intervention time, and retain a durable bond under the crack’s moisture and alkalinity. Crack sealing, load recovery, and durability recovery are different targets. Table 6 summarizes the reported healing properties and scope of action.

Epoxy and polyurethane illustrate the trade-off between structural bonding and rapid sealing. Low-viscosity, adequately mixed epoxy generally provides the strongest basis for restoring crack-face adhesion and load transfer, but its curing time, shrinkage, and wet-interface sensitivity must be controlled. Moisture can plasticize cured epoxy and compete with cement-surface interactions; calculated cement-epoxy adhesion decreased by 37.49% and 72.02% at 3% and 6% interface moisture, and concrete-epoxy interface fracture toughness decreased by up to approximately 50% under moisture-temperature conditioning. PU is more appropriate for rapid filling of wet, wide, or irregular cracks because moisture-triggered foaming expands into voids. Still, foam stiffness, adhesion, leakage, and channel blockage are formulation-dependent. Polyester-based PU is generally more hydrolysis-sensitive than polyether-based PU, and heat, oxidation, and ultraviolet exposure can promote embrittlement. Thus, epoxy is more defensible for structural recovery when one-time or separately routed delivery is acceptable, whereas PU is more defensible for rapid water sealing when expansion and aged flow resistance are verified.

Cyanoacrylate and MMA occupy the low-viscosity end of the selection range, but their long-term limitations differ. Cyanoacrylate rapidly polymerizes in moist or alkaline cracks. It can penetrate fine fracture-process-zone cracks, yet the same trigger can cause premature hardening in the carrier or closure near the crack entrance. Commercial cyanoacrylate polymerized in PLA microvascular units after approximately 72 h, while formulation modification delayed hardening and produced a pull-off bond strength of 2.6 MPa after 21 days. After curing, moisture and alkalinity can promote hydrolysis and embrittlement, and the stiff bonded zone may shift re-cracking to the surrounding matrix. MMA has water-like viscosity and high penetration potential, but unmodified MMA can exhibit substantial polymerization shrinkage; fillers can reduce shrinkage while increasing viscosity and potentially reducing bond performance. Cured PMMA-like material generally has good weatherability, yet thermal-expansion mismatch and thermal shock can reduce interface stability. MMA is therefore appropriate only when penetration benefits outweigh shrinkage, flammability, and multi-component storage risks.

The polymer-cement interface should be treated as an independent selection variable rather than inferred from bulk polymer strength. Bonding can involve mechanical interlocking, wetting, hydrogen bonding, and Ca-O or Ca-N interactions with hydration products, but crack moisture, polymerization shrinkage, interfacial porosity, creep, and thermal mismatch can progressively reduce adhesion. Candidate agents should be screened using pull-off or tensile bond strength, interfacial fracture energy, permeability, and failure mode under dry, saturated, alkaline, wet-dry, and thermally cycled conditions. For PU, the contributions of adhesion and foam filling should be separated; for cyanoacrylate and MMA, brittleness, shrinkage, and re-cracking adjacent to the healed zone should be recorded. Short-term load-regain indices should not be interpreted as directly comparable material properties when precracking level, curing time, and calculation method differ among studies.

Safety, environmental burden, repeatability, and end-of-life behavior further narrow the choice. BPA-related epoxy components and amine hardeners require controlled handling, PU formulations may involve isocyanates, unreacted MMA is volatile and flammable, and cyanoacrylates can rapidly bond skin and generate localized heat. Cured epoxy, PU, and CA are not practically recoverable or recyclable from cracks, while thermoplastic PMMA is theoretically recyclable only as a separated bulk material, not after confined curing within cement. Sustainability claims should therefore include production, unused-agent disposal, cleaning or flushing, blockage frequency, and avoided maintenance rather than relying on polymer type alone. On the present evidence, epoxy is best supported for structural bonding, PU for rapid wet-crack sealing, CA for controlled rapid penetration of small cracks, and MMA for very-low-viscosity delivery where shrinkage and fire safety can be managed. This is an application-dependent ranking, not a universal hierarchy.

### 4.4. Critical Discussion of Healing Agents

The literature does not establish a universal optimum healing agent concentration. The apparent optima reflect a transport-reactivity trade-off and inconsistent meanings of concentration. More concentrated silicate or nanoparticle-modified suspensions increase reactive solids and nucleation sites but may also increase viscosity, sedimentation, and blockage; dilution of polyurethane can improve penetration and recovery, whereas excessive dilution reduces polymer mass and final stiffness [97,127]. Modified cyanoacrylate formulations with CA:AA:nAq = 4:1:0.02 and CA: AA = 3:1 improved working time or flexural recovery relative to commercial cyanoacrylate, but they optimized different endpoints [130,131]. For two-component epoxy and MMA, component stoichiometry and crack-level mixing are more decisive than nominal concentration; insufficient mixing eliminated clear epoxy healing in d-TET networks [97]. Concentration therefore cannot be separated from supplied volume, channel diameter, pressure, viscosity evolution, curing onset, and crack geometry.

For structural applications in which recovery of load transfer is the primary criterion, low-viscosity epoxy has the strongest overall evidence. It can develop high crack-face adhesion, stiffness and strength recovery, and complete watertightness in several pressure-injected or ABS-network studies [62,67,69,122]. This ranking remains conditional: epoxy underperforms when its components do not mix, when curing is too rapid for delivery, or when moisture, shrinkage, and thermal mismatch weaken the interface [97,146]. Polyurethane performs better for rapid sealing of wet, wide, or irregular cracks because moisture-triggered expansion fills voids. Still, its formulation-dependent foam stiffness, leakage, and channel blockage weaken its case for durable structural load transfer [69,72,126,147]. Sodium silicate has the best chemical compatibility with cement and is more defensible for durability-oriented repair, but its typical mechanical recovery remains below that of polymer adhesives even after network or nanomaterial enhancement [22,97]. Cyanoacrylate and MMA provide exceptional penetration and rapid or high initial recovery, respectively, but premature polymerization, hydrolytic embrittlement, shrinkage, flammability, and multi-component handling restrict broader structural use [79,130,131,148].

The cross-study consensus is that healing performance is governed by the coupled vascular-agent-cement system rather than by cured-polymer strength alone. The vascular network controls storage, delivery, mixing, and spatial coverage; the agent controls penetration, curing, shrinkage, bonding, and environmental retention. Crack closure is therefore distinct from mechanical recovery and durability recovery, and first-cycle performance is distinct from repeated delivery. Epoxy, polyurethane, and MMA frequently block channels after curing; cyanoacrylate can harden prematurely; and silicate systems are generally cleaner but provide lower mechanical recovery. The conflict between high first-cycle recovery and poor repeatability is a system-level limitation, not an inconsistency in healing-agent chemistry.

The evidence supports an application-dependent hierarchy: epoxy for structural bonding, polyurethane for emergency or wet-crack sealing, silicate or organic-inorganic hybrids for durability and cement compatibility, cyanoacrylate for controlled penetration of fine cracks, and MMA for very-low-viscosity delivery where shrinkage and safety are controlled. No agent has yet demonstrated standardized, multi-cycle structural recovery after long-term alkaline, wet-dry, thermal, and fatigue exposure at full scale. The strongest short-term repair agent is therefore not automatically the most commercially viable system.

## 5. Evaluation Method for Self-Healing Cement Healing Effect in Vascular

For cement materials, regardless of the self-healing solution adopted, appropriate evaluation and observation methods are necessary to assess crack sealing and elucidate the healing mechanism, thereby demonstrating that the cement can again bear loads and prevent the intrusion of corrosive media. Vascular self-healing solutions are no exception. Traditional mechanical performance testing methods and microstructural and visualization analysis can clearly reflect the feasibility of vascular systems as a self-healing method and, to a certain extent, reflect the performance requirements of vascular systems as self-healing solutions, such as the rationality of vascular location, the effect of vascular volume fraction or related parameters on mechanical properties, and the bonding strength between the healing agent and the cement interface. Table 7 summarizes the typical characteristics of various evaluation methods in vascular self-healing.

### 5.1. Healing Ability Evaluation

The assessment of healing performance includes mechanical properties and durability, reflecting the compensatory and enhancing effects of the self-healing method on mechanical properties, the effectiveness of vascular transportation, the excellent bonding properties of the healing agent, and the quantitative weakening effects of vascular parameters on the mechanical properties of cement-based materials. For polymer-based healing agents, the evaluation should also consider the cured polymer modulus, interfacial adhesion, and long-term stability of the healed crack.

#### 5.1.1. Destructive Mechanical Property Evaluation

Three-point and four-point bending tests are most commonly used to evaluate the loading capacity of cement before and after healing. The difference between the two is that the three-point bending test introduces a notch at the point of maximum bending moment in the beam span, thereby generating a single crack. In contrast, the four-point bending test produces multiple cracks during continuous loading. It has been widely used to evaluate the healing efficiency of strain-hardening cement materials, which exhibit multiple cracks under continuous loading [154]. Bending tests on self-healing cement materials for vascular applications are conducted to evaluate the effectiveness of vascular parameters and vascular locations in conveying healing agents by recovering bending strength and stiffness and assess the repair performance of the healing agent [155]. To some extent, the integrity of cement materials can be evaluated through compressive strength, which reflects internal porosity and density; higher component density generally correlates with higher compressive strength.

Therefore, compression tests are widely used to evaluate the healing efficiency of self-healing cement. Compression tests on vascular self-healing cement-based materials can quantify the effect of the vascular self-healing system admixture on the mechanical properties of cement materials—for example, Zhang et al. [64] tested the impact of different vascular diameters and load directions on the compressive strength of test specimens. The results showed that when the load was applied perpendicular to the channel direction, a larger diameter further decreased strength.

Uniaxial tensile testing is also a method for assessing healing effectiveness [156], with tensile strength reflecting the bonding between healing products and the cement matrix at the macro level [131]. For vascular self-healing cement materials, the addition of a vascular system increases the requirements for the healing agent’s performance, particularly in curing and fluidity. Crack generation induced by notches creates conditions for the healing agent to be used. After healing is complete, uniaxial tensile tests are performed to evaluate the bonding performance of the healing agent and the cement matrix. For example, Selvarajoo et al. [140] measured the tensile strength of cured CA to be 21 MPa, which is much stronger than the tensile strength of cement (3.4 MPa), indicating that the test piece will not crack after being re-bonded and cured by the healing agent, and re-cracking will not occur around the healing area. It can be seen that the CA curing and cement matrix bonding form a cement-based polymer composite material with higher strength than the original cement.

At the macro scale, bending, compressive, and tensile tests constitute a standard system for evaluating the practicality of self-healing cement-based materials and healing agents for vascular systems. With the continuous development of testing technology, microhardness testing at the microscopic level can assess the microstructure and strength of cement mortar by measuring indentation and interfacial response in the transition zone, and has the potential to become a means of evaluating the self-healing performance of vascular [157,158,159]. This view was inspired by Zhang’s [64] research, in which her team demonstrated that carbonation enhances the mechanical properties of vascular channel walls via microhardness testing. Therefore, exploring reinforcement strategies for vascularized cement requires not only verifying their feasibility through macroscopic mechanical testing but also elucidating the specific mechanisms underlying performance improvements by observing the microscopic interfacial transition zone.

#### 5.1.2. Nondestructive Mechanical Property Evaluation

In addition to destructive mechanical performance tests to obtain specific numerical values for strength and characterize self-healing effects, acoustic testing methods (acoustic emission and ultrasonic pulse velocity) can also be used to assess crack-healing efficiency [71,167,168]. In acoustic emission testing, transient elastic waves (stress waves) are released when cement is damaged (crack initiation and propagation) or the vascular wall ruptures. These waves are then captured by a sensor, and the healing efficiency is evaluated by comparing the energy before and after healing [125,154]. However, noise interference in the test environment must be controlled. In ultrasonic pulse-velocity testing, because short ultrasonic waves propagate faster in cement than in water or air, an increase in transmission time indicates the presence of cracks and defects, and a decrease in transmission time after healing confirms healing. However, temperature changes in the test environment and moisture in the cement must be taken into account [160].

With the latest advancements in monitoring systems, a composite monitoring system combining acoustic emission (AE), ultrasonic pulse velocity (UPV), and digital image correlation (DIC) techniques has been developed [71,72] to dynamically monitor damage and healing in small-volume cement materials and track the entire field, complementing each other to assess healing efficiency. Combining the repeatable self-healing properties of pipe self-healing cement materials with a dynamic integrated monitoring and sensing system, cracks can be accurately located after they generate, and the extent of damage can be assessed, ensuring that the healing agent is continuously supplied at the right time and place to achieve accurate and repeatable autonomous crack repair, further optimizing healing efficiency and cost-effectiveness and broadening the scope of intelligent material design.

#### 5.1.3. Durability Evaluation

The appearance of cracks can allow moisture and corrosive ions to penetrate the cement, corrode the reinforcing steel, weaken mechanical properties, and adversely affect the cement’s long-term performance. Therefore, we will evaluate durability in terms of watertightness and ion permeability. The healing effect is primarily assessed by whether water or corrosive substances penetrate before and after repair and is usually measured using water permeability and water absorption tests [125,161]. It is sometimes assessed through rapid chloride penetration tests to assess ion permeability levels [162]. For vascular self-healing solutions, durability testing not only reflects the vascular system’s repeatability to some extent but also indicates adhesion between the healing agent and the cement matrix, as well as resistance to ion penetration. Based on the results of durability tests, the rationale for vascular design and the performance of the healing agent can be clearly understood. Short-term crack closure alone is insufficient to demonstrate long-term durability improvement; permeability, ion ingress, wet-dry cycling, freeze-thaw resistance, and aging of the polymer-cement interface should be considered.

### 5.2. Analysis of Healing Mechanisms

Using visualization methods, it is possible to intuitively see the distribution and effects of healing agents at the microscopic level, the vascular layout, and the dense structure around the vascular diameter, clearly showing vascular morphology that is difficult to observe with the naked eye. Chemical bond detection and elemental analysis methods help explain the healing mechanism and perform content analysis.

#### 5.2.1. Visualization

**Figure 17:**
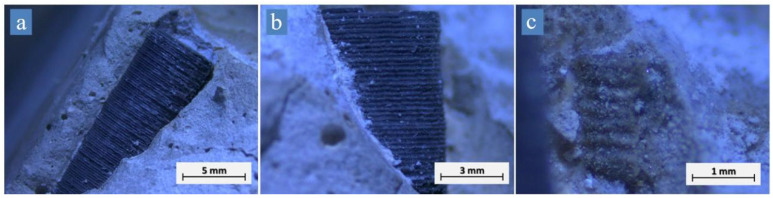
Microscopic image ((**a**) 5 mm, (**b**) 3 mm, (**c**) 1 mm) of the connection between the polylactic acid vascular system and the cement matrix [22].

Optical microscopy is an effective method for directly observing crack-surface repair products and assessing crack-surface closure [163]. When combined with image processing software, it can quickly quantify the crack-sealing rate. However, this observation is limited to the surface layer, whereas vascular self-healing typically occurs internally, making it difficult for microscopes to observe internal conditions. In such cases, microscopy is generally used for transportation system evaluations, such as the adhesion between vascular systems made of non-sacrificial materials and cement matrices (Figure 17) [22,97], the swelling behavior of water-soluble pipe materials during the curing process of cement [98,99], and the stability of volatile ink or Pickering emulsions [73,74].

For internal observation, X-ray computed tomography (X-CT) is generally used. X-CT is a non-destructive imaging technique that uses X-rays to penetrate samples from multiple directions, obtain projection data, and reconstruct 3D images of the internal and external structures [169]. It can be used to understand the internal conditions of the areas surrounding the vascular system, such as the distribution of healing agents [22,98], crack formation [64], and the internal connectivity of the vascular system [63,64,76,99]. It is worth noting that subsequent 3D visualization requires specialized software (e.g., ImageJ, DeVIDE).

Neutron beams can easily penetrate cement-based materials but are strongly attenuated when they encounter hydrogen atoms, making them suitable for visualizing water-molecule absorption and distribution before and after cement repair [164,165]. This technique can be used not only to study the healing efficiency of self-healing cement materials but also to investigate the influence of embedding a vascular system on water capillary absorption in cement matrices [147].

**Figure 18:**
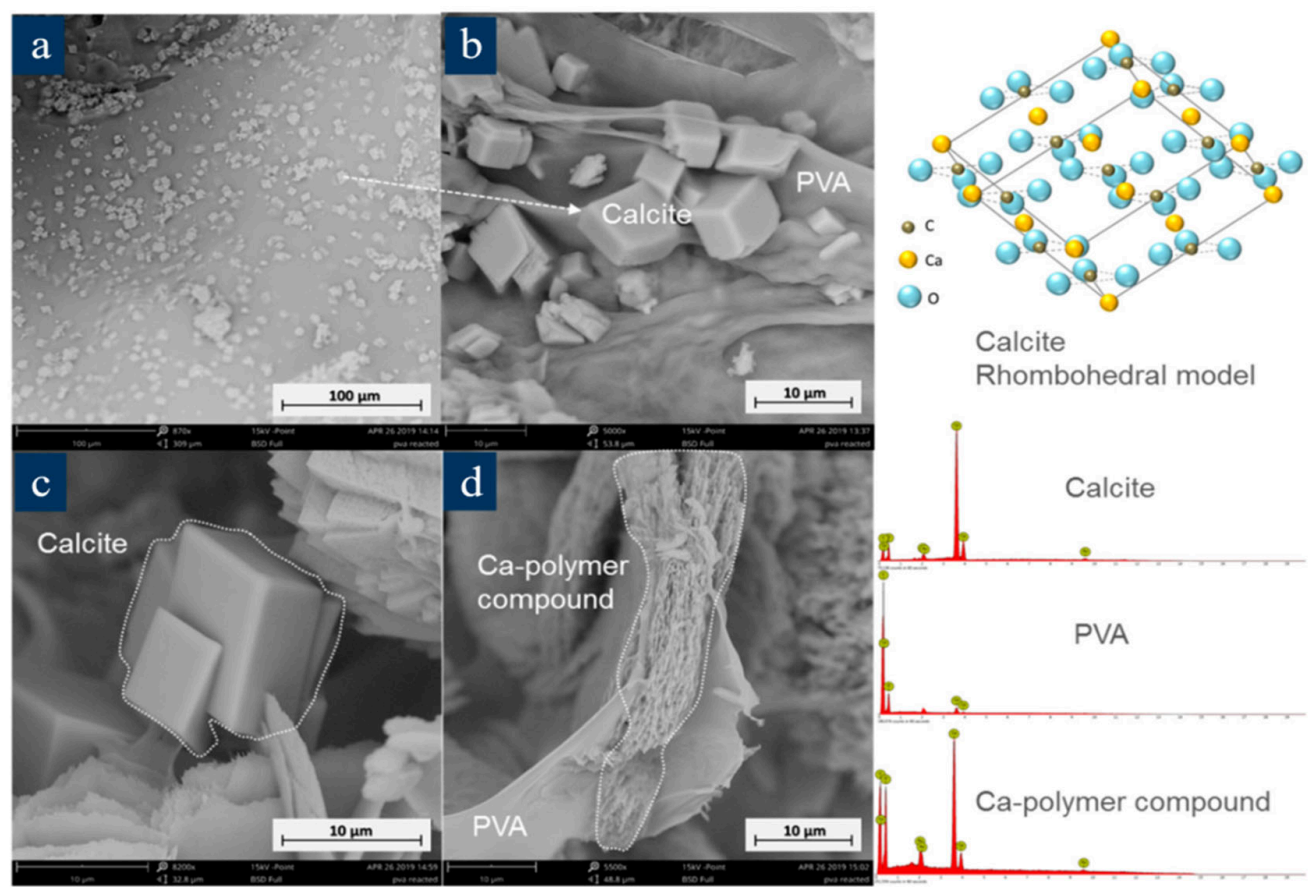
SEM images: (**a**) Expanded calcite mineral on polyvinyl alcohol, (**b**) Magnified image of polyvinyl alcohol and calcite, (**c**) Calcite crystal, (**d**) calcium-polymer composite formed on polyvinyl alcohol and EDS analysis of the reaction between polyvinyl alcohol and cement matrix [98].

The combination of scanning electron microscopy (SEM) and energy-dispersive X-ray spectroscopy (EDS) is also commonly used to visualize and analyze healing products [166]. While SEM alone can only indicate the presence, distribution, and crystal morphology of healing products, it cannot determine their specific composition. Therefore, EDS testing is required to provide chemical-composition analysis and further confirm the elemental composition and distribution of healing products. This composite detection method can be used to infer the formation of calcite or C-S-H hydrogel after healing, as well as the interaction between the tube system and the cement matrix. As shown in Figure 18, Wan et al. [98] used SEM and EDS to detect a composite composed of the tube-manufacturing material, polyvinyl alcohol, and Ca+ cations in the cement matrix.

#### 5.2.2. Elemental Composition

Spectral analysis can record the spectrum of light passing through healed or unhealed cement samples, revealing the location and intensity of absorption peaks. Then, the chemical composition of the healing products can be determined through a database. X-ray diffraction (XRD) or Fourier transform infrared (FTIR) spectroscopy is commonly used [64,98]. The two methods differ in specific ways, and using a single method has certain limitations. When polymers are used as vascular systems and alkaline silicates as healing agents, the two approaches should be combined for vascular self-healing. The specific reasons are as follows: XRD is primarily based on crystal diffraction according to Bragg’s law, where diffraction peaks at particular angles are generated by atomic planes in the crystal lattice, and it cannot be used to analyze amorphous materials; FTIR relies on detecting characteristic absorption peaks from different chemical bonds or functional groups, reflecting molecular vibrations. The former primarily consists of inorganic products, such as calcite, calcium aluminate, and calcium silicate hydrate. At the same time, the latter mainly comprises organic healing agents with characteristic functional groups, including polyurethane and acrylic esters. From the above, it can be seen that FTIR testing is required to observe the bonding products between the vascular and the cement matrix in non-sacrificial polymer vascular systems [98]. However, when the healing agent is an inorganic substance, FTIR cannot be used on its own. For other cases, appropriate characterization schemes can be flexibly selected based on the detection mechanisms of XRD and FTIR.

Thermal analysis is also an effective method for elemental composition analysis, as different substances exhibit distinct high-temperature decomposition ranges. The composition of target products is determined by the presence of mass loss peaks within specific temperature ranges. Standard detection methods include thermogravimetric analysis (TGA) and differential scanning calorimetry (DSC).

## 6. Challenges and Prospects for Polymer-Enabled Vascular Self-Healing Systems

To date, although the design and application characteristics of vascular self-healing in ordinary silicate cement matrix at a small-scale laboratory level have gradually matured, research on its applicability to large-volume cement and other specialized cement-based materials (such as geopolymer concrete and self-compacting concrete) remains inadequate. There is also considerable scope for further development in applying the most promising AI technologies to crack detection and predicting healing efficiency. In the previous paragraphs of this article, we provided a detailed overview and commentary on the design, manufacturing, and application of vascular self-healing. In the following paragraphs, we will summarize and explain the shortcomings and challenges in developing vascular self-healing and explore suitable directions for future development.

### 6.1. Challenges of Bionic Vascular Self-Healing Systems

(a) The current combination of Murray’s theorem and construction laws optimizes flow paths, reduces flow resistance, and expands coverage. Still, it has not addressed surface non-homogeneity in cement materials and is limited to vascular design itself. Research on the capillary action of healing agents and pressurized flow remains confined to linear vascular systems, and studies on the flow of non-Newtonian fluids in cement are still lacking. In summary, the vascular design has not achieved optimal flow.

(b) The combination of artificial intelligence technologies such as reinforcement learning, deep neural networks, and genetic algorithms has achieved accurate healing efficiency prediction, optimization of the optimal vascular layout position, and excellent iterative performance. However, there remains a lack of effective finite element models, resulting in insufficient data. This is because the existing models predict layout positions based on locations prone to bending and cracking, whereas the actual design combines the Murray theorem with the range of vascular coverage.

(c) The system introduced to enhance the synergy between the vascular system and the cement matrix still has defects. The introduction of the vascular system increases porosity and alters stress distribution, thereby reducing the mechanical properties of the cement. To compensate, researchers have made numerous attempts, including incorporating reinforcing materials—such as steel bars, fibers, and shape-memory alloys—or using biomimetic coaxial printing to manufacture self-healing mortar with embedded vascular. However, adding reinforcing materials reduces the volume of vascular layout and restricts vascular flexibility, while the toughening method of biomimetic coaxial printing relies on repair after the first failure. Future mitigation strategies should focus on reducing adverse effects and improving the material’s damage tolerance, rather than eliminating the stress concentrations caused by hollow channels.

(d) During the use of glass fiber-encapsulated healing agents, the distribution and brittleness of fragile glass materials become more pronounced, and large-scale application may lead to increased costs. Although incorporating ECC (engineered cement composite) or SMA (shape memory alloy) technology can improve repair effectiveness and operating conditions, the high cost remains an issue.

(e) Self-diagnostic methods rely on temperature for activation; although they offer higher controllability, the heating process may have specific effects on cement-based materials. Additionally, the heating system requires an external power supply, which increases costs and resource consumption beyond the cement itself, making it unsuitable for power-free environments.

(f) The steel bar extraction method and sacrificial materials in linear vascular are feasible for large-scale applications and concrete. However, the extraction process may cause structural damage. Additionally, the 1D linear design sacrifices vascular flexibility and creates new ingress points for corrosive media.

(g) Additive manufacturing pipe systems are limited by printer size and lack scalability. Layered printing may create gaps that cause cement leakage. Additionally, hollow network structures reduce the mechanical properties of the cement, necessitating improvements in printing quality to minimize porosity and enhance adhesion at the concrete matrix interface.

(h) The 3D-printed concrete with reserved piping system retains the low cost of cement while leveraging the freeform printing capabilities of additive manufacturing, demonstrating significant potential for application. However, modifications to the cement’s rheological properties are insufficient, and the formation of the piping system remains suboptimal.

(i) The water-conducting modification of pipes using Pickering emulsion relies on the release and absorption of methyl palmitate components during the exothermic hydration process. However, at normal laboratory temperatures, methyl palmitate is solid, making it inconvenient to use.

(j) Alkaline silicate solutions have good compatibility with cement matrices and a clear advantage in bonding with cement-based materials. However, they have slower healing rates and lower strength recovery than polymer adhesives, making them suitable only for repair work primarily intended to improve durability. They are insufficient to meet the requirements for strength recovery and rapid repair.

(k) Organic adhesives are diverse but have their own advantages and disadvantages. Bisphenol A in epoxy resins is toxic, polyurethanes degrade under UV light, methyl cyanoacrylate exhibits unpredictable curing rates that can cause blockages, and methyl methacrylate requires a multi-component formulation. Additionally, these polymer-based healing agents should be evaluated for viscosity, curing shrinkage, wettability, alkali stability, and long-term aging performance, rather than merely their ability to restore short-term strength.

(l) The fluid dynamics equations used for healing agents are dispersed and lack uniformity. Significant differences in dynamic parameters and equations, coupled with changes in fluid properties, render the equations inapplicable.

### 6.2. Prospects

(a) Long-term durability and service condition verification after healing: Although the pipeline self-healing method demonstrates a high healing rate in terms of short-term crack closure and strength recovery, its engineering reliability and long-term performance have not yet been proven. Durability factors such as degradation of the polymer pipeline wall, delamination of the polymer-based healing agent and the interface, repeated self-healing, healing agent performance under wet-dry and freeze-thaw cycles, thermal exposure, and the durability of the polymer system under chloride or sulfate corrosion still need to be evaluated. Full-scale, long-term validation is required to determine whether self-healing performance is maintained under actual service conditions [170,171,172].

(b) Scalable Manufacturing: Polymer vascular networks, Pickering emulsion in situ printing, and polymer-cement co-printing technologies offer considerable geometric flexibility. However, due to printer size limitations, issues such as the fabrication of pipeline systems in large-scale structures or concrete test specimens, channel accuracy, interlayer defects, detachability, and on-site reproducibility remain unaddressed, while the application of pipeline systems in large-scale structures is crucial for practical engineering applications. Therefore, research should focus on stable manufacturing processes and quality control methods for pipeline systems in large-scale structures [173,174,175,176,177,178].

(c) Mechanically compatible vascular structure design: Due to the reduction in effective bearing area and the stress concentrations that concrete generates in the pipeline region when subjected to loads, hollow channels inevitably alter crack propagation behavior and reduce structural mechanical performance. Therefore, the design should comprehensively consider factors such as the vascular geometry, orientation, diameter, adhesion between the polymer and cement, reinforcing materials, and cement toughening. Particular attention should be paid to flexural strength, tensile strength, and fracture energy, as effective compensation strategies for these parameters remain limited at present. Combining cement toughening technology with the self-healing mechanism of vascular conduits holds promise as a viable solution [179].

(d) Integrating sensing and delivery: Pipeline self-healing methods are more efficient than approaches such as microcapsules and microorganisms, but they cannot automatically release healing agents, such as pH-triggered release from microcapsules or oxygen-triggered release from microorganisms. The pumping of healing agents still requires human intervention. Therefore, future vascular self-healing cements should incorporate features such as online monitoring, sensor-based damage detection, AI-assisted crack identification, and controllable microfluidic delivery as sensing mechanisms. Specifically, fiber-optic Bragg grating sensors and distributed fiber-optic sensing can capture crack initiation in real time, locate damaged areas, and track strain or temperature evolution. Recent research on fiber-optic sensing monitoring for smart infrastructure also indicates that hybrid sensing networks can provide reliable data for damage diagnosis and healing decisions [180]. By integrating these sensing technologies into vasculature self-healing cement, the healing agent can be delivered precisely when and where needed, thereby improving healing efficiency and reducing unnecessary material consumption [181,182,183,184].

(e) Green and Sustainable Multifunctional Vascular Networks: Against the backdrop of the “Dual Carbon” goals and low-carbon development throughout the entire infrastructure lifecycle, future vascularized self-healing cement-based materials should not only focus on healing efficiency but also consider the environmental impact of vascular materials, healing agents, and manufacturing processes. Priority should be given to developing bio-based, recyclable, low-toxicity, and waste-derived materials for network fabrication, interface regulation, and healing agent selection to reduce resource consumption, potential toxicity, and disposal burden associated with traditional petrochemical polymers [185,186]. At the same time, vascular networks—characterized by continuity, directed transport, and repeatable replenishment—can be further coupled with various repair mechanisms [187,188] and extended to functions such as heat exchange, phase-change energy storage, temperature regulation, and structural condition monitoring. By integrating self-healing, thermal management [11,99], and sensing functions into a single network, the need for additional components and repetitive maintenance can be reduced, thereby improving material utilization efficiency and the structural value over the entire service life. Therefore, adopting green materials and multifunctional integration not only helps reduce the environmental impact of vascular networks themselves but also serves as a key pathway to enhancing their engineering economics and practical appeal.

## 7. Conclusions

This review comprehensively summarizes the latest advances in poly-based bionic vascular self-healing cementitious materials, including network design, manufacturing technologies, the selection and mechanisms of action of healing agents, and performance evaluation. Research indicates that with the development of additive manufacturing, Pickering emulsion in situ printing, and cement-based multi-material synergistic printing, vascular fabrication is gradually shifting from the production of standalone, pre-implanted structures to continuous networks; however, mechanical performance degradation caused by hollow channels, limited scalability of manufacturing methods, and a lack of practical engineering validation remain major challenges to their commercial application. The key conclusions drawn from this review are as follows:

- Compared to microcapsules and microbial self-healing, the main advantage of vascular self-healing lies in the greater directionality, controllability, and repeatability of healing agent delivery. It can continuously supply healing agents to cracks via capillary action, gravity, or external pressure, and enables multiple rounds of repair through flushing and reinjection, thereby overcoming the limitations of single-trigger activation in capsules and the slow, environment-dependent nature of microbial healing.
- These channels reduce the effective loading area and create stress concentrations near the channel walls and at the polymer–cement interface, thereby reducing flexural strength and compressive strength.
- Among existing manufacturing methods, direct printing of cement-based materials and coaxial multi-material integrated printing of cement-polymer composites offer the greatest potential for large-scale production. These methods allow for the simultaneous construction of a vascular network during component formation, reduce the need for prefabrication and embedding steps, and are compatible with existing 3D-printed concrete technologies; however, issues such as channel deformation, interlayer compression, and material rheological matching still need to be addressed.
- Different healing agents are suitable for different purposes. Epoxy resins are better suited for restoring structural loading capacity, polyurethanes are suitable for quickly sealing wide and irregular cracks, and silicates are better suited for repairs that prioritize cement compatibility and durability. In the future, efforts should focus on comprehensively optimizing the viscosity, curing time, interfacial adhesion, and repeatability of these repair agents.
- Future research should focus on designing vascular systems that balance regenerative capacity with mechanical properties. On the one hand, the loss of mechanical properties caused by hollow channels should be reduced by optimizing pipeline diameter, spatial positioning, branch configurations, and volume fraction, or by employing cement modification strategies such as fiber reinforcement, nanomaterial modification, and matrix densification. On the other hand, manufacturing methods for pipelines suitable for large-scale concrete components should be explored, promoting the development of direct printing, in situ sacrificial templates, and multi-material collaborative printing, with a focus on addressing issues related to channel continuity, forming accuracy, construction stability, and on-site quality control.

## Figures and Tables

**Table 1:** Design principles and auxiliary methods for self-healing vascular-based systems.

Principle/ Method	Purpose	Applicability	Results	Disadvantages	References
Murray’s Law	Optimize vascular layout to reduce fluid transportation energy consumption.	Newtonian fluid with low viscosity based on Poiseuille flow	The crack healing rate in 3D networks was 36% and 23% higher than in 1/2D networks	Ideal conditions are homogeneity	[20,21,22,23]
Construction law	Optimize grid structure	Non-Newtonian fluids and complex fluid environments	Extending Murray’s Law from energy minimization in branched networks carrying ideal Newtonian fluids to non-Newtonian fluids	Grid density affects mechanical properties	[24,25,26,27,28]
Genetic algorithm	Predict healing efficiency	Multi-parameter processing	Quantifying the effects of factors such as w/c ratio, biological repair materials, and crystalline components on crack healing capacity	High data dependency, slow iteration	[29,30]
Deep neural network	Optimize vascular arrangement	Large data sets	Find the vascular location with the least impact on strength: Test R^2^ > 0.895; average peak load/toughness/hybrid target increased by 17.31%/34.16%/9.51%	Decreased accuracy of multifactorial prediction	[31]
Reinforcement learning	Reduce the adverse effects of vascular on cement	Suitable for scenarios with a broad design space and complex objectives	Through iterative interaction with the finite element model, RL automatically adjusts the position and number of pipelines to obtain a layout that yields a higher peak load or fracture energy.	Data simulation takes time due to reliance on interactive simulation environments.	[32,33]

**Table 2:** Mechanical properties of cement containing a vascular network.

Vascular Network	Compressive Strength	Flexural Strength
Encapsulated fiberglass	Not reported; only load-deflection testing was performed.	Not reported; only load-deflection testing was performed.
Thermoplastic organic film pipe	Not reported; primarily characterizes heat responsiveness	Not reported; primarily characterizes heat responsiveness
Linear vascular	Not reported, but deflection, stress-strain, and CMOD were tested.	Not reported, but deflection, stress-strain, and CMOD were tested.
Polymeric vascular networks	For polylactic acid pipes, the peak load increased from 5 kN to 10.1–13.2 kN due to the reinforcing effect of the steel reinforcement; however, the flexural strength of polyurethane and acrylonitrile-butadiene-styrene materials both decreased.	Considering branch and pipeline lengths, no compressed pressure test reported
Pickering emulsion in situ printing	Pipe diameters of 1, 2, and 3 mm result in a decline in flexural strength of 13.97%, 26.09%, and 44.91%, respectively.	Losses range from 3.07% to 21.43%; the impact is significantly exacerbated in 3 mm pipelines.
3D-printed cement mortar	Vertical printing provides the highest flexural strength, while horizontal printing provides the lowest; no control group without piping.	Not reported; all were printed as concrete beams.

**Table 3:** Summary of reported healing indicators and outcomes for representative vascular fabrication methods.

Fabrication Method	Representative Study and Healing Agent	Healing Indicators	Reported Healing Efficacy or Research Focus
Encapsulated fiberglass	Methyl methacrylate in hollow glass fibers	Dye/liquid release, permeability, flexural or compressive response, and qualitative crack filling	Verified active/passive triggering and internal liquid release. Standardized load, strength, sealing, or water-tightness recovery ratios were not defined
	Low-viscosity epoxy in hollow fibers combined with superelastic SMA wires	Cyclic load-deflection response, second-loading cracking load, and re-cracking location	Second-loading cracking load increased by about 28.6% and 21.2%. The result reflects the coupled effect of SMA crack closure and epoxy bonding, not epoxy alone.
	PSS-ECC with glass tubes and low-viscosity ethyl cyanoacrylate	Stiffness recovery, crack relocation, and multiple cracking behavior	The study emphasized stiffness recovery and crack redistribution rather than a single peak-load recovery ratio because ECC strain-hardening can affect the apparent reloading response
Thermoplastic organic film pipe	Ethylene-vinyl acetate thermoplastic film tube containing one-component thermosetting epoxy resin	Electrical resistance change, heating temperature-time response, film melting, epoxy release, and water leakage	Cracking increased resistance from about 69.6 to 76.7 ohm; selective heating reached the film melting temperature and enabled epoxy release
Linear vascular	Steel-rod-formed injection network with moisture-curing epoxy	Cyclic load-displacement response, residual crack width, ultrasonic transmission, original-crack reopening and new-crack formation	Near-full strength recovery was reported after epoxy injection and curing within several days
	Three-part methyl methacrylate system	Load-displacement or flexural response, crack bonding, polymerization, and deflection-controlled recovery	The repaired beams reached up to about 130% flexural strength under deflection-controlled conditions
	Refillable two-dimensional vascular network; sodium silicate and cyanoacrylate-related systems	Healing of about 0.2 mm cracks, pumping, flushing, repeated delivery, flexural or load recovery, and channel maintainability	Sodium silicate showed better pumpability and cleanability but lower mechanical recovery, whereas cyanoacrylate could reach up to about 90% strength recovery in pressurized vascular systems
	Glass-tube vascular comparison using sodium silicate, polyurethane (PU1/PU2), silane-based waterproofing agents, and two-component epoxy.	Load-regain index, sealing efficiency, crack-surface coverage, channel blockage, and cleaning by compressed air	Epoxy, PU2, and PU1 reached load-regain indices of about 257%, 195%, and 155%, respectively; PU1 increased to about 215% after high-pressure flushing. Sodium silicate showed 62% sealing efficiency, whereas PU1 showed about 144% and remained about 139% after high-pressure flushing
	Reservoir-vascular tube network with polymeric healing agent	Acoustic emission, digital image correlation, ultrasonic pulse velocity, strength recovery, toughness recovery, and repeated repair	Strength and toughness recovery exceeded 80%, and ultrasonic pulse velocity increased markedly after healing
Additive manufacturing of polymeric vascular networks	1D, 2D, and 3D PLA vascular networks supplied with sodium silicate	Surface crack closure, X-CT internal healed volume, load recovery, and sorptivity reduction for about 0.7–0.8 mm cracks	Surface crack closure was about 52%, 69%, and 81% for 1D, 2D, and 3D networks; internal healed volume was about 36%, 49%, and 72%; load recovery was about 20%, 20%, and 34%; sorptivity reductions were 25%, 69%, and 77%
	3D-printed ABS vascular networks with epoxy healing agent	Load-CMOD or flexural response, toughness, crack pattern, permeability, and water-tightness recovery across two healing cycles	Flexural healing efficiency exceeded 150% under the study-specific definition, and water-tightness recovery reached 100%.
	3D-printed PLA tetrahedral mini-vascular networks (TETs) filled with sodium silicate	Load-CMOD, strength recovery, stiffness recovery, fracture-energy recovery, fracture-surface coverage, and repeated damage-healing cycles	In the first cycle, two- and three-TET specimens reached about 19% and 20% strength recovery, with stiffness recovery of about 75–79%. Later cycles showed a clear decline.
	D-TET networks storing separate components; epoxy/hardener, sodium silicate, sodium silicate/nanosilica, and sodium silicate/nanolime were compared	Crack triggering at about 0.35 mm, load-CMOD response, strength recovery, stiffness recovery, and two-component release	Sodium silicate/nanolime gave the highest strength recovery, about 25%; sodium silicate alone was about 17%, and sodium silicate/nanosilica about 20%. Bi-component epoxy did not give clear healing when crack-level mixing or stoichiometry was insufficient.
	3D-printed PVA twin-twisted sacrificial vascular tunnels	PVA water absorption, swelling, dissolution, channel formation, SEM/EDS, and matrix cracking risk	No standardized mechanical or sealing recovery was reported. The main result was demonstrating complex PVA tunnel formation.
	Ductile-porous versus brittle 3D-printed vascular networks with polyurethane	Load recovery, stiffness recovery, non-destructive evaluation, channel state, and network ductility	Reported load recovery reached about 56% and stiffness recovery about 91% in the best cases
In situ printing with fugitive ink and Pickering emulsions	Freeform embedded printing using Pickering emulgel fugitive ink	Emulsion rheology, storage modulus, printing stability, template removal, channel connectivity, and liquid transport	The study demonstrated printable and connected channels for healing-agent transport, but did not report standardized load, strength, crack-closure, or water-tightness recovery ratios
	Programmable emulsion-based vasculature using sodium alginate/Tween 80	Ink printability, shape retention, free discharge, channel morphology, and water-based agent transport	Comparable mechanical or permeability recovery percentages were not reported
	MMT/CMC-stabilized Pickering emulsion; moisture-reactive polyurethane healing agent	Flexural-strength recovery, compressive recovery after pre-damage, crack sealing for 100–400 micrometer cracks, water absorption, permeability.	Flexural-strength recovery was about 12.24–17.61% after 1 d and 32.49–39.48% after 3 d for 1–3 mm channels. Low pumping speed gave compressive recovery of about 89.89%, 94.06%, and 92.56%. Sealing efficiency exceeded 98% at 200 micrometers and averaged above 74% over 100–400 micrometers.
3D-printed cement mortar	Direct ink writing of cementitious channels with epoxy healing agent	Four-point bending, flexural healing efficiency, permeability, X-CT channel morphology, channel volume, and printing-direction effects	Study-specific flexural healing efficiency exceeded 100%, but cured epoxy blocked the rough channel walls, so only one effective healing cycle was obtained. The work therefore links recovery to channel quality and clogging rather than only to epoxy strength.
	Coaxially printed silicate-epoxy hybrid system; flowing epoxy component A and component B	Repair of 20–350 micrometer cracks, compressive stress-strain response, maximum compressive strength, crack filling	The system repaired cracks of about 20–350 micrometers, and the maximum compressive strength after healing was reported to increase by about 50% compared with the initial undamaged silicate specimen

**Table 4:** Engineering properties of different vascular fabrication methods.

Fabrication Method	Manufacturing Complexity	Cost	Scalability	Practical Potential
Encapsulated fiberglass	Low; tubes are prefabricated and directly embedded, but fragile during casting	Moderate; low device cost but high manual placement demand	Low; tube distribution and crack–tube intersection are difficult to control	Low; mainly suitable for proof-of-concept and small specimens
Thermoplastic organic film pipe	High; requires sensing/heating and pipe melting control	High; heating system and power supply increase cost	Low; external power and selective heating limit field use	Low; controllable triggering is attractive, but system complexity is high
Linear vascular	Moderate; one-dimensional channels are simple, but ports and removal must be controlled	Moderate; simple molds/channels, but maintenance ports are needed	High; suitable for accessible or prefabricated components	High; best near-term route for refillable and maintainable systems
Additive manufacturing of polymeric vascular networks	High; requires printing, positioning, coating/removal or interface control	High; polymer printing and post-treatment increase cost	Moderate; geometry is flexible but printer size and placement limit scale-up	Moderate; strongest quantified evidence, but scale-up and long-term polymer interface remain unresolved
In situ printing with fugitive ink and Pickering emulsions	High; emulsion stability, rheology, printing and removal must be controlled simultaneously	High at current stage; formulation and in situ printing control are complex	Low; high design freedom but still laboratory-stage	Moderate; high geometric freedom, but mechanical and healing benchmarks remain limited
3D-printed cement mortar	High; depends on cement rheology, buildability, channel accuracy and layer stability	Moderate; cement itself is cheap, but printing equipment/process control is costly	High; conceptually closest to construction-scale printing	High potential; promising for integrated construction, but not yet engineering-ready

**Table 5:** Healing agent curing mechanisms and workability, including polymer-based and inorganic systems.

Healing Agent	Healing Mechanism	Physical and Chemical Properties	Applicability	Disadvantages	Maintainability
Alkaline silicate	Hydration reaction	Cement-compatible and alkali-stable; low handling risk, but UV/thermal resistance is rarely reported	Repair work aimed primarily at improving durability	Not sufficient to support strength recovery and rapid healing requirements	Water/air flushing is possible in refillable channels, but C–S–H precipitation may gradually block channels and slow repeated healing
Epoxy resin	Anionic polymerization reaction	Alkaline and thermal stability are formulation-dependent; BPA-based systems raise toxicity and handling concerns	Healing of areas requiring high strength	Bisphenol A is toxic	Generally not cleanable after curing; repeated use requires separate delivery paths or delayed mixing, with shrinkage and interface aging as concerns
Polyurethane	Isocyanate foaming reaction	Moisture-reactive and expandable, but sensitive to UV/high temperature and involves isocyanate-related handling risks	For situations requiring rapid healing, large cracks, and complex shapes	Poor high temperature resistance, prone to aging under UV light	Cleaning is difficult after foaming; high-pressure flushing can reduce blockage, but repeated use is limited by foam residue and UV/thermal aging
Cyanoacrylate	Anionic polymerization reaction	Moisture/alkalinity-triggered rapid curing; long-term UV/thermal stability is unclear, with brittleness and handling concerns	Situations requiring quick repairs	Excessively fast curing causes the front end of a crack to close, affecting the healing efficiency	Poor repeatability because rapid curing can block channels; no reliable post-curing cleaning method is usually available
Methyl methacrylate	Free radical polymerization	Low viscosity and rapid curing after initiation; however, uncured MMA exhibits flammability risks, requiring careful preparation and handling of multi-component mixtures	Extreme temperature conditions	Flammable and low toxicity	Limited repeatability because cured MMA/PMMA is not readily flushed; long-term concerns include shrinkage, flammability, and multi-component storage.

**Table 6:** Healing properties and range of action of healing agents.

Healing Agent	Viscosity	Curing Time	Crack-Width Range	Strength Recovery	Durability Recovery
Alkaline silicate	General: At 20 °C, the viscosity is approximately 80 mPa·s	Often evaluated after 28 d curing; reaction rate depends on water availability and Ca(OH)_2_/Ca^2+^ supply	0.2–0.35 mm in TET/d-TET mini-vascular systems; 0.7–0.8 mm reported for Murray-law PLA vascular networks supplied with sodium silicate for 28 d	20% strength recovery for 1D/2D PLA vascular networks and 34% for a 3D PLA vascular network after 28 d sodium-silicate (SS) pumping; 17% for SS-filled d-TETs, increasing to 25% with SS/nanolime and 20% with SS/nanosilica	62% sealing efficiency in a glass fiber vascular comparison study; 25%, 69%, and 77% sorptivity reduction for 1D, 2D, and 3D PLA vascular networks, respectively
Epoxy resin	Approximately 80–500 mPa·s depending on formulation; 160 mPa·s reported for a two-component epoxy in a vascular-network study	Formulation-dependent; two-component systems can cure without elevated temperature; curing shorter than 1 min is unsuitable for pumping	≤0.5 mm in pressure-injected epoxy networks; 0.35 mm in d-TET systems	Full strength recovery in pressure-injected networks; flexural load recovery > 150% in ABS vascular systems and >100% in first-cycle PVA systems, whereas no clear recovery was obtained for insufficiently mixed d-TET epoxy	100% watertightness recovery in ABS networks; high recovery in PVA systems, but cured epoxy blocked the channels and generally limited repair to one effective cycle
Polyurethane	Broad formulation-dependent range; ~390–4200 mPa·s in a vascular-network study and up to ~7200 mPa·s in earlier self-healing literature	Quickly foams and hardens upon exposure to moisture; curing time depends on the formulation and humidity	Complete filling for 0.10–0.20 mm cracks; sealing efficiency exceeded 98% at 0.20 mm and remained above 74% for cracks up to 0.40 mm (Pickering emulsion in situ printing)	For 1–3 mm channels, flexural strength recovery was 12.24–17.61% after 1 d and increased to 32.49–39.48% after 3 d	98% sealing efficiency at 0.20 mm and >74% over the investigated 0.10–0.40 mm range; narrow cracks showed nearly complete water blocking
Cyanoacrylate	Very low viscosity, typically less than 10 mPa·s; according to reports, the viscosity of a CA system for vascular use is approximately 4 mPa·s	The polymerization reaction is very rapid, and the bond strength increases gradually over a period of 14 to 21 days.	Significant healing was reported up to approximately 0.20 mm, with a healing index > 100% at CMOD = 0.15 mm; ethyl cyanoacrylate has also been applied to cracks < 0.5 mm.	Strength recovery reached up to 90%; modified cyanoacrylate increased flexural-load recovery by 24% relative to commercial cyanoacrylate and by 48% relative to the best original formulation.	Not consistently quantified; rapid crack sealing and adhesive bonding were reported, but comparable permeability-recovery data remain unavailable.
Methyl methacrylate	Very low viscosity, approximately 1 mPa·s; reportedly, MMA thickened with PMMA has a viscosity of approximately 34 mPa·s to improve crack retention	Fast free-radical polymerization; three-component systems can harden within 30 min	Crack-width range not consistently reported; early studies on linear channel healing used a deflection value of 0.5 mm rather than directly measured crack width.	Under deflection-controlled conditions, the flexural strength of beams repaired using the early linear channel method has reportedly reached as high as 130%.	Not consistently quantified; reports indicate the presence of dense, plexiglass-like crack fillers, but lack comparable data on permeability.

**Table 7:** Characterization methods for healing performance

Type	Test	Characterization	Disadvantages	References
Mechanical property recovery	Bending test	Vascular arrangement effects, healing agent performance	Sample size, load control methods, and loading rates can affect the resulting strength values	[154,155]
	Compression test	Cement density, vascular weakening effect	Relatively insensitive to local crack bonding and affected by end friction, sample size, and channel orientation	[64]
	Uniaxial tensile test	Bonding of the healing agent and cement	Sensitive to eccentric loading, gripping, crack localization, and sample moisture	[131,140,156]
	Microscopic hardness testing	Vascular wall reinforcement	Representative only locally, and subject to the scale-down effect, surface treatment, and heterogeneity	[64,157,158,159]
	Acoustic emission	Evaluate healing efficiency	Sensitive to background noise	[71,72,126,154]
	Ultrasonic pulse velocity	Proof of healing	Affected by the temperature and moisture conditions at the test site and samples	[71,72,160]
Durability recovery	Permeability test, Water absorption test	Healing agent diffusion effect	Influenced by crack connectivity, crack distribution, and boundary sealing conditions	[125,161]
	Rapid chloride ion penetration	Anti-ion permeation of healing agents	Unable to distinguish between chemical bonding and physical barriers	[162]
Visualization	Optical microscope	Adhesion between vascular-based systems and cement	Resolution affects observation quality	[22,97,163]
	X-CT	Visualization of the internal vascular	Limited by the maximum resolution of the instrument	[22,63,64,76,98,99]
	Neutron ray scanning	Visualization of water molecule distribution	Hydrogen interference, equipment scarcity	[147,164,165]
	SEM, EDS	Analysis of the morphology and distribution of healing products	Coverage area is limited; sample preparation may alter the polymer or crack morphology; compounds cannot be identified using energy-dispersive spectroscopy alone	[98,166]
Elemental composition	XRD, FTIR	Determining the composition of healing products	Limited when used alone; requires a database to determine the corresponding elements or chemical bonds of peaks.	[64,98]

## Data Availability

No new data were created or analyzed in this study.

## References

[1] Habert G., Miller S.A., John V.M., Provis J.L., Favier A., Horvath A., Scrivener K.L. (2020). Environmental Impacts and Decarbonization Strategies in the Cement and Concrete Industries. Nat. Rev. Earth Environ..

[2] Al Khaffaf I., Hawileh R.A., Sahoo S., Abdalla J.A., Kim J.H. (2025). Toward Carbon- Neutral Construction: A Review of Zero-Carbon Concrete. J. Build. Eng..

[3] Wang T., Ding D., Song H., Wu H., Niu Y. (2026). Research Progress on the Self-Healing Mechanism and Technology of Cracks in Cementitious Materials. J. Mater. Sci..

[4] Fernandez C.A., Correa M., Nguyen M.-T., Rod K.A., Dai G.L., Cosimbescu L., Rousseau R., Glezakou V.-A. (2021). Progress and Challenges in Self-Healing Cementitious Materials. J. Mater. Sci..

[5] Wu M., Johannesson B., Geiker M. (2012). A Review: Self-Healing in Cementitious Materials and Engineered Cementitious Composite as a Self-Healing Material. Constr. Build. Mater..

[6] Wen X., Pagcaliwagan A.M., Hou H., Yin X. (2025). Advancements in Self-Healing Concrete: Material Mechanisms, Durability Assessment, and Implications for Infrastructure Lifecycle Management. Alex. Eng. J..

[7] De Belie N., Gruyaert E., Al-Tabbaa A., Antonaci P., Baera C., Bajare D., Darquennes A., Davies R., Ferrara L., Jefferson T. (2018). A Review of Self-Healing Concrete for Damage Management of Structures. Adv. Mater. Inter..

[8] Galano S., Calabrese A., Asvapathanagul P., Shadravan S., Ly M., Flores D., Hernandez M., Rahmani M. (2025). Innovative Approaches to Enhancing Concrete Compressive Strength: An Extensive Investigation of Biochar-Embedded and Self-Repairing Techniques. J. Mater. Civ. Eng..

[9] Chang H., Guo Z., Chen C., Zhang H., Li Z., Wang X., Liu J. (2024). Mechanical Properties, Durability and Self-Healing Performance of Mortar Containing Double-Layer Capsules. Mater. Today Commun..

[10] Jiang L., Wu M., Du F., Chen D., Xiao L., Chen W., Du W., Ding Q. (2024). State-of-the-Art Review of Microcapsule Self-Repairing Concrete: Principles, Applications, Test Methods, Prospects. Polymers.

[11] Shao L., Feng P., Liu Q., Chen C., Cai Y., Xu G. (2023). A Review on Performance Improvement and Multi-Functionalization of Cement Composites Using Capsules. Constr. Build. Mater..

[12] Yıldırım M., Özhan H.B., Öz H.G., Öğüt H. (2025). Utilization of tea waste as a bacterial carrier in self-healing mortars. Arch. Civ. Mech. Eng..

[13] Bhutange S.P., Latkar M.V., Muhammad S. (2024). A review on the potential challenges in the application of biocementation in cement-based materials, possible solutions and way forward. Mater. Today Commun..

[14] Sharma T., Banerjee A., Nanthagopalan P. (2024). Probing the Abyss: Bacteria-Based Self-Healing in Cementitious Construction Materials—A Review. Constr. Build. Mater..

[15] Verma N., Satya Eswari J., Mahapatra C. (2025). Revolutionizing Concrete: Unveiling Bio-Concrete’s Advantages and Challenges in Self-Healing through Microbial-Induced Calcium Carbonate Precipitation. Sustain. Mater. Technol..

[16] Hermawan H., Riordan C., Van Der Sichel S., Lust L., Serna P., Palmer D., Al-Tabbaa A., Gruyaert E. (2024). Mix Design Optimisation of Self-Sealing Concrete Containing Microcapsules with Polyurethane Shell and Water Repellent Cargo. Dev. Built Environ..

[17] Su Y., Qu F., Zhang J., Zhang X. (2024). A New Bacteria-Based Self-Healing System Triggered by Sulfate Ion for Cementitious Material. J. Build. Eng..

[18] Dry C. (1994). Matrix Cracking Repair and Filling Using Active and Passive Modes for Smart Timed Release of Chemicals from Fibers into Cement Matrices. Smart Mater. Struct..

[19] Yen E., Mishra G., Iqbal M.I., Namakiaraghi P., Shields Y., Van Tittelboom K., De Belie N., Farnam Y. (2024). Recent Progress in Vascularization of Cementitious Composites: Fundamental Concepts, Strategies and Applications. Constr. Build. Mater..

[20] Shumal M., Saghafian M., Shirani E., Nili-Ahmadabadi M. (2024). A General Scaling Law of Vascular Tree: Optimal Principle of Bifurcations in Pulsatile Flow. J. Appl. Fluid Mech..

[21] Jessen E., Steinbach M.C., Debbaut C., Schillinger D. (2024). Branching Exponents of Synthetic Vascular Trees Under Different Optimality Principles. IEEE Trans. Biomed. Eng..

[22] Li Z., Souza L.R.D., Litina C., Markaki A.E., Al-Tabbaa A. (2020). A Novel Biomimetic Design of a 3D Vascular Structure for Self-Healing in Cementitious Materials Using Murray’s Law. Mater. Des..

[23] Wan Z., Zhang Y., Xu Y., Šavija B. (2023). Self-Healing Cementitious Composites with a Hollow Vascular Network Created Using 3D-Printed Sacrificial Templates. Eng. Struct..

[24] Bejan A. (2024). Vascular Flow Design and Predicting Evolution. Int. Commun. Heat. Mass. Transf..

[25] Bejan A. (1996). Street Network Theory of Organization in Nature. J. Advced Transp..

[26] Miguel A.F. (2016). Toward an Optimal Design Principle in Symmetric and Asymmetric Tree Flow Networks. J. Theor. Biol..

[27] Bejan A., Lorente S., Wang K.-M. (2006). Networks of Channels for Self-Healing Composite Materials. J. Appl. Phys..

[28] Wang K.-M., Lorente S., Bejan A. (2007). Vascularization with Grids of Channels: Multiple Scales, Loops and Body Shapes. J. Phys. D Appl. Phys..

[29] Adeli H., Yeh C. (1989). Perceptron Learning in Engineering Design. Comput. Aided Civ. Eng..

[30] Ramadan Suleiman A., Nehdi M. (2017). Modeling Self-Healing of Concrete Using Hybrid Genetic Algorithm–Artificial Neural Network. Materials.

[31] Wan Z., Chang Z., Xu Y., Šavija B. (2023). Optimization of Vascular Structure of Self-Healing Concrete Using Deep Neural Network (DNN). Constr. Build. Mater..

[32] Zhang W., Gai J., Zhang Z., Tang L., Liao Q., Ding Y. (2019). Double-DQN Based Path Smoothing and Tracking Control Method for Robotic Vehicle Navigation. Comput. Electron. Agric..

[33] Wan Z., Xu Y., Chang Z., Liang M., Šavija B. (2024). Automatic Enhancement of Vascular Configuration for Self-Healing Concrete through Reinforcement Learning Approach. Constr. Build. Mater..

[34] Zhou B., Cheng Q., Chen Z., Chen Z., Liang D., Munro E.A., Yun G., Kawai Y., Chen J., Bhowmick T. (2024). Universal Murray’s Law for Optimised Fluid Transport in Synthetic Structures. Nat. Commun..

[35] Skorpa R., Vrålstad T. (2018). Visualization of Fluid Flow Through Cracks and Microannuli in Cement Sheaths. SPE J..

[36] Medeiros J.M.P., Di Sarno L. (2024). Cracking Methods for Testing of Self-Healing Concrete: An Experimental Approach. Buildings.

[37] Garg A., Mishra H., Pattanayek S.K. (2025). Optimal Flow and Scaling Laws for Power-Law Fluids in Elliptical Cross-Sectional Self-Similar Tree-like Networks. J. Appl. Phys..

[38] Rathnayaka M., Wijesundara K., Gunasekara C., Law D.W., Karunasingha D., Lokuge W. (2025). Integrating Advanced Data Imputation Techniques and Machine Learning Models to Develop a Predictive Model for Compressive Strength of Geopolymer Concrete. Arch. Civ. Mech. Eng..

[39] Jamal A.S., Ahmed A.N. (2025). Estimating Compressive Strength of High-Performance Concrete Using Different Machine Learning Approaches. Alex. Eng. J..

[40] Shafaie V., Movahedi Rad M. (2024). Multi-Objective Genetic Algorithm Calibration of Colored Self-Compacting Concrete Using DEM: An Integrated Parallel Approach. Sci. Rep..

[41] Fang Z., Wang X., Sun Y., Adibhashimi M.A. (2025). Accelerating Multi-Objective Optimization of Concrete Thin Shell Structures Using Graph-Constrained GANs and NSGA-II. Sci. Rep..

[42] Khosravi H., Bahram M. (2025). Prediction of Geopolymer Concrete Compressive Strength Using Artificial Neural Network and Genetic Algorithm. Results Eng..

[43] Zhao Z., Bao Y., Gao T., An Q. (2024). Optimization of GFRP-Concrete-Steel Composite Column Based on Genetic Algorithm—Artificial Neural Network. Appl. Ocean. Res..

[44] Bäck T.H.W., Kononova A.V., Van Stein B., Wang H., Antonov K.A., Kalkreuth R.T., De Nobel J., Vermetten D., De Winter R., Ye F. (2023). Evolutionary Algorithms for Parameter Optimization—Thirty Years Later. Evol. Comput..

[45] Zantvoort K., Nacke B., Görlich D., Hornstein S., Jacobi C., Funk B. (2024). Estimation of Minimal Data Sets Sizes for Machine Learning Predictions in Digital Mental Health Interventions. npj Digit. Med..

[46] Xu P., Ji X., Li M., Lu W. (2023). Small Data Machine Learning in Materials Science. npj Comput. Mater..

[47] Antipov D., Doerr B. (2024). Evolutionary Algorithms Are Significantly More Robust to Noise When They Ignore It. arXiv.

[48] Jiang W., Gao K., Zhu S., Xu L. (2025). A Novel Robust Multi-Objective Evolutionary Optimization Algorithm Based on Surviving Rate. Complex. Intell. Syst..

[49] Bos F., Menna C., Robens-Radermacher A., Wolfs R., Roussel N., Lombois-Burger H., Baz B., Weger D., Nematollahi B., Santhanam M. (2025). Mechanical Properties of 3D Printed Concrete: A RILEM TC 304-ADC Interlaboratory Study—Approach and Main Results. Mater. Struct..

[50] Tao Q., Yu J., Mu X., Jia X., Shi R., Yao Z., Wang C., Zhang H., Liu X. (2025). Machine Learning Strategies for Small Sample Size in Materials Science. Sci. China Mater..

[51] Liu S., Wang H., Peng W., Yao W. (2024). Surrogate-Assisted Evolutionary Algorithms for Expensive Combinatorial Optimization: A Survey. Complex. Intell. Syst..

[52] Jayasinghe S.C., Mahmoodian M., Alavi A., Sidiq A., Shahrivar F., Sun Z., Thangarajah J., Setunge S. (2025). A Review on the Applications of Artificial Neural Network Techniques for Accelerating Finite Element Analysis in the Civil Engineering Domain. Comput. Struct..

[53] Li K., Rubungo A.N., Lei X., Persaud D., Choudhary K., DeCost B., Dieng A.B., Hattrick-Simpers J. (2025). Probing Out-of-Distribution Generalization in Machine Learning for Materials. Commun. Mater..

[54] Miao X., Chen T., Lang Z., Wu Y., Wu X., Zhu Z., Xu R.X. (2025). Design, Fabrication, and Application of Bioengineering Vascular Networks Based on Microfluidic Strategies. J. Mater. Chem. B.

[55] Mustafaoglu M., Kaan Yesilyurt M., Kotcioglu İ., Allahyari M. (2025). Analysis of Flow Resistance in Trifurcation Fluid Networks. JAFM.

[56] Cruz A.S., Caldas L.R., Mendes V.M., Mendes J.C., Bastos L.E.G. (2024). Multi-Objective Optimization Based on Surrogate Models for Sustainable Building Design: A Systematic Literature Review. Build. Environ..

[57] Gupta R., Zhang Q. (2024). Data-driven Decision-focused Surrogate Modeling. AIChE J..

[58] Peng F., Li Y., Xue W. (2024). Transfer Learning-Based Confinement Strength Prediction of Concrete Confined by FRP Transverse Reinforcements. Eng. Struct..

[59] Ajani O.S., Ivan D.F., Darlan D., Suganthan P.N., Gao K., Mallipeddi R. (2024). Deep Reinforcement Learning as Multiobjective Optimization Benchmarks: Problem Formulation and Performance Assessment. Swarm Evol. Comput..

[60] Gao Q., Schweidtmann A.M. (2024). Deep Reinforcement Learning for Process Design: Review and Perspective. Curr. Opin. Chem. Eng..

[61] Muhit I.B., Al-Fakih A., Suntharalingam T., Michel A. (2025). Progress in Computational Modelling for Concrete Durability and Its Integration with Artificial Intelligence and Life-Cycle Assessment. Arch. Comput. Methods Eng..

[62] Pareek S., Shrestha K.C., Suzuki Y., Omori T., Kainuma R., Araki Y. (2014). Feasibility of Externally Activated Self-Repairing Concrete with Epoxy Injection Network and Cu-Al-Mn Superelastic Alloy Reinforcing Bars. Smart Mater. Struct..

[63] Wan Z., Xu Y., Zhang Y., He S., Šavija B. (2022). Mechanical Properties and Healing Efficiency of 3D-Printed ABS Vascular Based Self-Healing Cementitious Composite: Experiments and Modelling. Eng. Fract. Mech..

[64] Zhang Y., Lu P., Fang G., Dong B., Hong S., Wang Y., Li J., Fan S. (2024). Enhancing Mechanical Properties of Concrete with 3D Printed Vascular Networks via Carbonation Strengthening. Cem. Concr. Compos..

[65] Lee H.W., Meng L., Ashkpour A., Sadighi A., Iqbal M.I., Pour-Ghaz M., Hubler M.H., Sales C.M., Farnam Y., Najafi A.R. (2025). Sensitivity of Embedded Channel for Self-Healing Agent Delivery on Splitting Tensile Strength of Concrete. J. Build. Eng..

[66] Qian H., Umar M., Nasir Ayaz Khan M., Shi Y., Manan A., Raza A., Li F., Li Z., Chen G. (2024). A State-of-the-Art Review on Shape Memory Alloys (SMA) in Concrete: Mechanical Properties, Self-Healing Capabilities, and Hybrid Composite Fabrication. Mater. Today Commun..

[67] Kuang Y., Ou J. (2008). Self-Repairing Performance of Concrete Beams Strengthened Using Superelastic SMA Wires in Combination with Adhesives Released from Hollow Fibers. Smart Mater. Struct..

[68] Kuang Y., Ou J. (2008). Passive Smart Self-Repairing Concrete Beams by Using Shape Memory Alloy Wires and Fibers Containing Adhesives. J. Cent. South. Univ. Technol..

[69] Shields Y., Van Mullem T., De Belie N., Van Tittelboom K. (2021). An Investigation of Suitable Healing Agents for Vascular-Based Self-Healing in Cementitious Materials. Sustainability.

[70] Wan Z., Xu Y., He S., Schlangen E., Šavija B. (2024). The Use of Additive Manufacturing in Self-Healing Cementitious Materials: A State-of-the-Art Review. Dev. Built Environ..

[71] Tsangouri E., Karaiskos G., Deraemaeker A., Van Hemelrijck D., Aggelis D. (2016). Assessment of Acoustic Emission Localization Accuracy on Damaged and Healed Concrete. Constr. Build. Mater..

[72] Tsangouri E., Van Loo C., Shields Y., De Belie N., Van Tittelboom K., Aggelis D.G. (2022). Reservoir-Vascular Tubes Network for Self-Healing Concrete: Performance Analysis by Acoustic Emission, Digital Image Correlation and Ultrasound Velocity. Appl. Sci..

[73] Zhang Y., Pan P., Li W., Dong B., Tang J., Xing F., Zhu G.M. (2022). Freeform Embedded Printing of Vasculature in Cementitious Materials for Healing-Agent Transport. Addit. Manuf..

[74] Zhang Y., Li W., Pan P., Tang J., Dong B., Xing F., Zhu G. (2022). Programmable Construction of Vasculature by Printing in Cementitious Materials for Self-Healing Application. Compos. Part B Eng..

[75] Sharma D., Goyal S. (2022). Effect of Accelerated Carbonation Curing on near Surface Properties of Concrete. Eur. J. Environ. Civ. Eng..

[76] Wan Z., Xu Y., He S., Chen Y., Xie J., Šavija B. (2023). Direct Ink Writing of Vascularized Self-Healing Cementitious Composites. Cem. Concr. Compos..

[77] Yuan J., Liu J., Zhao J., Song K., Zhang Q. (2025). Coaxially Printing of Biomimetic Self-Healing and Transparent Silicate-Epoxy Composites. Case Stud. Constr. Mater..

[78] Li V.C., Lim Y.M., Chan Y.-W. (1998). Feasibility Study of a Passive Smart Self-Healing Cementitious Composite. Compos. Part B Eng..

[79] Dry C., McMillan W. (1996). Three-Part Methylmethacrylate Adhesive System as an Internal Delivery System for Smart Responsive Concrete. Smart Mater. Struct..

[80] Qian H., Wang X., Wang Y., Duan S., Xiong J. (2024). Experimental Investigation on Flexural Performance of Concrete Beams Strengthened with SMA-GFRP-ECC Smart Composite Materials. J. Intell. Mater. Syst. Struct..

[81] Chen G., Tang W., Chen S., Ng C., Cui H. (2025). Prediction of Self-Healing Ability of Recurring Cracks in Engineered Cementitious Composites with a Machine Learning Based Computational Approach. J. Build. Eng..

[82] Turkmen M., Issa A., Awayssa O., El-Hassan H. (2025). Incorporation of Nitinol (NiTi) Shape Memory Alloy (SMA) in Concrete: A Review. Materials.

[83] Sun W., Wu Y., Yuan J., Gao X., Lu S., Ni W., Feng J. (2025). Optimizing Penetration Resistance of Ni-Ti Shape Memory Alloy Fiber Reinforced Concrete under High Impact Velocity: A Comprehensive Study. J. Build. Eng..

[84] Nishiwaki T., Mihashi H., Jang B.-K., Miura K. (2006). Development of Self-Healing System for Concrete with Selective Heating around Crack. J. Adv. Concr. Technol..

[85] Jang D., Park J., Choi S., Bang J., Choi J., Kim J., Yang B., Jeon H. (2025). Novel Approach for Crack Detections and Rapid Repairment Methods in Cement-Based Self-Heating Composites for Smart Infrastructures. Compos. Part B Eng..

[86] Oumer A., Lee C., Ahn E., Gwon S. (2024). Review on Self-Heating Electrically Conductive Cementitious Composites: Focus on Deicing and Electrical Curing. Constr. Build. Mater..

[87] Ukpata J.O., Ewa D.E., Liwhuliwhe J.U., Alaneme G.U., Obeten K.E. (2023). Effects of Elevated Temperatures on the Mechanical Properties of Laterized Concrete. Sci. Rep..

[88] Zhang L., Zheng M., Zhao D., Feng Y. (2024). A Review of Novel Self-Healing Concrete Technologies. J. Build. Eng..

[89] Wan Z., Xu Y., Šavija B. (2023). Influence of Printing Direction on 3D-Printed Vascular Based Self-Healing Cementitious Composites. MATEC Web Conf..

[90] Davies R., Jefferson A., Lark R., Gardner D. A Novel 2D Vascular Network in Cementitious Materials. Proceedings of the Symposium 2015.

[91] Davies R., Teall O., Pilegis M., Kanellopoulos A., Sharma T., Jefferson A., Gardner D., Al-Tabbaa A., Paine K., Lark R. (2018). Large Scale Application of Self-Healing Concrete: Design, Construction, and Testing. Front. Mater..

[92] Van Tittelboom K., De Belie N. (2013). Self-Healing in Cementitious Materials—A Review. Materials.

[93] Anglani G., Van Mullem T., Tulliani J.-M., Van Tittelboom K., De Belie N., Antonaci P. (2022). Durability of Self-Healing Cementitious Systems with Encapsulated Polyurethane Evaluated with a New Pre-Standard Test Method. Mater. Struct..

[94] Dwiyati S.T., Kholil A., Riyadi R., Putra S.E. (2019). Influence of Layer Thickness and 3D Printing Direction on Tensile Properties of ABS Material. J. Phys. Conf. Ser..

[95] Ni F., Wang G., Zhao H. (2017). Fabrication of Water-soluble Poly(Vinyl Alcohol)-based Composites with Improved Thermal Behavior for Potential Three-dimensional Printing Application. J. Appl. Polym. Sci..

[96] De Nardi C., Gardner D., Jefferson A.D. (2020). Development of 3D Printed Networks in Self-Healing Concrete. Materials.

[97] De Nardi C., Gardner D., Cristofori D., Ronchin L., Vavasori A., Jefferson T. (2023). Advanced 3D Printed Mini-Vascular Network for Self-Healing Concrete. Mater. Des..

[98] Li Z., Souza L.R.D., Litina C., Markaki A.E., Al-Tabbaa A. (2019). Feasibility of Using 3D Printed Polyvinyl Alcohol (PVA) for Creating Self-Healing Vascular Tunnels in Cement System. Materials.

[99] Osan R., Deb R., Houshmand M., Namakiaraghi P., Iqbal M.I., Farnam Y.A. (2025). Nature Inspired Vascular Self-Thermal Responsive Cementitious Composites with Phase Change Materials. J. Build. Eng..

[100] Lefever G., Abdullah A., Van Hemelrijck D., Snoeck D., Aggelis D.G. (2024). Air-Coupled Ultrasonic Mapping of Stimulated Autogenous Self-Healing and Repair Effectiveness within Concrete Mixtures. Constr. Build. Mater..

[101] Zhang Y., Zhu G., Dong B., Wang F., Tang J., Stadler F.J., Yang G., Hong S., Xing F. (2021). Interfacial Jamming Reinforced Pickering Emulgel for Arbitrary Architected Nanocomposite with Connected Nanomaterial Matrix. Nat. Commun..

[102] Cui S.-M., Hashmi S., Li W.-Q., Handschuh-Wang S., Zhu C.-T., Wang S.-C., Huang Y.-F., Zhu G.-M., Stadler F.J. (2023). Rheology of Graphene Oxide Stabilized Pickering Emulsions. Soft Matter.

[103] Karvinen J., Kellomäki M. (2024). 3D-Bioprinting of Self-Healing Hydrogels. Eur. Polym. J..

[104] Lim H.-P., Karandagaspitiya C.O., Chan D.K.-H., Low L.-E., Tey B.-T., Chan E.-S. (2023). Pickering Emulsion Ink in Additive Manufacturing: A State-of-the-Art Review. Addit. Manuf..

[105] Huang H., Liao L., Lin Z., Pan D., Nuo Q., Wu T., Jiang Y., Bai H. (2023). Direct Ink Writing of Pickering Emulsions Generates Ultralight Conducting Polymer Foams with Hierarchical Structure and Multifunctionality. Small.

[106] Wang X., Zheng J., Zhang D., Zhang X., Zhu G., Zhang Y., Dong B., Luo Q., Long W., Xing F. (2026). Pickering Emulsion-Based 3D Printing for Efficient Vascular Self-Healing in Cementitious Materials. Cem. Concr. Compos..

[107] Hojati M., Sedghi R., Li Z., Memari A., Nazarian S., Radlińska A., Duarte J.P., Lowke D., Freund N., Böhler D., Herding F. (2024). Flexural Strength of 3D Printed Concrete Beams: Exploring Barbed-Wire Reinforcement and Cross-Sectional Geometry. Fourth RILEM International Conference on Concrete and Digital Fabrication.

[108] Saadi M.A.S.R., Maguire A., Pottackal N.T., Thakur M.S.H., Ikram M.M., Hart A.J., Ajayan P.M., Rahman M.M. (2022). Direct Ink Writing: A 3D Printing Technology for Diverse Materials. Adv. Mater..

[109] El-Sayegh S., Romdhane L., Manjikian S. (2020). A Critical Review of 3D Printing in Construction: Benefits, Challenges, and Risks. Arch. Civ. Mech. Eng..

[110] Rahman M., Rawat S., Yang R., Mahil A., Zhang Y.X. (2024). A Comprehensive Review on Fresh and Rheological Properties of 3D Printable Cementitious Composites. J. Build. Eng..

[111] Zhou Y., Althoey F., Alotaibi B.S., Gamil Y., Iftikhar B. (2023). An Overview of Recent Advancements in Fibre-Reinforced 3D Printing Concrete. Front. Mater..

[112] Tang J., Wang Z., Gao D., Yang L., Zhu H., Zhao L., Li Z. (2025). Research Progress on 3D Printed Geopolymer Concrete. J. Mater. Sci..

[113] Raza M.H., Besklubova S., Zhong R.Y. (2024). Economic Analysis of Offsite and Onsite 3D Construction Printing Techniques for Low-Rise Buildings: A Comparative Value Stream Assessment. Addit. Manuf..

[114] Wang X., Li W., Guo Y., Kashani A., Wang K., Ferrara L., Agudelo I. (2024). Concrete 3D Printing Technology for Sustainable Construction: A Review on Raw Material, Concrete Type and Performance. Dev. Built Environ..

[115] Miri Z.S., Baaj H., Polak M.A. (2025). 3D-Printed Concrete Bridges: Material, Design, Construction, and Reinforcement. Appl. Sci..

[116] Adhikary S.K., Rathod N., Adhikary S.D., Kumar A., Perumal P. (2024). Chemical-Based Self-Healing Concrete: A Review. Discov. Civ. Eng..

[117] Ait Ouarabi M., Antonaci P., Boubenider F., Gliozzi A., Scalerandi M. (2017). Ultrasonic Monitoring of the Interaction between Cement Matrix and Alkaline Silicate Solution in Self-Healing Systems. Materials.

[118] Yang X., Zhu W., Yang Q. (2008). The Viscosity Properties of Sodium Silicate Solutions. J. Solut. Chem..

[119] Joseph C., Jefferson A.D., Cantoni M.B. Issues Relating to the Autonomic Healing of Cementitious Materials. Proceedings of the First International Conference on Self-Healing Materials.

[120] Rumman R., Bediwy A., Alam M.S. (2024). Revolutionizing Concrete Durability: Case Studies on Encapsulation- Based Chemical (Autonomous) Self-Healing Techniques and Future Directions—A Critical Review. Case Stud. Constr. Mater..

[121] Amran M., Onaizi A.M., Fediuk R., Vatin N.I., Muhammad Rashid R.S., Abdelgader H., Ozbakkaloglu T. (2022). Self-Healing Concrete as a Prospective Construction Material: A Review. Materials.

[122] Dry C., Corsaw M., Bayer E. (2003). A Comparison of Internal Self-Repair with Resin Injection in Repair of Concrete. J. Adhes. Sci. Technol..

[123] Abdulqadir Z.M., Karim F.R. (2022). Evaluating the Efficiency of Epoxy Injection Technique for Repairing Normal and High Strength Concrete Beams—A Critical Review. Constr..

[124] Ighalo J.O., Kurniawan S.B., Khongthaw B., Buhari J., Chauhan P.K., Georgin J., Pfingsten Franco D.S. (2024). Bisphenol A (BPA) Toxicity Assessment and Insights into Current Remediation Strategies. RSC Adv..

[125] Van Tittelboom K., De Belie N., Van Loo D., Jacobs P. (2011). Self-Healing Efficiency of Cementitious Materials Containing Tubular Capsules Filled with Healing Agent. Cem. Concr. Compos..

[126] Van Tittelboom K., De Belie N., Lehmann F., Grosse C.U. (2012). Acoustic Emission Analysis for the Quantification of Autonomous Crack Healing in Concrete. Constr. Build. Mater..

[127] Hu Z.-X., Hu X.-M., Cheng W.-M., Zhao Y.-Y., Wu M.-Y. (2018). Performance Optimization of One-Component Polyurethane Healing Agent for Self-Healing Concrete. Constr. Build. Mater..

[128] Van Belleghem B., Gruyaert E., Van Tittelboom K., Moerman W., Dekeyser B., Van Stappen J., Cnudde V., De Belie N. (2018). Effect of Polyurethane Viscosity on Self-Healing Efficiency of Cementitious Materials Exposed to High Temperatures from Sun Radiation. J. Mater. Civ. Eng..

[129] Davies R., Jefferson T., Gardner D. (2021). Development and Testing of Vascular Networks for Self-Healing Cementitious Materials. J. Mater. Civ. Eng..

[130] De Nardi C., Gardner D., Cazzador G., Cristofori D., Ronchin L., Vavasori A., Jefferson T. (2021). Experimental Investigation of a Novel Formulation of a Cyanoacrylate-Based Adhesive for Self-Healing Concrete Technologies. Front. Built Environ..

[131] De Nardi C., Freeman B.L., Gardner D., Jefferson T. (2023). Mechanical Response and Predictive Modelling of Vascular Self-Healing Cementitious Materials Using Novel Healing Agents. Cem. Concr. Compos..

[132] Yip B.F., Kasiman E.H., Zainal Abidin A.R., Tan C.S., Sulaiman A. (2025). Improving Capillary Flow Predictions in Self-Healing Concrete: A Dynamic Contact Angle Approach Using the Volume of Fluid Model. J. Build. Rehabil..

[133] Vouvoudi E.C., Morfis P.D., Chatzicharistou E.A., Tamias G.A., Achilias D.S. (2022). Preliminary Study of a Polycyanoacrylate Adhesive for Glass: Changes Under Ageing Conditions. Sci. Cult..

[134] Kaptay G. (2024). On the Temperature Dependence of Surface Tension: Historical Perspective on the Eötvös Equation of Capillarity, Celebrating His 175th Anniversary. Adv. Colloid. Interface Sci..

[135] Dehghanpoor Abyaneh S., Wong H.S., Buenfeld N.R. (2014). Computational Investigation of Capillary Absorption in Concrete Using a Three-Dimensional Mesoscale Approach. Comput. Mater. Sci..

[136] Gardner D., Jefferson A., Hoffman A., Lark R. (2014). Simulation of the Capillary Flow of an Autonomic Healing Agent in Discrete Cracks in Cementitious Materials. Cem. Concr. Res..

[137] Huang H., Ye G., Shui Z. (2014). Feasibility of Self-Healing in Cementitious Materials – By Using Capsules or a Vascular System?. Constr. Build. Mater..

[138] Hamilton A.R., Sottos N.R., White S.R. (2012). Pressurized Vascular Systems for Self-Healing Materials. J. R. Soc. Interface.

[139] Qamar I.P.S., Sottos N.R., Trask R.S. (2020). Grand Challenges in the Design and Manufacture of Vascular Self-Healing. Multifunct. Mater..

[140] Selvarajoo T., Davies R.E., Freeman B.L., Jefferson A.D. (2020). Mechanical Response of a Vascular Self-Healing Cementitious Material System under Varying Loading Conditions. Constr. Build. Mater..

[141] Zirjawi A.S., Xue P., Chaudhry S.H. (2025). Enhanced Method in the Design of Microvascular Self-Healing System in a Composite Structure Based on a Newtonian Healing Agent. J. Mater. Res. Technol..

[142] Freeman B.L., Jefferson T. (2020). The Simulation of Transport Processes in Cementitious Materials with Embedded Healing Systems. Numer. Anal. Methods Geomech..

[143] Mohan P. (2013). A Critical Review: The Modification, Properties, and Applications of Epoxy Resins. Polym.-Plast. Technol. Eng..

[144] Xiang Q., Xiao F. (2020). Applications of Epoxy Materials in Pavement Engineering. Constr. Build. Mater..

[145] Wang X., Xie W., Ren J., Zhu J., Li L.-Y., Xing F. (2021). Interfacial Binding Energy between Calcium-Silicate-Hydrates and Epoxy Resin: A Molecular Dynamics Study. Polymers.

[146] Sun D., Wang J., Wang H., Yan Z., Ma W., Ma X., Wang Y., Ma Z., Jin X., Sun S. (2023). Influence of Crosslink Density on the Interfacial Bonding Performance for Epoxy Resin towards Load-Induced Calcium Silicate Hydrate Gel. J. Non-Cryst. Solids.

[147] Van Den Heede P., Van Belleghem B., Alderete N., Van Tittelboom K., De Belie N. (2016). Neutron Radiography Based Visualization and Profiling of Water Uptake in (Un)Cracked and Autonomously Healed Cementitious Materials. Materials.

[148] Joseph C., Jefferson A.D., Isaacs B., Lark R., Gardner D. (2010). Experimental Investigation of Adhesive-Based Self-Healing of Cementitious Materials. Mag. Concr. Res..

[149] Van Tittelboom K., Adesanya K., Dubruel P., Van Puyvelde P., De Belie N. (2011). Methyl Methacrylate as a Healing Agent for Self-Healing Cementitious Materials. Smart Mater. Struct..

[150] Kan Y.-C., Yen T., Lee M.-G. (2008). Restored Strength of Cracked Concrete Beam Repaired by Epoxy and Polymethyl Methacrylate. ACI Mater. J..

[151] Giannaros P., Kanellopoulos A., Al-Tabbaa A. (2016). Sealing of Cracks in Cement Using Microencapsulated Sodium Silicate. Smart Mater. Struct..

[152] Kanellopoulos A., Giannaros P., Al-Tabbaa A. (2016). The Effect of Varying Volume Fraction of Microcapsules on Fresh, Mechanical and Self-Healing Properties of Mortars. Constr. Build. Mater..

[153] Hu M., Guo J., Xu Y., Fan J., Cao L., Wang M., Feng Y. (2018). Influence of Triethanolamine on Reactivity of Hydrated Matrix in Sodium Silicate Self-Healing System and the Mechanism. Constr. Build. Mater..

[154] Granger S., Loukili A., Pijaudier-Cabot G., Chanvillard G. (2007). Experimental Characterization of the Self-Healing of Cracks in an Ultra High Performance Cementitious Material: Mechanical Tests and Acoustic Emission Analysis. Cem. Concr. Res..

[155] Hilloulin B., Hilloulin D., Grondin F., Loukili A., De Belie N. (2016). Mechanical Regains Due to Self-Healing in Cementitious Materials: Experimental Measurements and Micro-Mechanical Model. Cem. Concr. Res..

[156] Jefferson A., Selvarajoo T., Freeman B., Davies R. (2019). An Experimental and Numerical Study on Vascular Self-Healing Cementitious Materials. MATEC Web Conf..

[157] Glinicki M.A., Zielinski M. (2004). Depth-Sensing Indentation Method for Evaluation of Efficiency of Secondary Cementitious Materials. Cem. Concr. Res..

[158] Igarashi S., Bentur A., Mindess I.S. (1996). Microhardness Testing of Cementitious Materials. Adv. Cem. Based Mater..

[159] Ollivier J.P., Maso J.C., Bourdette B. (1995). Interfacial Transition Zone in Concrete. Adv. Cem. Based Mater..

[160] Ahn E., Kim H., Sim S.-H., Shin S., Shin M. (2017). Principles and Applications of Ultrasonic-Based Nondestructive Methods for Self-Healing in Cementitious Materials. Materials.

[161] Hou S., Li K., Wu Z., Li F., Shi C. (2022). Quantitative Evaluation on Self-Healing Capacity of Cracked Concrete by Water Permeability Test—A Review. Cem. Concr. Compos..

[162] Hameed N.A., Othman F.M., Abdul-Hamead A.A. (2023). Integration of FDM and Self-Healing Technology: Evaluation of Crack Sealing by Durability and Mechanical Strength. Mater. Res. Express.

[163] Van Tittelboom K., Wang J., Araújo M., Snoeck D., Gruyaert E., Debbaut B., Derluyn H., Cnudde V., Tsangouri E., Van Hemelrijck D. (2016). Comparison of Different Approaches for Self-Healing Concrete in a Large-Scale Lab Test. Constr. Build. Mater..

[164] Van Tittelboom K., Snoeck D., Vontobel P., Wittmann F.H., De Belie N. (2013). Use of Neutron Radiography and Tomography to Visualize the Autonomous Crack Sealing Efficiency in Cementitious Materials. Mater. Struct..

[165] Zhang P., Wittmann F.H., Lura P., Müller H.S., Han S., Zhao T. (2018). Application of Neutron Imaging to Investigate Fundamental Aspects of Durability of Cement-Based Materials: A Review. Cem. Concr. Res..

[166] Wang X.F., Yang Z.H., Fang C., Han N.X., Zhu G.M., Tang J.N., Xing F. (2019). Evaluation of the Mechanical Performance Recovery of Self-Healing Cementitious Materials—Its Methods and Future Development: A Review. Constr. Build. Mater..

[167] Shields Y., Di Summa D., Ospitia N., Herrier G., Schlangen E., Jefferson T., De Belie N., Van Tittelboom K. (2024). A Large-Scale Demonstration and Sustainability Evaluation of Ductile-Porous Vascular Networks for Self-Healing Concrete. J. Build. Eng..

[168] Shields Y., Tsangouri E., Riordan C., De Nardi C., Godinho J.R.A., Ooms T., Antonaci P., Palmer D., Al-Tabbaa A., Jefferson T. (2024). Non-Destructive Evaluation of Ductile-Porous versus Brittle 3D Printed Vascular Networks in Self-Healing Concrete. Cem. Concr. Compos..

[169] Sidiq A., Robert D., Gravina R., Giustozzi F. (2021). Coupled FEM-Microstructural X-Ray Examination of a Controlled Internal Damage Approach for Concrete Samples. Arch. Civ. Mech. Eng..

[170] Bai Z., Jiang J., Zhu H., Zhang D., Zhang H., Yu Y., Quan F. (2024). Multi-Network PVA/SA Aerogel Fiber: Unveiling Superior Mechanical, Thermal Insulation, and Extreme Condition Stability. Polym. Degrad. Stab..

[171] Bhagoria P., Bharadwaj M.R., Kumar R., Yadav K., Tiwari V. (2025). Mechanical and Fracture Behaviour of Epoxy Polymer under Extreme Low Temperature and High Strain Rate Conditions. Int. J. Adhes. Adhes..

[172] Xu Y., Ye H., Yuan Q., Shi C., Gao Y., Fu Q. (2022). The Durability of Concrete Subject to Mechanical Load Coupled with Freeze–Thaw Cycles: A Review. Arch. Civ. Mech. Eng..

[173] Zuo Z., De Corte W., Huang Y., Chen X., Zhang Y., Li J., Zhang L., Xiao J., Yuan Y., Zhang K. (2024). Strategies towards Large-Scale 3D Printing without Size Constraints. Virtual Phys. Prototyp..

[174] Zuo Z., De Corte W., Huang Y., Chen X., Zhang Y., Li J., Zhang L., Xiao J., Yuan Y., Zhang K. (2023). Propelling the Widespread Adoption of Large-Scale 3D Printing. Nat. Rev. Mater..

[175] Geng S.-Y., Cheng B.-Y., Long W.-J., Luo Q.-L., Dong B.-Q., Xing F. (2025). Co-Driven Physics and Machine Learning for Intelligent Control in High-Precision 3D Concrete Printing. Autom. Constr..

[176] Geng S., Luo Q., Liu K., Li Y., Hou Y., Long W. (2023). Research Status and Prospect of Machine Learning in Construction 3D Printing. Case Stud. Constr. Mater..

[177] Gao H., Jin L., Chen Y., Chen Q., Liu X., Yu Q. (2024). Rheological Behavior of 3D Printed Concrete: Influential Factors and Printability Prediction Scheme. J. Build. Eng..

[178] Ma L., Yin D., Ren J., Tian M., Chen X., Li L. (2024). An Effective Thixotropic Structural Dynamics Rheological Model for 3D Printed Concrete Materials in the Flow State. Constr. Build. Mater..

[179] Tang J., Gao C., Li Y., Xu J., Huang J., Xu D., Hu Z., Han F., Liu J. (2024). A Review on Multi-Scale Toughening and Regulating Methods for Modern Concrete: From Toughening Theory to Practical Engineering Application. Research.

[180] Senkans U., Silkans N., Merijs-Meri R., Haritonovs V., Skels P., Porins J., Lima M.S.S., Spolitis S., Braunfelds J., Bobrovs V. (2026). Fiber-Optical-Sensor-Based Technologies for Future Smart-Road-Based Transportation Infrastructure Applications. Photonics.

[181] Sun Z., Mahmoodian M., Sidiq A., Jayasinghe S., Shahrivar F., Setunge S. (2025). Optimal Sensor Placement for Structural Health Monitoring: A Comprehensive Review. J. Sens. Actuator Netw..

[182] Sarkar K., Shiuly A., Dhal K.G. (2024). Revolutionizing Concrete Analysis: An in-Depth Survey of AI-Powered Insights with Image-Centric Approaches on Comprehensive Quality Control, Advanced Crack Detection and Concrete Property Exploration. Constr. Build. Mater..

[183] Kato Y., Minakuchi S., Ogihara S., Takeda N. (2021). Self-Healing Composites Structure Using Multiple through-Thickness Microvascular Channels. Adv. Compos. Mater..

[184] Zhu J., Song J. (2020). Weakly Supervised Network Based Intelligent Identification of Cracks in Asphalt Concrete Bridge Deck. Alex. Eng. J..

[185] Ling Q., Goyal M., Harrison M.D., Hassanpour M. (2025). Rice Husk Derived Lignin Particles Generated through Solvent and pH Shifting for Pickering Emulsion Applications. Ind. Crops Prod..

[186] Chen Q., Zhao Y., Yang Z., Guo R., Huan S., Wang B., Yang G. (2024). Pickering Emulsion via Interfacial Assembly of Lignin Particles and Cationic Surfactant: Formation of Robust Anchoring Layer. Colloids Surf. A Physicochem. Eng. Asp..

[187] Feng J., Qian S. (2023). Accelerating Autonomic Healing of Cementitious Composites by Using Nano Calcium Carbonate Coated Polypropylene Fibers. Mater. Des..

[188] Taheri S., Georgaklis J., Ams M., Patabendigedara S., Belford A., Wu S. (2022). Smart Self-Sensing Concrete: The Use of Multiscale Carbon Fillers. J. Mater. Sci..

